# Progressively exploring and assessing the prognosis of bladder urothelial cancer based on the microenvironment through the integration of multiple databases

**DOI:** 10.3389/fmolb.2025.1702311

**Published:** 2025-11-19

**Authors:** Xiong Zou, Yanfeng Li, Xuefeng Peng, Changshi Gu, Qiang Wang

**Affiliations:** Department of Urology, The Affiliated Hospital of Guizhou Medical University, Guiyang, Guizhou, China

**Keywords:** bladder urothelial carcinoma (BLCA), risk stratification, immune subtypes, prognosis, microenvironment

## Abstract

**Background:**

The heterogeneous prognosis of bladder urothelial carcinoma (BLCA) remains a significant clinical challenge. A multi-factor prognostic model is essential for BLCA, as it not only assesses tumor progression and elucidates underlying molecular mechanisms but also paves the way for timely treatment adjustments and improved clinical decision-making.

**Methods:**

Using R software, we performed immunophenotyping on multiple BLCA cohorts from the GEO database to identify shared immune signatures. Simultaneously, we identified BLCA prognosis-associated genes by analyzing TCGA data. Prognostic genes were further refined via LASSO regression, allowing BLCA patients to be stratified into high- and low-risk groups based on their expression patterns. Quantitative PCR (qPCR) was used to validate gene expression in tumor and matched normal tissues. Finally, we integrated clinical data to construct a prognostic model.

**Results:**

The GSE31684 and GSE48276 cohorts were divided into high immunity (Immunity_H) and low immunity (Immunity_L) groups, and there were significant microenvironment differences between the Immunity_H and Immunity_L of the two cohorts, and there were many common differentially expressed genes (DEGs) between different immune subtypes of the two cohorts, which were mainly involved in immune-related biological processes. In addition, patients in the high-risk BLCA group exhibited significantly worse prognosis than those in the low-risk group. qPCR analysis confirmed that the expression levels of the risk-stratification genes were significantly different between BLCA tumors and matched adjacent normal tissues. The integrated analysis of tumor mutation burden (TMB) and our risk stratification revealed that patients with low-risk scores and high TMB exhibited the most favorable prognosis. Furthermore, the risk score was validated as an independent prognostic factor through both univariate and multivariate Cox regression analyses. Consequently, we constructed a nomogram that incorporates these findings to assist clinicians in prognostic assessment for BLCA patients.

**Conclusion:**

Given that the tumor microenvironment significantly influences BLCA prognosis, our finding that risk stratification serves as an independent prognostic indicator underscores the clinical relevance of our model. This stratification strategy has the potential to improve prognostic assessment and inform personalized treatment planning for BLCA patients.

## Introduction

In 2020, nearly 600000 new cases of bladder cancer were diagnosed globally (Sung et al., 2021). By 2040, this number is projected to double (Dyrskjøt et al., 2023). More than 90% of the pathological types of bladder cancer are bladder urothelial carcinoma (BLCA) (Zhang et al., 2021), so this paper mainly discusses and studies BLCA. The microenvironment of BLCA has strong heterogeneity (Hoadley et al., 2014; Warrick et al., 2019), effective management of BLCA requires a multidisciplinary approach that comprehensively considers patient characteristics and the molecular features of the disease.

Currently, the diagnosis and follow-up of BLCA rely on a comprehensive assessment using cystoscopy, histology, and cytology. However, the detection and screening of BLCA remain insufficient, as a significant number of patients are diagnosed at an advanced stage. While early-stage BLCA is more amenable to effective treatment compared to late-stage disease, it has a high recurrence rate (Zhao et al., 2023). Moreover, even among patients with BLCA of the same stage and grade undergoing identical treatment regimens, there can be substantial differences in recurrence times and prognoses, which may be related to the heterogeneity of tumor microenvironment (TME) in cancer patients (Prasetyanti and Medema, 2017; Burrell et al., 2013). Therefore, further risk stratification of BLCA is crucial for better prognosis assessment and timely adjustment of treatment strategies. Elucidating the molecular mechanism of BLCA is the basis of precise treatment of BLCA. Detection of genes and related gene expression products in cancer tissue plays a crucial role in BLCA molecular subtyping (Zhu et al., 2020).

TME is a highly heterogeneous biological system comprising immune cells, cancer cells, extracellular matrix components, and various signaling molecules (Warrick et al., 2019; Hu et al., 2021a; da Costa et al., 2018). The expression of immune cells and their related genes is a crucial component of the TME, significantly impacting the prognosis of BLCA (Zhang et al., 2022).

In this study, we performed immunotyping on BLCA patients from GSE31684 and GSE48276, comparing the tumor microenvironment and differentially expressed genes between the identified immune subtypes. Subsequently, we integrated TCGA and GEO transcriptomic datasets to establish a comprehensive risk stratification system for BLCA. Based on this analysis, we developed a predictive model to facilitate clinical prognosis evaluation, enabling timely therapeutic optimization for BLCA patients.

## Methods

### Acquisition and processing of GSE31684 and GSE48276 data from GEO database

Firstly, the gene expression matrices of 93 BLCA samples from GSE31684 and 116 BLCA samples from GSE48276 were obtained through the “Biobase” and “GEOquery” packages of R language (R 4.4.1). The raw gene expression matrices obtained from the GEO database were already preprocessed and normalized by the original submitters using the robust multi-array average (RMA) method. We directly utilized these normalized expression data for our subsequent ssGSEA and differential expression analyses. Both datasets exclusively contain profiles from Bladder Urothelial Carcinoma (BLCA) patients, which aligns perfectly with the focus of our study. Each dataset contains a substantial number of samples (GSE31684: n = 93; GSE48276: n = 116), which provides sufficient statistical power for reliable subgroup identification and differential expression analysis. According to the expression information of 29 immune-related gene sets in each sample, the immune-related characteristics of GSE31684 and GSE48276 samples were evaluated comprehensively, that is, gene set enrichment analysis of a single sample (ssGSEA) (Wu et al., 2022; Hanzelman et al., 2013). Based on the ssGSEA score, Euclidean distance and Ward’s linkage (Guan et al., 2020), the samples of GSE31684 and GSE48276 were divided into two groups, namely, the low immunity group (Immunity_L) and the high immunity group (Immunity_H). The Immunity_L of GSE31684 dataset includes 71 samples, and Immunity_H includes 22 samples. The Immunity_L of GSE48276 dataset includes 74 samples, and Immunity_H includes 42 samples. We also used t-distributed Stochastic Neighbor Embedding (tSNE) algorithm to cluster the Immunity_H and Immunity_L groups again. Then, the tumor microenvironment of the Immunity_L and Immunity_H groups of GSE31684 and GSE48276 datasets was evaluated by “limma” package in R language. We compared the expression levels of HLA-related genes between Immunity_H and Immunity_L groups, and also compared the levels of immune cell infiltration between Immunity_h and Immunity_L groups by CIBERSORT analysis (Newman et al., 2015). In addition, we focused on genes that were differentially expressed between Immunity_H and Immunity_L in the GSE31684 and GSE48276 datasets. With Immunity_L as the control group, the *P* value less than 0.05 and the absolute value of logFC greater than 0.585 as the standard to measure the differentially expressed genes (DEGs) between different immune subtypes. When integrating the gene expression data from the two GEO cohorts (GSE31684 and GSE48276) for the identification of common DEGs, we employed the ComBat algorithm from the “sva” R package to adjust for potential batch effects arising from different experimental batches or platforms. This step ensured that the identified common DEGs were more likely to be biologically relevant rather than technical artifacts.

### The acquisition and collation of BLCA data from TCGA

The clinical information and expression matrix of BLCA from TCGA were downloaded and sorted out with R language. The RNA-seq data (in FPKM format) downloaded from TCGA were log2-transformed (log2(FPKM+1)) to approximate a normal distribution before any downstream analysis. Normal tissue samples were removed, and 411 BLCA samples were obtained for subsequent analysis. The expression matrix of DEGs in the two GEO datasets (GSE31684 and GSE48276) from TCGA was obstained using the “sva” and “limma” packages. For the integration of GEO-derived DEGs with the TCGA dataset, we utilized the limma package’s removeBatchEffect function prior to survival analysis to minimize non-biological variance. At the same time, gene expression data and survival data of 403 samples with complete clinical information (including sex, age, grade, stage and survival information) of BLCA patients were combined through the “limma” package. The prognostic related genes (PRGs) of BLCA were identified by analyzing the combined gene expression matrix and survival data using the “survival” package. Download tumor-related transcription factors (TFs) from the website (http://www.cistrome.org/), and construct the co-expression analysis of PRGs and TFs by “dplyr” and “ggalluvial” packages to further explore the possible causes of PRGs affecting the prognosis of BLCA. Gene oncology (GO) analysis was used to explore the primary biological processes involving these PRGs.

### Risk stratification of BLCA

Based on the expression of PRGs and survival information of the samples, the lasso regression analysis was performed on 403 samples with complete clinical information, and 30 genes for risk score and their corresponding coefficients were obtained. The LASSO Cox regression was performed using the “glmnet” R package. To determine the optimal penalty parameter (lambda) and prevent overfitting, we employed 10-fold cross-validation. This process was repeated 100 times to enhance the stability and reliability of the lambda selection. The optimal lambda value was selected based on the minimum partial likelihood deviance criterion (i.e., lambda.min). The 403 samples were divided into high-risk (201 samples) and low-risk (202 samples) groups based on the median risk score. The genes and their coefficients used to calculate the risk score are shown in Supplementary Table S1. Moreover, we compared the survival outcomes of high and low risk groups with the “survival” package.

### UALCAN

UALCAN is a powerful website that can be used to analyze the associations between transcriptomic, proteomic, and patient survival information across various cancers (Chandrashekar et al., 2022). We used UALCAN to explore the impact of the three genes with the largest or smallest coefficients used to calculate risk scores on survival in BLCA patients.

### Tumor mutation burden

Tumor mutation burden (TMB) data of BLCA were downloaded from TCGA, and TMB of each sample was calculated through Strawberry Perl software. Using “limma” and “ggpubr” packages in R language to compare the TMB of high and low risk groups. Simultaneously, we evaluated the association between TMB and clinical outcomes in BLCA patients. Furthermore, we performed integrated analysis to assess the combined prognostic value of both risk stratification and TMB in patients.

### A prognostic model for BLCA

Given the significance of risk stratification based on gene expression, we further assessed its superiority in evaluating BLCA prognosis by univariate and multifactorial independent prognostic analyses. Last but not least, through the “timeROC” and “rms” packages of R language, we devised a comprehensive scoring system based on risk stratification, incorporating patient grade, stage, age, and gender to systematically evaluate the outcomes of BLCA.

### qPCR

We obtained tumor and adjacent normal tissue samples from four bladder cancer (BLCA) patients at the Affiliated Hospital of Guizhou Medical University. Using qPCR, we compared the expression levels of either the three genes with the highest coefficients or the three genes with the lowest coefficients in our risk score calculation model. Total RNA was extracted from tissue samples using TRIzol reagent. RNA purity and concentration were verified by spectrophotometry with acceptable A260/A280 ratios between 1.8–2.0. Total RNA was reverse transcribed into cDNA following the manufacturer’s protocol (Vazyme, R323-01). Post-amplification, the comparative 2^−ΔΔCT^ method was employed to quantify differential gene expression between malignant and matched paracancerous tissues. qPCR was performed using the primer sequences specified below.

GAPDH:

5′-AATCAAGTGGGGCGATGCTG-3' (Forward),

5′-GCAAATGAGCCCCAGCCTTC-3′(Reverse);

ADCY7 (Adenylate Cyclase 7):

5′-GATGTACGTCGAGTGTCTCCT-3' (Forward),

5′- CTTTGTCCATGCGTCGAACA-3' (Reverse);

SLC1A6 (Solute Carrier Family 1 Member 6):

5′-CTCAACCTGGGTCAGATCACA-3' (Forward),

5′-CCGACCGACGTAAGCACAA-3' (Reverse);

NELL2 (Neural EGFL Like 2):

5′-GAGCTGAACAGCGAATGAATAGA-3' (Forward),

5′-AATTCTCGGTAGGTGGTTCCC-3' (Reverse);

ZNF823 (Zinc Finger Protein 823):

5′-GTCGTCTTGGGTCATTCGTCT-3' (Forward),

5′-ATGTGTCTTCGGAGGTTTCCA-3' (Reverse);

ITGB7 (Integrin Subunit Beta 7):

5′-TGGACCTGAGCTACTCCATGA-3' (Forward),

5′-GGTGAAAGCTGAATGGTGACTG-3' (Reverse);

CTLA4 (Cytotoxic T-Lymphocyte Associated Protein 4):

5′-GCCCTGCACTCTCCTGTTTTT-3' (Forward),

5′-GGTTGCCGCACAGACTTCA-3' (Reverse).

## Results

### Immune subtypes of BLCA and their microenvironment comparison

**FIGURE 1:**
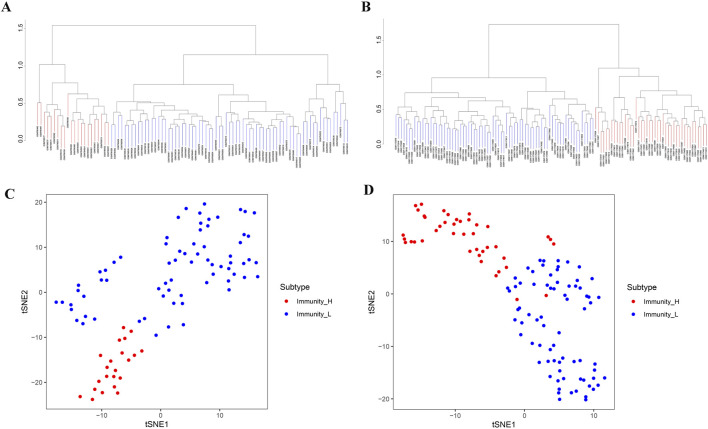
Two different immunophenotypes in BLCA patients. **(A,B)** Based on ssGSEA results, BLCA patients from GSE31684 **(A)** and GSE48276 **(B)** were stratified into Immunity_H and Immunity_L. **(C,D)** The reliability of BLCA immunophenotypes derived from GSE31684 **(C)** and GSE48276 **(D)** cohorts was verified by tSNE.

**FIGURE 2:**
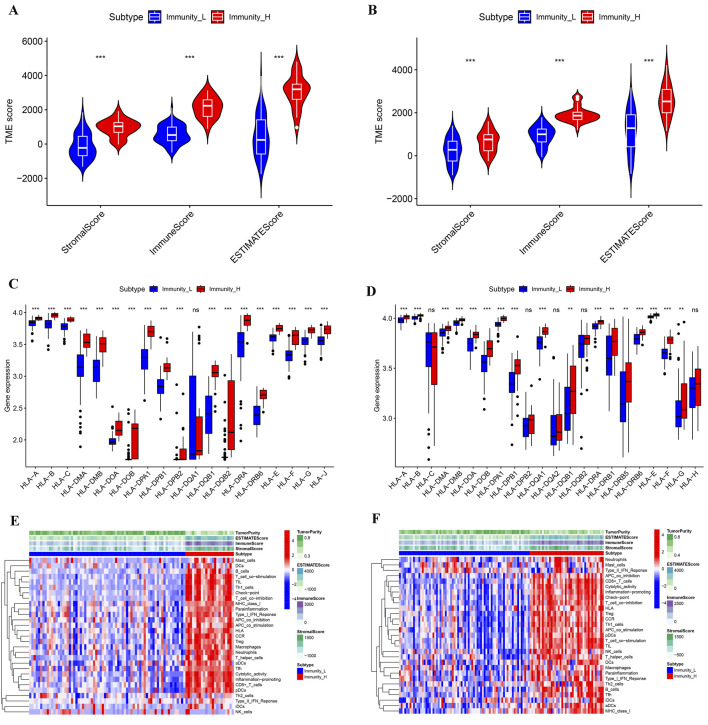
Multilevel comparison of the microenvironment among different immune subtypes of BLCA. **(A,B)** Comparison of StromalScore, ImmuneScore, and ESTIMATEScore for different immune subtypes from the GSE31684 **(A)** and GSE48276 **(B)** cohorts. **(C,D)** Comparison of HLA gene expression levels from different immune subtypes in GSE31684 **(C)** and GSE48276 **(D)** cohorts. **(E,F)** Microenvironmental landscapes of different immune subtypes in BLCA patients from the GSE31684 **(E)** and GSE48276 **(F)** cohorts. *p < 0.05; **p < 0.01; ***p < 0.001.

We performed ssGSEA analysis on 93 cancer samples from GSE31684 and 116 cancer samples from GSE48276, classifying them into two distinct immune subtypes: Immunity_H (high immune infiltration) and Immunity_L (low immune infiltration) (Figures 1A,B). Both t-SNE visualization and hierarchical clustering yielded consistent subtype classification patterns (Figures 1C,D), demonstrating that these computational approaches effectively discriminated between Immunity_H and Immunity_L subgroups. We compared the microenvironment of the Immunity_H and Immunity_L. As shown in Figure 2A, the tumor microenvironment scores were significantly elevated in the Immunity_H group compared to the Immunity_L group in the GSE31684 cohort (n = 93). Specifically, the StromalScore (*p* < 0.001), ImmuneScore (*p* < 0.001), and ESTIMATEScore (*p* < 0.001) were all markedly higher, as determined by the two-sided Student’s t-test. Similar results were observed in BLCA from GSE48276 (Figure 2B). Moreover, the expression of multiple HLA-related genes was significantly higher in Immunity_H group compared to the Immunity_H group (Figures 2C,D), and the immune-related functional scores were also significantly higher in Immunity_H (Figures 2E,F). These results indicate that there are significant differences in the immune microenvironment between the Immunity_H and Immunity_L, and the immune microenvironment is a crucial factor affecting cancer patient prognosis (Caramelo et al., 2023; Xie et al., 2024). Therefore, further exploration of BLCA based on immune subtyping is essential.

### Differential analysis of BLCA immune subtypes

**FIGURE 3:**
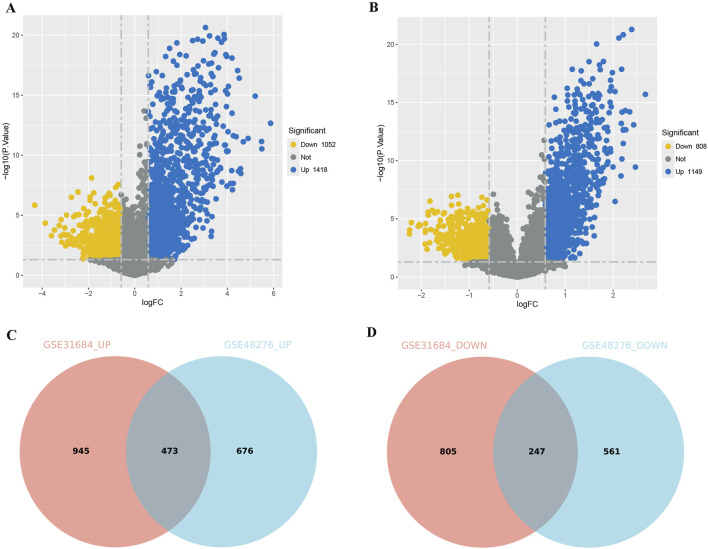
Comparison of DEGs among different immune subtypes of BLCA. **(A,B)** Volcano plots displaying the DEGs for different subtypes from the GSE31684 **(A)** and GSE48276 **(B)** cohorts. **(C,D)** A Venn diagram depicting the DEGs that are consistently upregulated **(C)** or downregulated **(D)** in the GSE31684 and GSE48276 cohorts (Immunity_L as the control group).

To further investigate the differences between immune subtypes, we compared the DEGs between the Immunity_H and Immunity_L, using the Immunity_L group as a control. In the BLCA of GSE31684, 1418 genes were upregulated and 1052 genes were downregulated in Immunity_H group (Figure 3A). In the BLCA of GSE48276, 1149 genes were upregulated and 808 genes were downregulated in Immunity_H group (Figure 3B). In the shared DEGs from GSE31684 and GSE48276, the Immunity_H group had 473 genes commonly upregulated and 247 genes commonly downregulated (Figures 3C,D).

### The combination of TCGA and GEO to explore the PRGs of BLCA

**FIGURE 4:**
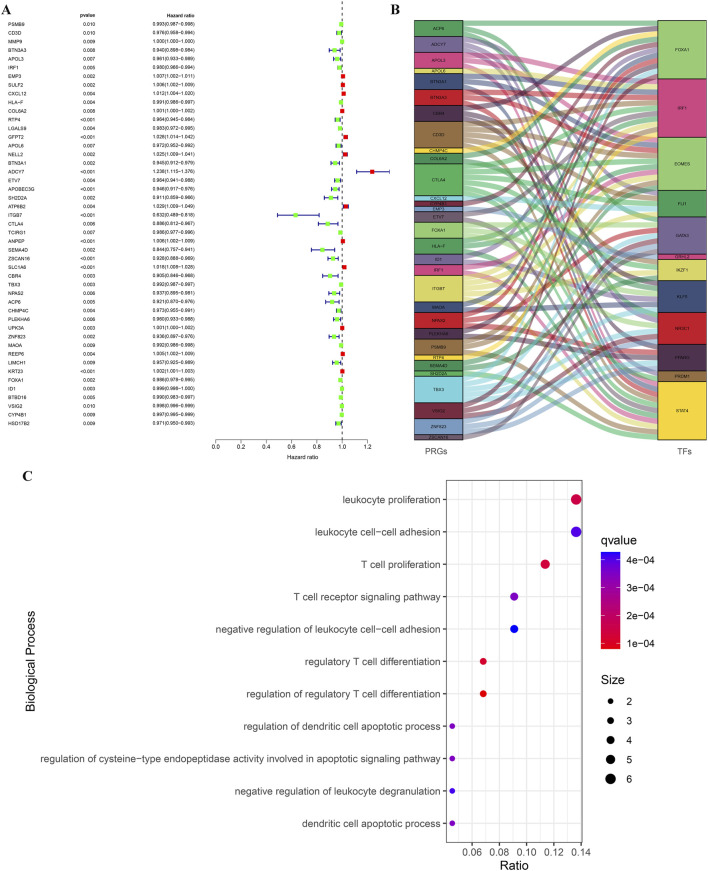
Identification of PRGs for BLCA based on TCGA and GEO data, along with the construction of their regulatory network. **(A)** The forest atlas showed common DEGs in the GSE31684 and GSE48276 cohorts that affected the survival outcomes of TCGA-BLCA patients. **(B)** A river interaction diagram showcasing the relationships between PRGs and TFs. **(C)** GO analysis revealed the main biological processes involved in PRGs.

Based on immune subtypes, we selected the shared DEGs (Immunity_H versus Immunity_L) in GSE31684 and GSE48276, then integrated TCGA survival data to explore prognostic genes in BLCA. We obtained a total of 47 genes that influence the prognosis of BLCA patients (Figure 4A). To gain a deeper understanding of BLCA development and prognostic differences, we explored the co-expression analysis of PRGs and tumor-related TFs. Multiple PRGs and TFs showed significant co-expression correlations (Figure 4B). Detailed co-expression information can be found in Supplementary Material S1. GO analysis showed that the biological processes involved in these PRGs were mainly immune related processes such as regulatory T cell differentiation, leukocyte proliferation, T cell receptor signaling pathway and leukocyte cell-cell adhesion (Figure 4C). These results highlight the complex regulatory network among PRGs and their strong association with immunity.

### Risk stratification of BLCA based on PRGs

**FIGURE 5:**
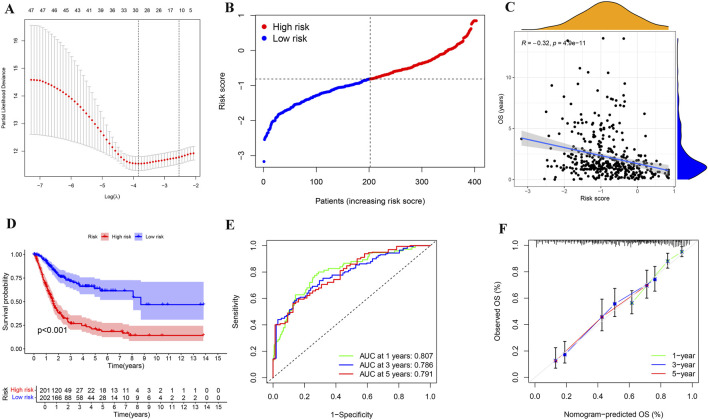
Determination and evaluation of risk assessment for patients with BLCA. **(A,B)** LASSO coefficient profiles **(A)** and cross-validation plot **(B)** for gene parameter selection within a lasso model of BLCA. **(C)** A distribution plot of risk scores for BLCA patients based on 30 genes identified through the lasso model. **(D)** Correlation analysis between OS and risk scores in patients with BLCA. **(E)** Kaplan-Meier survival analysis for BLCA across different risk groups. **(F)** The ROC analysis based on the lasso model yielded AUC values for 1-year, 3-year, and 5-year survival predictions of 0.807, 0.786, and 0.791, respectively. **(G)** A calibration plot for the risk characteristics of bladder cancer (BLCA) based on genes selected through the lasso model.

Given the importance of these PRGs in the development of BLCA, we performed lasso regression analysis based on PRGs and identified 30 genes for BLCA risk stratification (Figures 5A,B). Risk scores were then calculated from gene expression profiles and regression coefficients, enabling stratification of BLCA patients into high- and low-risk categories (Figure 5C). Kaplan-Meier survival analysis revealed a significantly poorer OS for patients in the high-risk group (n = 201) compared to those in the low-risk group (n = 202) (*p* < 0.001, Figure 5D). ROC analysis was used to determine the superiority of risk stratification in predicting survival of BLCA patients. The AUC values for 1-year, 3-year, and 5-year survival predictions from ROC analysis were 0.807, 0.786, and 0.791, respectively (Figure 5E). The calibration plot results showed that the predicted values based on risk stratification closely matched the actual values (Figure 5F), indicating the reliability of risk stratification for predicting BLCA prognosis.

**FIGURE 6:**
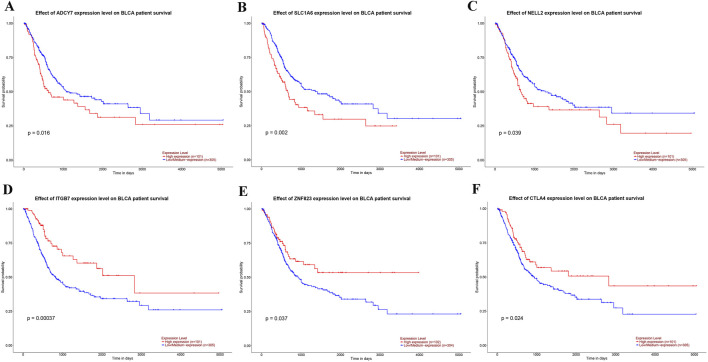
The impact of the expression levels of genes used for risk stratification on the prognosis of BLCA. **(A–C)** The influence of the expression levels of the three genes with the highest coefficients—ADCY7 **(A)**, SLC1A6 **(B)**, and NELL2 **(C)**—on the prognosis of BLCA. **(D–F)** The influence of the expression levels of the three genes with the smallest coefficients—ITGB7 **(D)**, ZNF823 **(E)**, and CTLA4 **(F)**—on the prognosis of BLCA.

We further explored the effect of the six genes with the largest (ADCY7, SLC1A6, NELL2) or smallest (ITGB7, ZNF823, CTLA4) risk score coefficients on BLCA prognosis. Surprisingly, we found that high expression of the top three genes with risk coefficients greater than 0 (ADCY7, SLC1A6, NELL2) was detrimental to the survival prognosis of BLCA patients (Figures 6A–C), while high expression of the three genes with the smallest risk coefficients less than 0 (ITGB7, ZNF823, CTLA4) was beneficial for their survival prognosis (Figures 6D–F). These results are consistent with our risk score findings, further demonstrating the reliability of risk stratification.

### The expression of PRGs in BLCA and adjacent normal tissues

**FIGURE 7:**
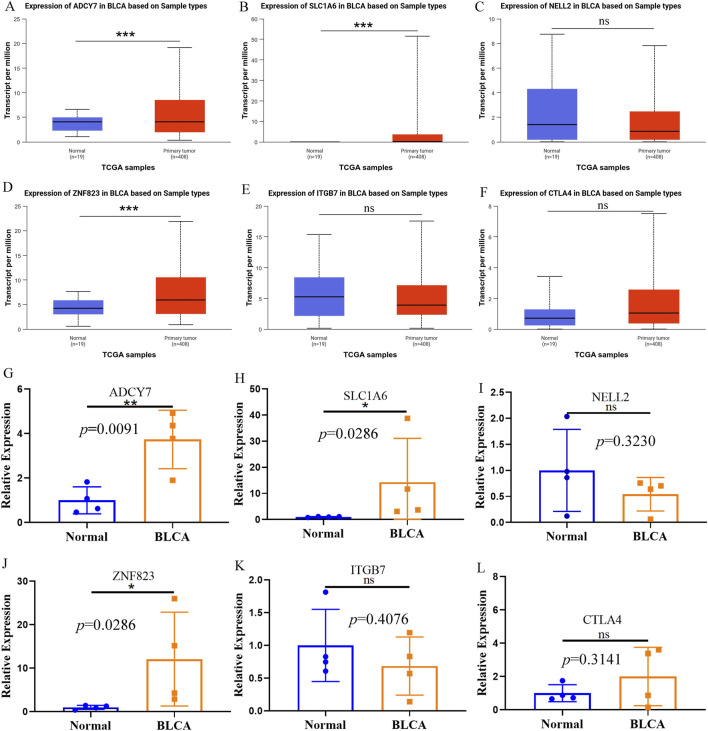
The expression of PRGs in BLCA and adjacent normal tissues. **(A–F)** The expression of ADCY7 **(A)**, SLC1A6 **(B)**, NELL2 **(C)**, ZNF823 **(D)**, ITGB7 **(E)** and CTLA4 **(F)** in BLCA and adjacent tissues in the UALCAN database. **(G–L)** The qPCR results confirmed the expression of ADCY7 **(G)**, SLC1A6 **(H)**, NELL2 **(I)**, ZNF823 **(J)**, ITGB7 **(K)** and CTLA4 **(L)** in BLCA and adjacent tissues. *p < 0.05; **p < 0.01; ***p < 0.001.

Further investigation using the UALCAN database revealed distinct expression patterns of ADCY7, SLC1A6, NELL2, ITGB7, ZNF823, and CTLA4 in BLCA compared to adjacent normal tissues. Among the three genes with highest risk-score coefficients, ADCY7 and SLC1A6 showed significantly elevated expression in BLCA (Figures 7A,B), while NELL2 exhibited no significant differential expression (Figure 7C). Conversely, among the three genes with lowest risk score coefficients, ZNF823 was markedly upregulated in tumor tissues (Figure 7D), whereas ITGB7 and CTLA4 demonstrated comparable expression levels between BLCA and normal tissues (Figures 7E,F). These database findings were subsequently validated by qPCR experiments (Figures 7G–L).

### Combining risk stratification with TMB to assess BLCA prognosis

**FIGURE 8:**
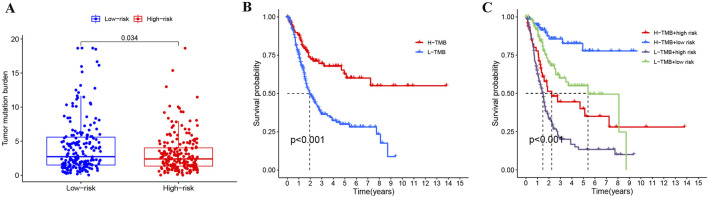
Joint analysis of risk stratification and TMB in BLCA. **(A)** Comparison of TMB across different risk groups in BLCA. **(B)** Impact of TMB on BLCA prognosis. **(C)** Evaluating the prognosis of BLCA by combining TMB and risk stratification.

Given that tumor mutational burden (TMB) significantly influences cancer prognosis (Provencio et al., 2023; Sung et al., 2022), we compared TMB levels between high- and low-risk BLCA patient groups. Analysis of tumor mutation burden (TMB) between the risk groups showed that the high-risk group (n = 201) had a significantly lower TMB than the low-risk group (n = 202) (*p* = 0.034, Figure 8A). Moreover, BLCA patients with high TMB (H-TMB) have a better survival prognosis than those with low TMB (L-TMB) (Figure 8B). Studies have reported that colorectal cancer patients with high TMB have a better prognosis (Wang et al., 2022), which is similar to our results. Integrating both TMB and risk stratification, we found BLCA patients with H-TMB and low-risk status showed optimal survival outcomes, whereas those with L-TMB and high-risk status had the poorest prognosis (Figure 8C).

### Independent prognostic analysis and predictive model construction for BLCA

**FIGURE 9:**
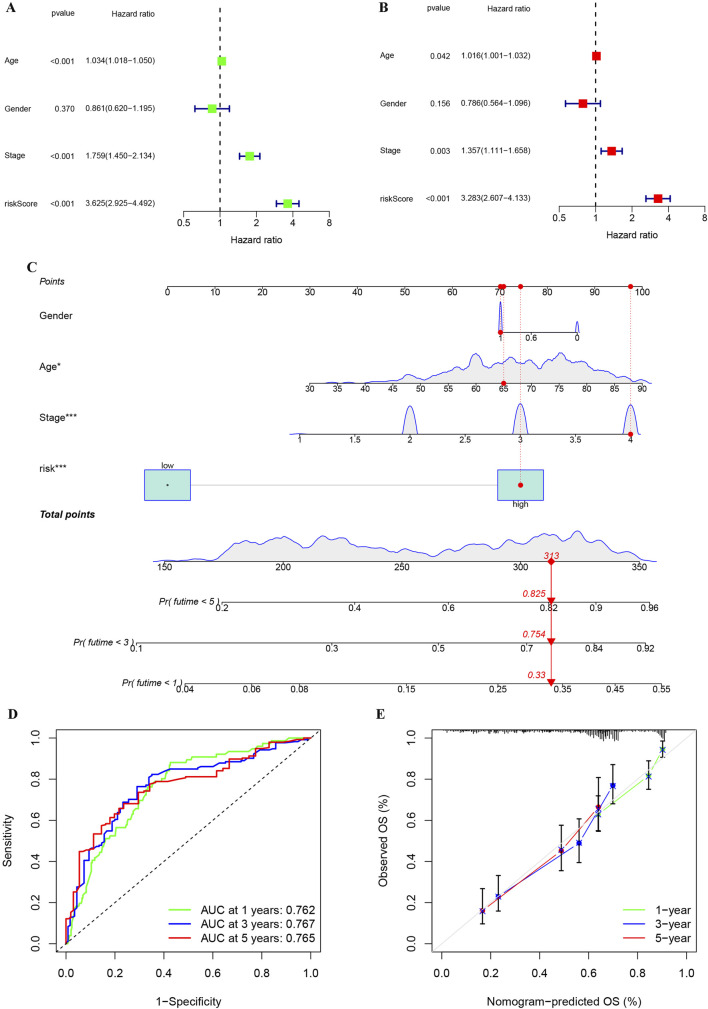
Univariate and multivariate analyses of the impact of risk stratification on BLCA, along with the establishment and evaluation of a nomogram model. **(A)** Analysis of risk stratification and other variables in BLCA using univariate cox regression. **(B)** Analysis of risk stratification and other variables in BLCA using multivariate cox regression. **(C)** Construction of a nomogram based on clinical features and risk stratification for BLCA.**(D)** Assessment of the reliability of the nomogram through ROC analysis. **(E)** Map of calibration used to compare the nomogram to the ideal model for similarity assessment. *p < 0.05; **p < 0.01; ***p < 0.001.

To evaluate the independent prognostic value of our risk score, we performed Cox regression analyses. Univariate Cox regression analysis identified the risk score as a significant prognostic factor (HR = 3.625, *p* < 0.001). Importantly, in the multivariate analysis adjusted for age, gender, grade, and stage, the risk score remained an independent predictor of overall survival (HR = 3.283, *p* < 0.001), confirming its prognostic value beyond standard clinical parameters (Figures 9A,B). To facilitate clinical prognosis evaluation and timely treatment adjustment, we developed a comprehensive prognostic model incorporating risk stratification, age, stage, and grade using BLCA patient data. The model generates a total score where higher values correlate with increased probability of survival below 1, 3, and 5 years (Figure 9C). ROC analysis confirmed the nomogram’s high predictive accuracy for BLCA patient survival (Figure 9D). At the same time, calibration plot also indicated that the performance of the nomogram was highly similar to that of the ideal model (Figure 9E). These results demonstrate the reliability and practicality of our comprehensive assessment, incorporating clinical information and risk stratification, in predicting the prognosis of BLCA patients.

## Discussion

Due to the unique nature of its surgery, BLCA significantly impacts patients’ quality of life (Tang et al., 2020). To better adjust treatment plans and effectively assess the prognosis of BLCA, further exploration of its molecular subtypes is necessary. Studies have shown that the heterogeneity of the TME is a significant factor affecting cancer prognosis and the efficacy of drug treatments (Schulz et al., 2019; Hanahan and Weinberg, 2011), and the expression of immune cells and immune-related genes is a critical component of the TME (Zhang et al., 2022). In this study, by mining the BLCA data of TCGA and GEO, we not only performed immune typing of BLCA according to immune-related genes, but also performed risk stratification of BLCA in combination with PRGs. Additionally, we developed a prognostic model to assist clinicians in evaluating BLCA patient outcomes and guiding timely treatment adjustments.

First of all, different cohorts of BLCA patients in GEO were divided into two immune subtypes, and it was found that these two immune subtypes had great differences in HLA-related gene expression and immune cell infiltration. These results indicate a large heterogeneity in the TME of BLCA. Previous studies have reported that differences in immune cell infiltration levels are a key factor contributing to variations in BLCA prognosis (Hu et al., 2021b; Xu et al., 2022; Debatin et al., 2024; Li et al., 2024). Therefore, we further explored how to effectively assess BLCA prognosis based on different immune subtypes, enabling clinicians to adjust treatment plans promptly and effectively. In order to ensure that our research is more reliable, we continued to conduct in-depth analysis of immunophenotyping in conjunction with TCGA. We identified multiple genes that influence the prognostic risk of BLCA, with a strong interaction observed between these prognostic genes and tumor transcription factors. This discovery lays the foundation for future exploration of the molecular mechanisms influencing BLCA prognosis. ADCY7 catalyzes the production of cyclic AMP (cAMP), a critical second messenger. In immune cells, high cAMP levels are a potent negative regulator of T cell activation and effector functions (Bayerl et al., 2023). This aligns with studies showing that cAMP-elevating pathways are a mechanism of immune evasion in cancers (Zha et al., 2022). Our finding positions ADCY7 as a potential mediator of immunosuppression in BLCA. SLC1A6 is a glutamate and aspartate transporter. Beyond its role in the nervous system, glutamate signaling is implicated in cancer (Ren et al., 2025). It can influence tumor cell proliferation, invasion, and calcium signaling (Bertero et al., 2019). The high expression of SLC1A6 in BLC and its association with poor prognosis suggest it may fuel aggressive tumor behavior. Furthermore, glutamate can modulate T cell function, and dysregulated glutamate metabolism in the TME is emerging as a contributor to cancer progression (Stanulovic et al., 2024). Thus, SLC1A6 may represent a novel metabolic driver in BLCA. ITGB7 pairs with α4 integrin to form α4β7 integrin, which is crucial for lymphocyte homing to mucosal tissues, including the gut and possibly the bladder mucosa (Nie et al., 2022). In our model, high ITGB7 expression is protective. This strongly suggests that a robust lymphocyte recruitment mechanism to the tumor site is a favorable prognostic factor. The presence of ITGB7 may indicate a more effective anti-tumor immune infiltration. This is consistent with the fundamental role of T cell recruitment in cancer immunotherapy (Zhu et al., 2025). CTLA-4 is a well-established immune checkpoint molecule on T cells. It transmits an inhibitory signal that serves as a critical “brake” on the immune response to prevent autoimmunity. It is also a premier target for cancer immunotherapy. In some studies, high intratumoral CTLA4 transcript levels can predict response to anti-CTLA-4 therapy and are associated with improved survival (Blanchard et al., 2025). This finding underscores the complexity TME in BLCA. Moreover, the biological processes involving these prognostic genes were closely related to immunity, further highlighting the significant impact of immunity on BLCA prognosis. It has been reported that the apoptosis and proliferation of immune cells play a significant role in BLCA prognosis (Liu Q. et al., 2024; Gao et al., 2024). Our study aligns with previous research and further explores the potential mechanisms influencing BLCA prognosis through tumor transcription factors and immunobiological processes.

In addition, the Immunity_H subtype exhibits an “activated-but-suppressed” microenvironment, characterized by concurrent enrichment of cytotoxic CD8^+^ T cells and immunosuppressive elements (M2 macrophages and some immune checkpoints). This indicates that the Immunity_H subtype needs to reach a state of immune balance in order to maximize its benefits for the patient’s survival. In contrast, the Immunity_L subtype represents an “immune-desert” phenotype, with minimal immune infiltration and a failure to initiate anti-tumor immunity, resulting in unfavorable outcomes.

Furthermore, we refined the prognostic gene signature through LASSO regression analysis, enabling stratification of BLCA patients into distinct high- and low-risk groups based on gene expression patterns. Integration with tumor mutational burden (TMB) further enhanced prognostic discrimination, revealing significant survival differences among BLCA subgroups. Consistent with established findings that TMB significantly influences prognosis through immune modulation (Chan et al., 2019; Jiang et al., 2022; Palmeri et al., 2021), and BLCA has a better survival prognosis in the low-risk + H-TMB group (Zhang et al., 2022). Our study also found similar results, indicating the reliability of our findings. TMB serves as a measure of tumor immunogenicity, where a higher load of mutations generates more neoantigens, potentially initiating a T-cell response (Westcott et al., 2023). Our risk model assesses the functional state of the tumor microenvironment (TME). A low-risk score indicates a TME permissive for immune cell function, while a high-risk score signifies an immunosuppressive TME. Therefore, the most favorable prognosis is observed in patients with High-TMB + Low-Risk scores. In this group, the “spark” of immunogenicity (neoantigens from high TMB) meets the “fertile ground” of a functional TME, enabling an effective anti-tumor immune response. Conversely, a high-risk TME can suppress the immune response even in the presence of high immunogenicity (High-TMB + High-Risk), leading to poorer outcomes. This framework explains the enhanced prognostic accuracy of the combined model. Moreover, our TMB + Risk model can stratify BLCA patients into distinct subgroups with direct therapeutic implications. For example, in Low-Risk + High-TMB group, these “ideal responders” possess both high immunogenicity and a functional TME, making them the strongest candidates for immune checkpoint blockade (ICB) therapy. In High-Risk + High-TMB, this group has the antigenic targets for immunotherapy but within a suppressive TME. They may require combinatorial strategies (ICB combined with TME-modulating agents) to overcome resistance. In High-Risk + Low-TMB, these “double-negative” patients, with low immunogenicity and a hostile TME, may derive less benefit from initial immunotherapy and could be prioritized for conventional chemotherapy or novel agents. This framework provides a actionable blueprint for personalizing treatment decisions in BLCA. However, the underlying molecular mechanisms through which TMB influences prognosis remain to be further elucidated in subsequent studies. We evaluated the impact of certain genes used for risk stratification on BLCA prognosis. Some genes were beneficial to BLCA prognosis when highly expressed, while others were advantageous when expressed at low levels, indicating that the genes selected for risk stratification are comprehensive and reasonable. Moreover, our qPCR results further validated the heterogeneous expression patterns of these prognostic genes between BLCA and matched adjacent tissues, further demonstrating the necessity of comprehensively judging the prognosis of BLCA based on these genes.

Current prognostic approaches for BLCA lack precision, highlighting the urgent need for robust stratification tools to guide clinical decision-making. We constructed a nomogram by combining risk stratification identified from multiple databases with patients’ clinicopathological information, providing clinicians with a tool to assess BLCA prognosis. Although nomograms for BLCA prognosis have been developed in previous studies (Zhang et al., 2022; Wu et al., 2022; Huang et al., 2023; Rodriguez-Enriquez et al., 2020; Liu L. et al., 2024), our nomogram offers more accurate predictive probabilities and covers a wider scoring range, demonstrating its greater reliability and applicability.

In summary, our study established a novel immune classification system for BLCA and developed a robust gene expression-based risk stratification model, effectively distinguishing high-risk and low-risk patient subgroups with distinct prognostic outcomes. We also integrated risk stratification and clinicopathological information to construct a nomogram that assists clinicians in assessing BLCA prognosis. However, it is undeniable that our study has certain limitations. On one hand, the qPCR validation of gene expression differences was performed on a limited number of patient samples (n = 4). While the results were consistent with the trends observed in the TCGA and UALCAN databases, this small sample size precludes strong statistical conclusions and necessitates validation in a larger, independent cohort. On the other hand, future studies should employ large-scale prospective cohort research to continuously refine and improve our predictive models, while further validating the biological functions of relevant target genes and proteins through both *in vivo* and *in vitro* experiments.

## Data Availability

The original contributions presented in the study are included in the article/Supplementary Material, further inquiries can be directed to the corresponding authors.
