# Supplementary material for: Progressively exploring and assessing the prognosis of bladder urothelial cancer based on the microenvironment through the integration of multiple databases
Source: Front Mol Biosci. 2025 Nov 19;12:1702311. doi: 10.3389/fmolb.2025.1702311 (PMC12672317; doi:10.3389/fmolb.2025.1702311)
Supplement: Supplementary file 1 [file DataSheet1.zip › all raw data/Ethics approval.pdf]

伦理证明

贵州医科大学附属医院泌尿外科邹雄撰写的文章《**Progressively exploring and assessing the prognosis of bladder urothelial cancer based on the microenvironment through the integration of multiple databases**》经医学伦理委员会审核，认为改论文内容不涉及患者隐私及商业利益，符合伦理相关法律法规要求，同意投稿。

贵州医科大学附属医院医学伦理委员会

2025.04.21
