# Supplementary material for: Progressively exploring and assessing the prognosis of bladder urothelial cancer based on the microenvironment through the integration of multiple databases
Source: Front Mol Biosci. 2025 Nov 19;12:1702311. doi: 10.3389/fmolb.2025.1702311 (PMC12672317; doi:10.3389/fmolb.2025.1702311)

	pvalue	Hazard ratio
PSMB9	0.010	0.993(0.987−0.998)
CD3D	0.010	0.976(0.958−0.994)
MMP9	0.009	1.000(1.000−1.000)
BTN3A3	0.008	0.940(0.898−0.984)
APOL3	0.007	0.961(0.933−0.989)
IRF1	0.005	0.980(0.966−0.994)
EMP3	0.002	1.007(1.002−1.011)
SULF2	0.002	1.006(1.002−1.009)
CXCL12	0.004	1.012(1.004−1.020)
HLA−F	0.004	0.991(0.986−0.997)
COL6A2	0.008	1.001(1.000−1.002)
RTP4	<0.001	0.964(0.945−0.984)
LGALS9	0.004	0.983(0.972−0.995)
GFPT2	<0.001	1.028(1.014−1.042)
APOL6	0.007	0.972(0.952−0.992)
NELL2	0.002	1.025(1.009−1.041)
BTN3A1	0.002	0.945(0.912−0.979)
ADCY7	<0.001	1.238(1.115−1.376)
ETV7	0.004	0.964(0.941−0.988)
APOBEC3G	<0.001	0.946(0.917−0.976)
SH2D2A	0.002	0.911(0.859−0.966)
ATP8B2	0.004	1.029(1.009−1.049)
ITGB7	<0.001	0.632(0.489−0.818)
CTLA4	0.006	0.886(0.812−0.967)
TCIRG1	0.007	0.986(0.977−0.996)
ANPEP	<0.001	1.006(1.002−1.009)
SEMA4D	0.002	0.844(0.757−0.941)
ZSCAN16	<0.001	0.928(0.888−0.969)
SLC1A6	<0.001	1.018(1.008−1.028)
CBR4	0.003	0.905(0.846−0.968)
TBX3	0.003	0.992(0.987−0.997)
NPAS2	0.006	0.937(0.895−0.981)
ACP6	0.005	0.921(0.870−0.976)
CHMP4C	0.004	0.973(0.955−0.991)
PLEKHA6	0.006	0.960(0.933−0.988)
UPK3A	0.003	1.001(1.000−1.002)
ZNF823	0.002	0.936(0.897−0.976)
MAOA	0.009	0.992(0.986−0.998)
REEP6	0.004	1.005(1.002−1.009)
LIMCH1	0.009	0.957(0.925−0.989)
KRT23	<0.001	1.002(1.001−1.003)
FOXA1	0.002	0.986(0.978−0.995)
ID1	0.003	0.999(0.998−1.000)
BTBD16	0.005	0.990(0.983−0.997)
VSIG2	0.010	0.998(0.996−0.999)
CYP4B1	0.009	0.997(0.995−0.999)
HSD17B2	0.009	0.971(0.950−0.993)

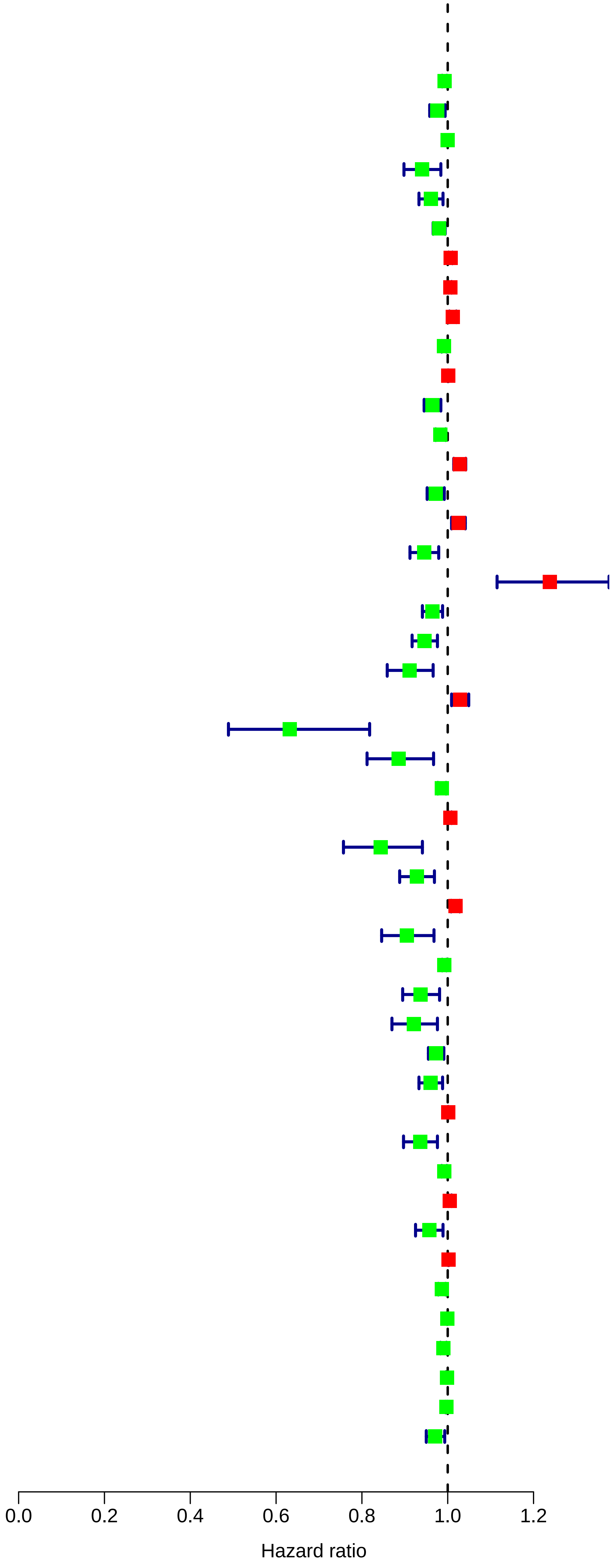

Supplement: Supplementary file 1 [file DataSheet1.zip › all raw data/Figures/Figure 4/Figure 4A.pdf]
