# Supplementary material for: Progressively exploring and assessing the prognosis of bladder urothelial cancer based on the microenvironment through the integration of multiple databases
Source: Front Mol Biosci. 2025 Nov 19;12:1702311. doi: 10.3389/fmolb.2025.1702311 (PMC12672317; doi:10.3389/fmolb.2025.1702311)
Supplement: Supplementary file 1 [file DataSheet1.zip › all raw data/Table S1.docx]

**Table S1：**

The genes and coefficients used to calculate the risk score for each sample

| Genes | Coefficients |
| --- | --- |
| ADCY7 | 0.173837 |
| SLC1A6 | 0.011979 |
| NELL2 | 0.011799 |
| ATP8B2 | 0.009539 |
| GFPT2 | 0.007682 |
| REEP6 | 0.003043 |
| EMP3 | 0.001724 |
| SULF2 | 0.001455 |
| KRT23 | 0.001291 |
| ANPEP | 0.001023 |
| UPK3A | 0.00069 |
| CXCL12 | 0.000288 |
| MMP9 | 0.000272 |
| MAOA | -3.08E-05 |
| ID1 | -0.00011 |
| CBR4 | -0.00057 |
| HLA-F | -0.00145 |
| APOL6 | -0.00443 |
| TCIRG1 | -0.0045 |
| CHMP4C | -0.00586 |
| LIMCH1 | -0.00848 |
| HSD17B2 | -0.01316 |
| RTP4 | -0.01434 |
| ZSCAN16 | -0.01583 |
| APOBEC3G | -0.01905 |
| SEMA4D | -0.02014 |
| SH2D2A | -0.02375 |
| CTLA4 | -0.02807 |
| ZNF823 | -0.0312 |
| ITGB7 | -0.2594 |
