# Supplementary figures and images for: Progressively exploring and assessing the prognosis of bladder urothelial cancer based on the microenvironment through the integration of multiple databases
Source: Front Mol Biosci. 2025 Nov 19;12:1702311. doi: 10.3389/fmolb.2025.1702311 (PMC12672317; doi:10.3389/fmolb.2025.1702311)

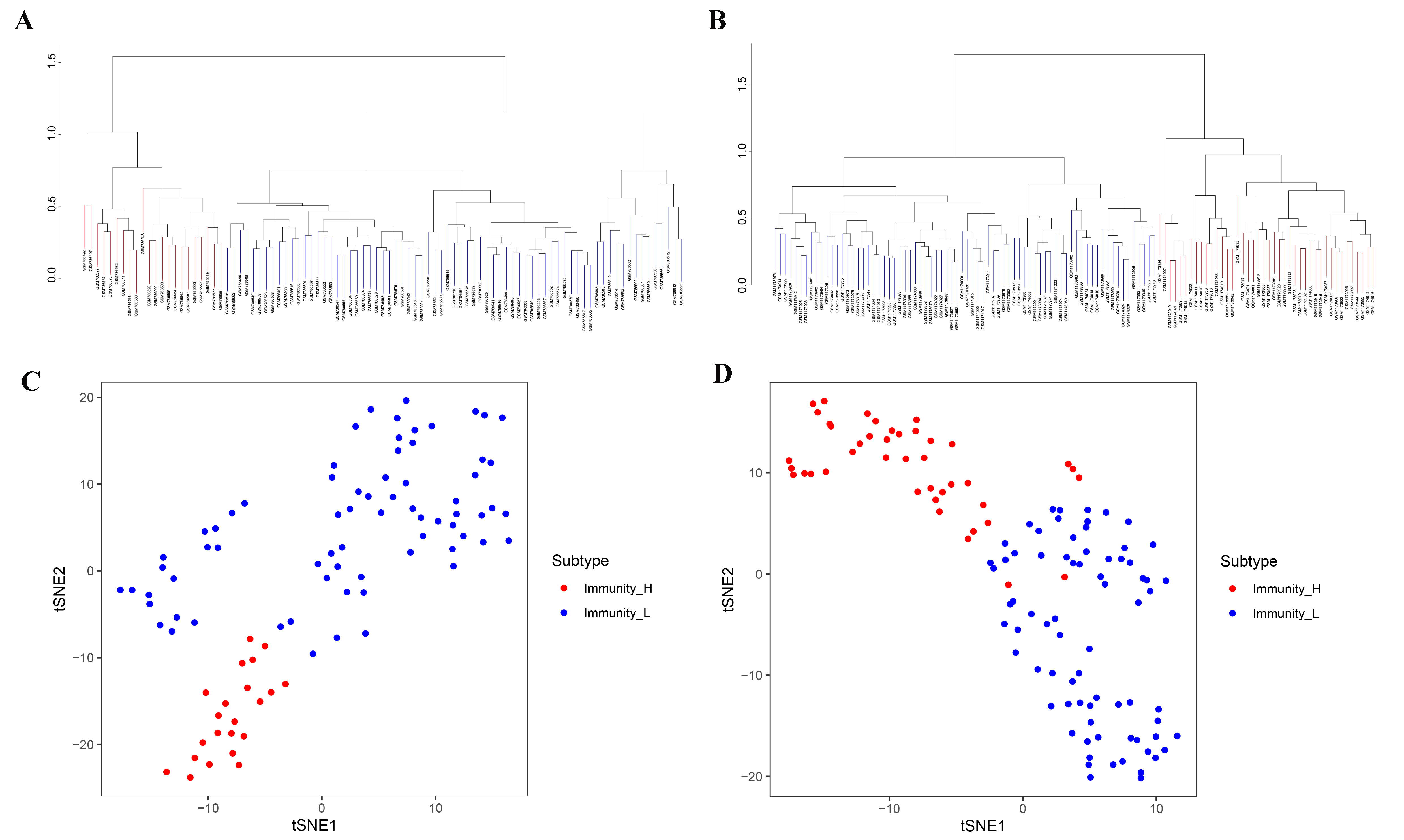

Supplement: Supplementary file 1 [file DataSheet1.zip › all raw data/Figures/Figure 1/Figure 1.jpg]

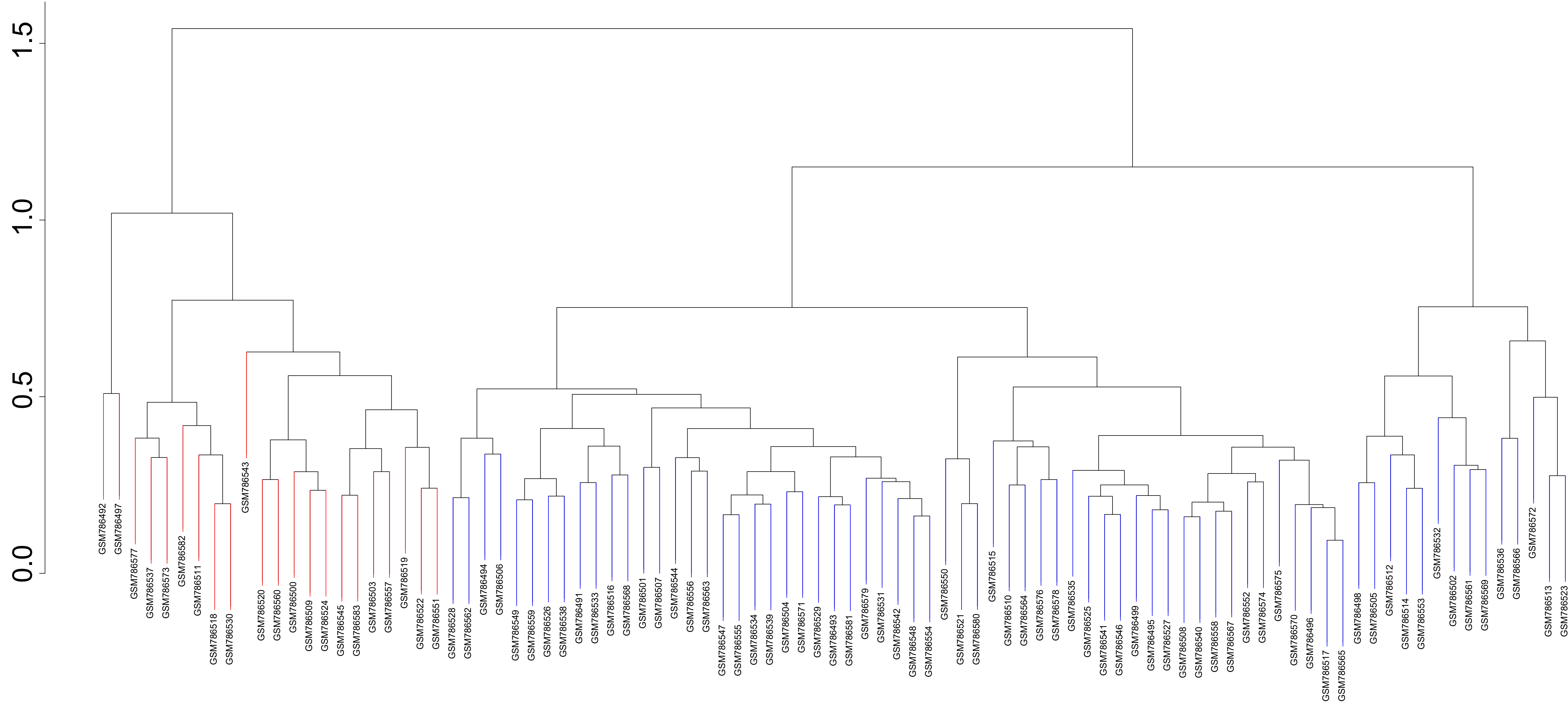

Supplement: Supplementary file 1 [file DataSheet1.zip › all raw data/Figures/Figure 1/Figure 1A.pdf]

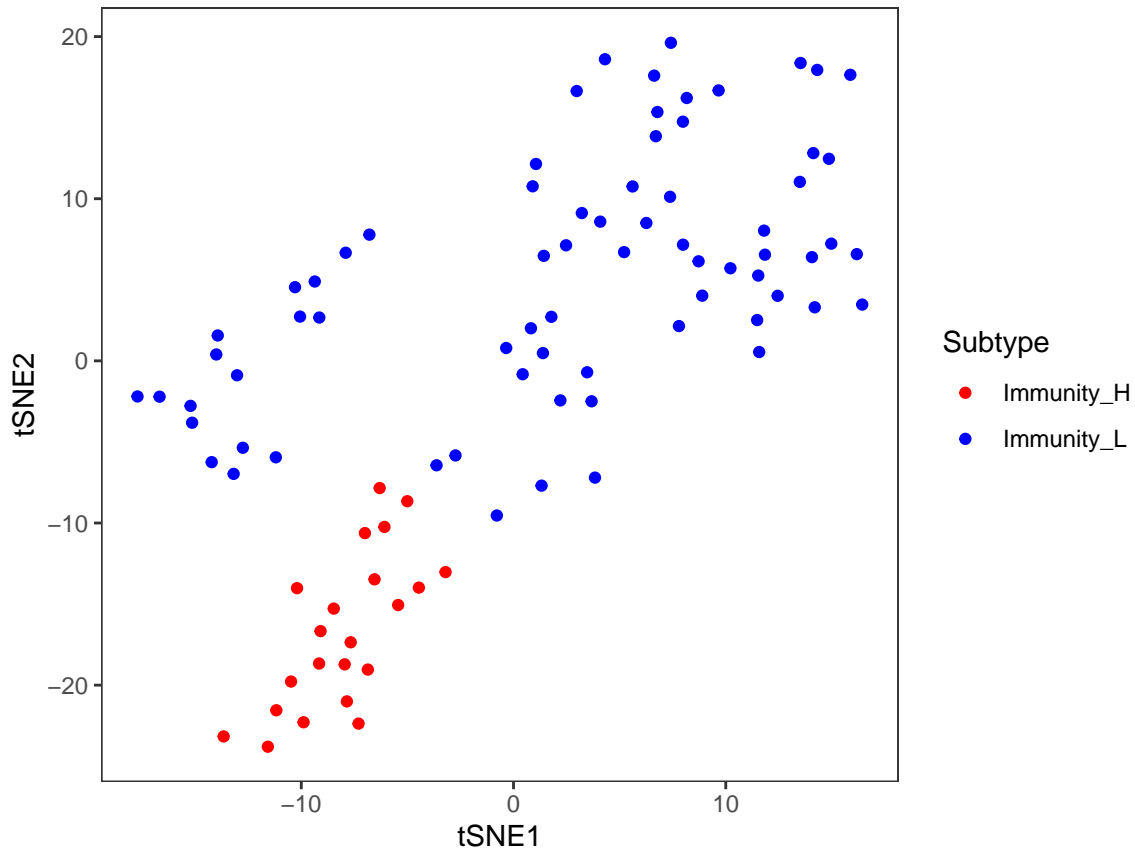

Supplement: Supplementary file 1 [file DataSheet1.zip › all raw data/Figures/Figure 1/Figure 1C.pdf]

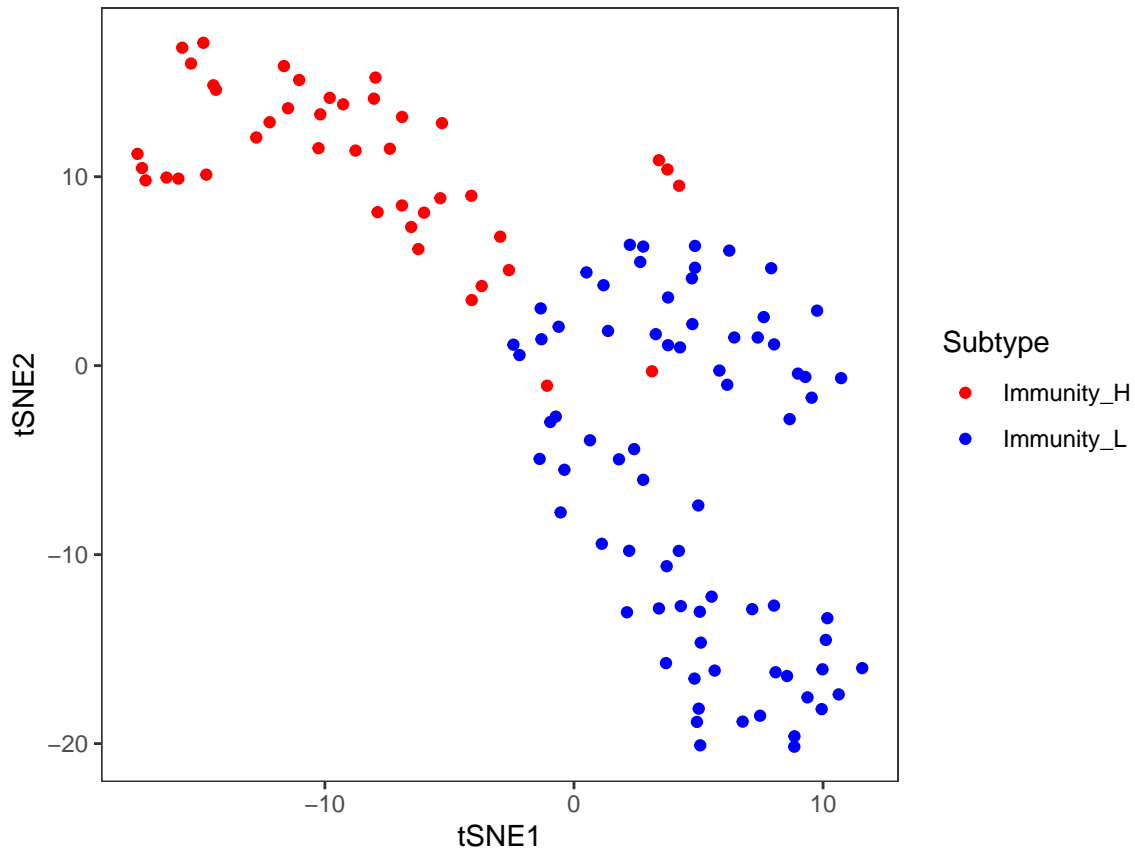

Supplement: Supplementary file 1 [file DataSheet1.zip › all raw data/Figures/Figure 1/Figure 1D.pdf]

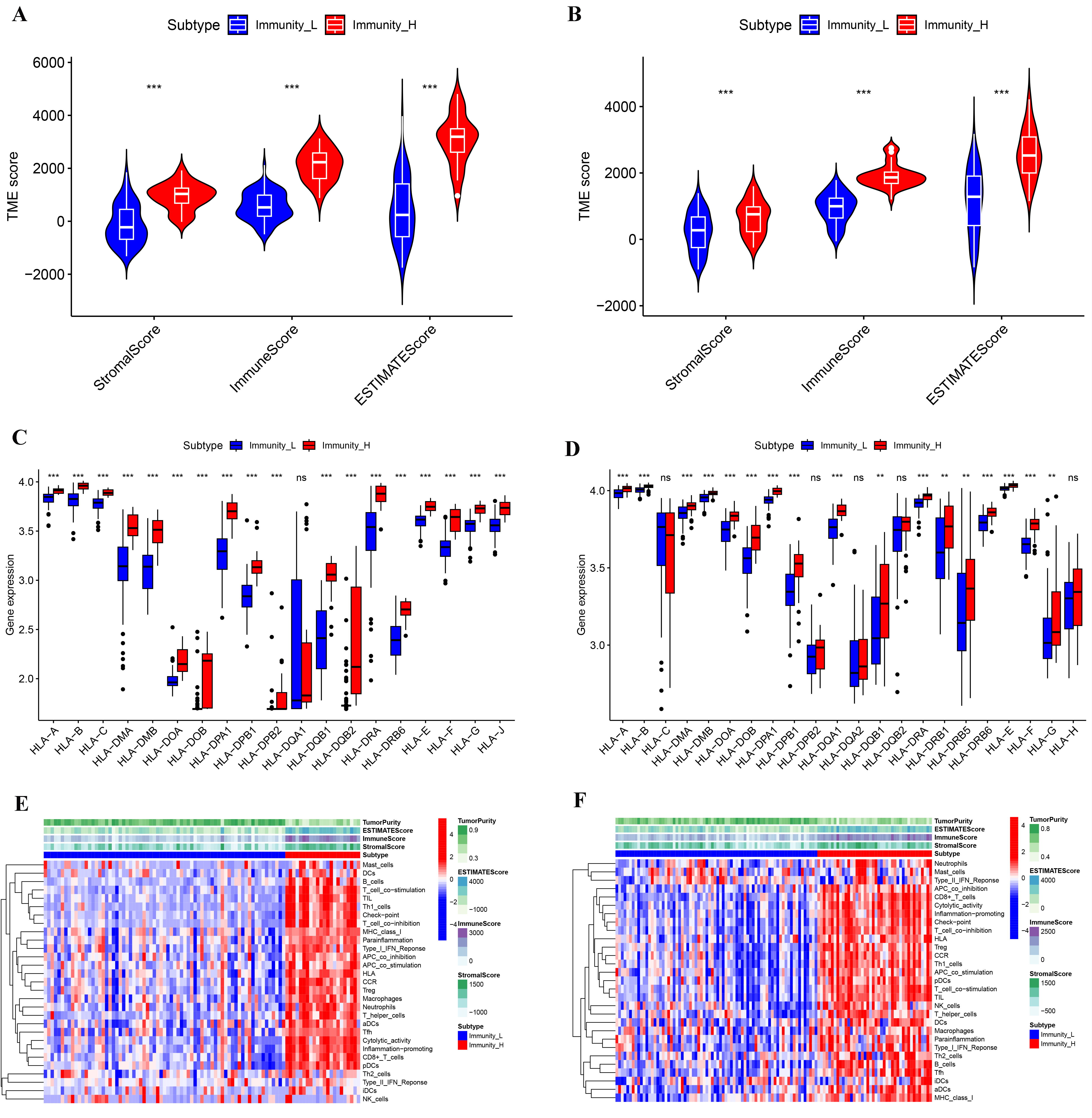

Supplement: Supplementary file 1 [file DataSheet1.zip › all raw data/Figures/Figure 2/Figure 2.jpg]

Subtype

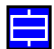

Immunity\_L

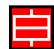

Immunity\_H

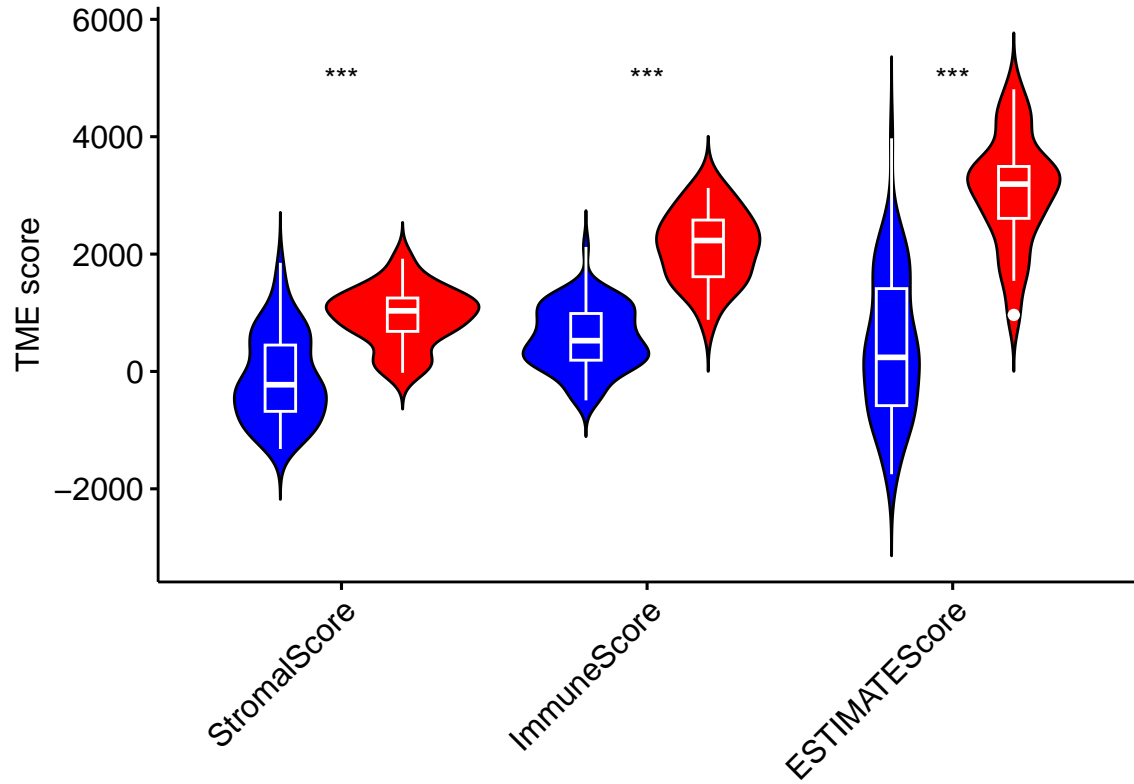

Supplement: Supplementary file 1 [file DataSheet1.zip › all raw data/Figures/Figure 2/Figure 2A.pdf]

Subtype

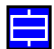

Immunity\_L

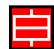

Immunity\_H

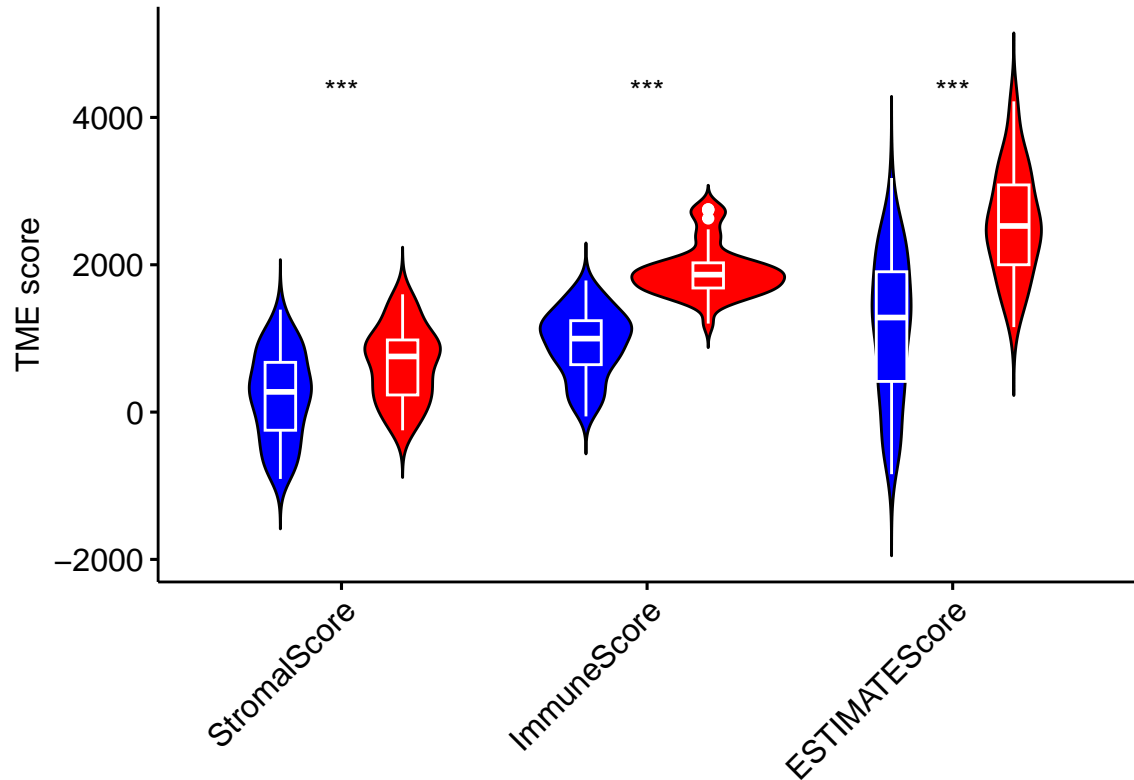

Supplement: Supplementary file 1 [file DataSheet1.zip › all raw data/Figures/Figure 2/Figure 2B.pdf]

Subtype Immunity\_L Immunity\_H

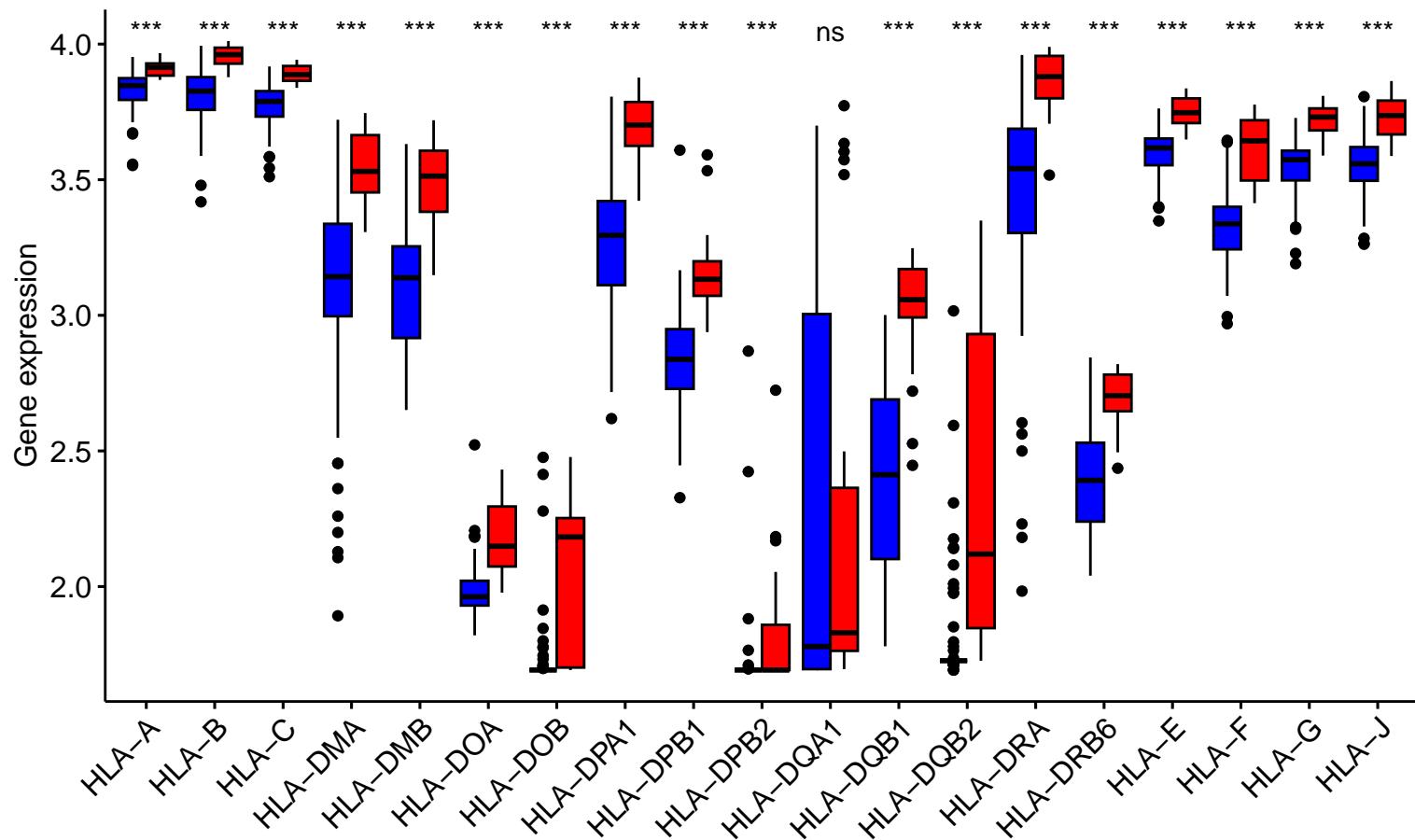

Supplement: Supplementary file 1 [file DataSheet1.zip › all raw data/Figures/Figure 2/Figure 2C.pdf]

Subtype Immunity\_L Immunity\_H

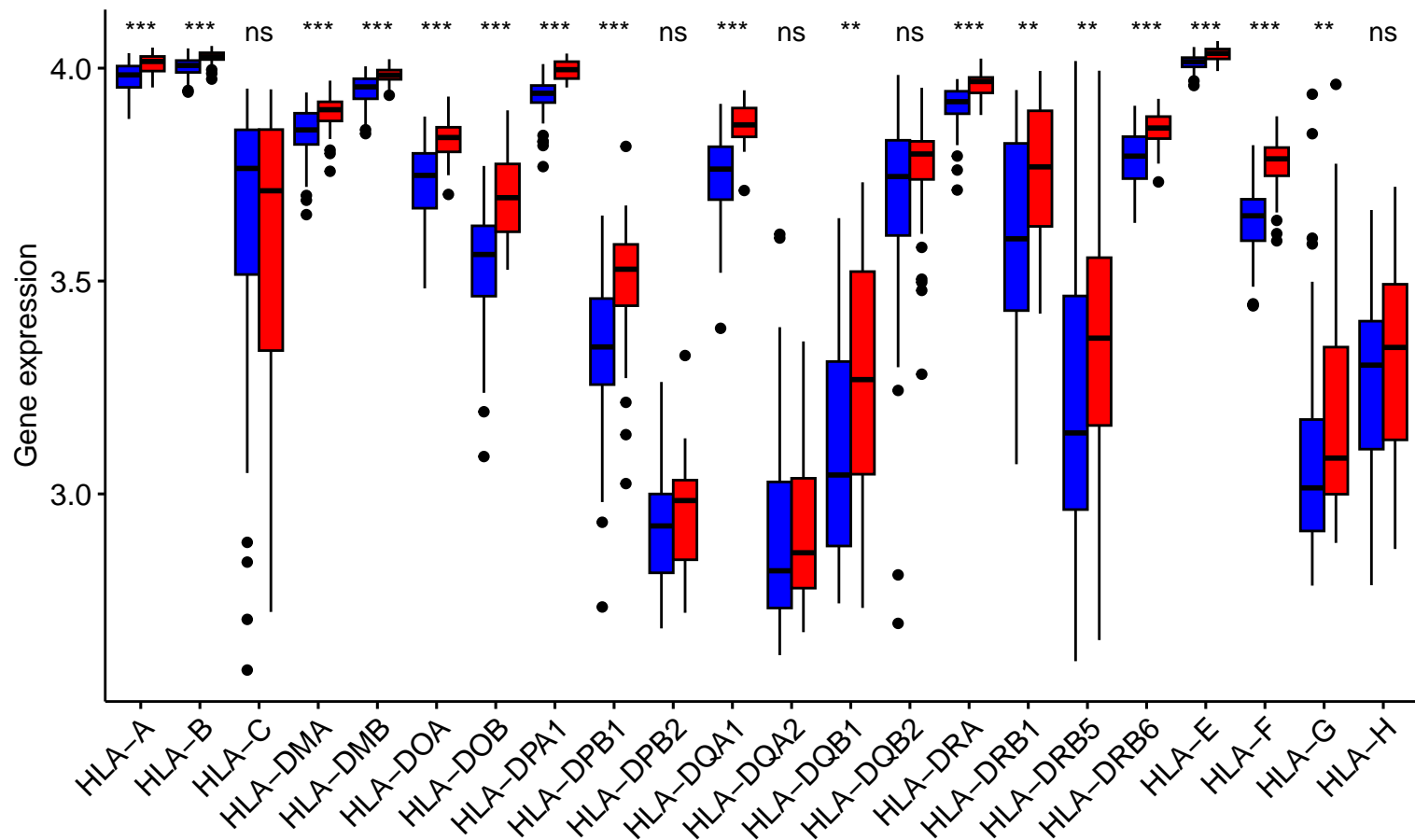

Supplement: Supplementary file 1 [file DataSheet1.zip › all raw data/Figures/Figure 2/Figure 2D.pdf]

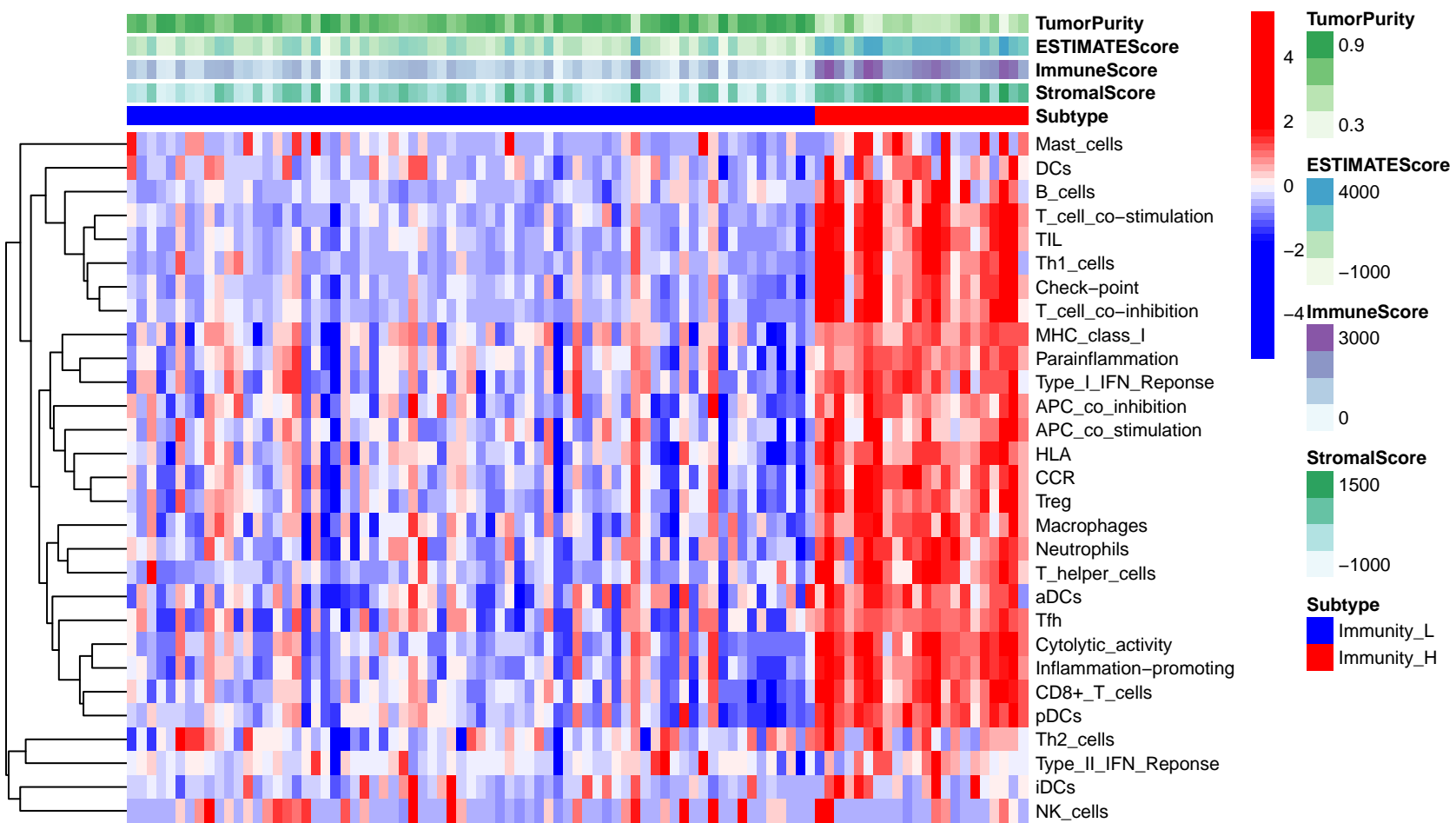

Supplement: Supplementary file 1 [file DataSheet1.zip › all raw data/Figures/Figure 2/Figure 2E.pdf]

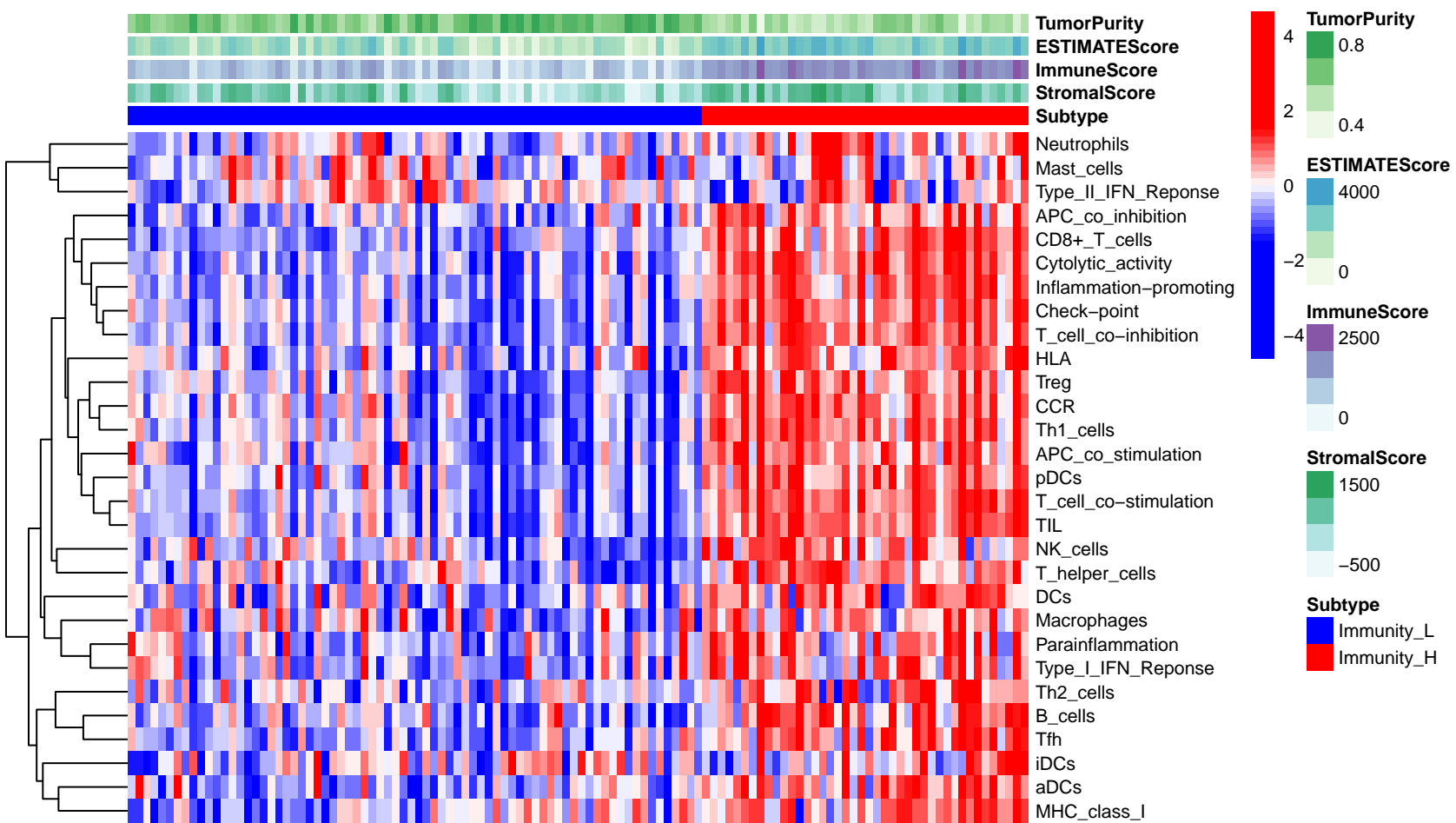

Supplement: Supplementary file 1 [file DataSheet1.zip › all raw data/Figures/Figure 2/Figure 2F.pdf]

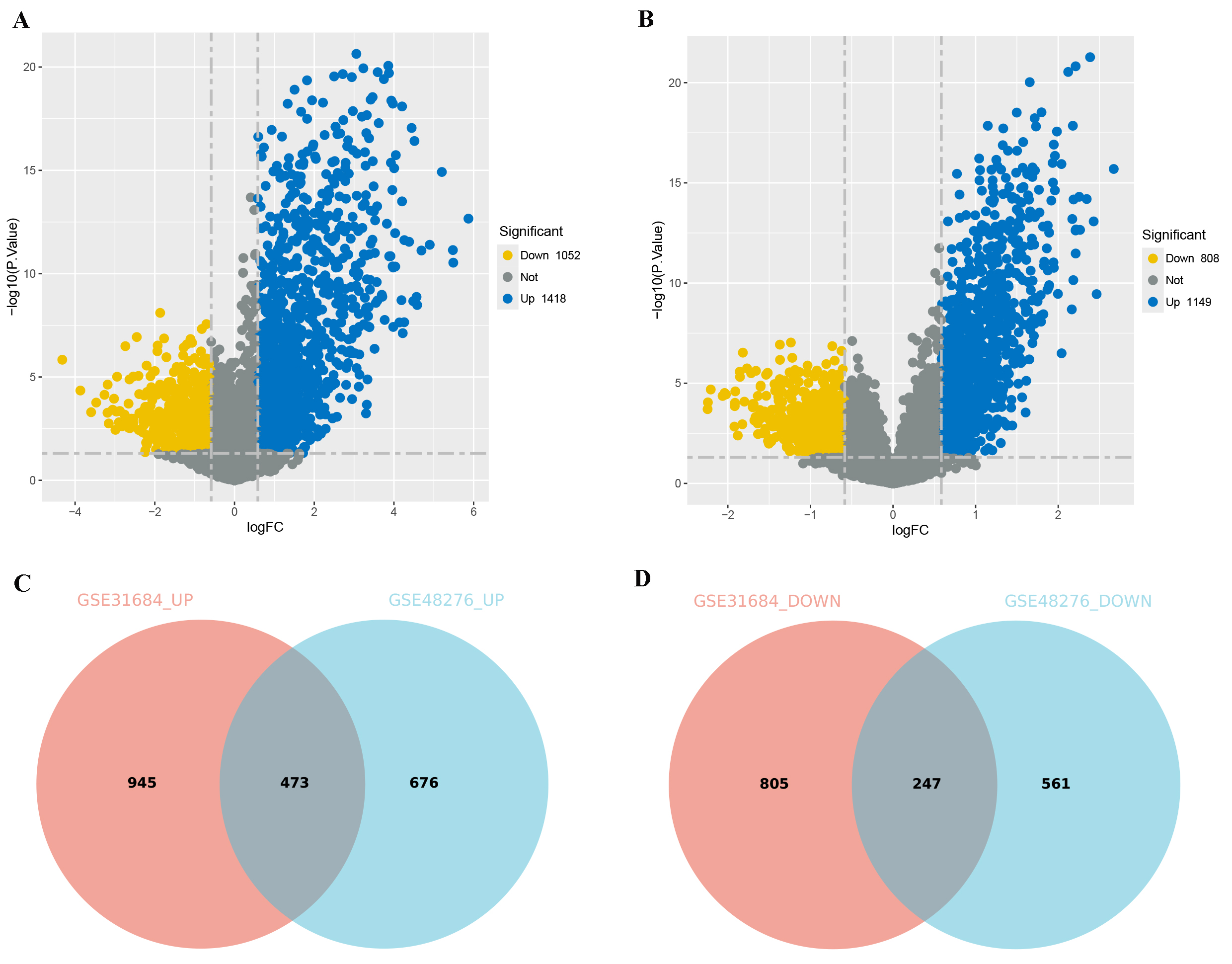

Supplement: Supplementary file 1 [file DataSheet1.zip › all raw data/Figures/Figure 3/Figure 3.jpg]

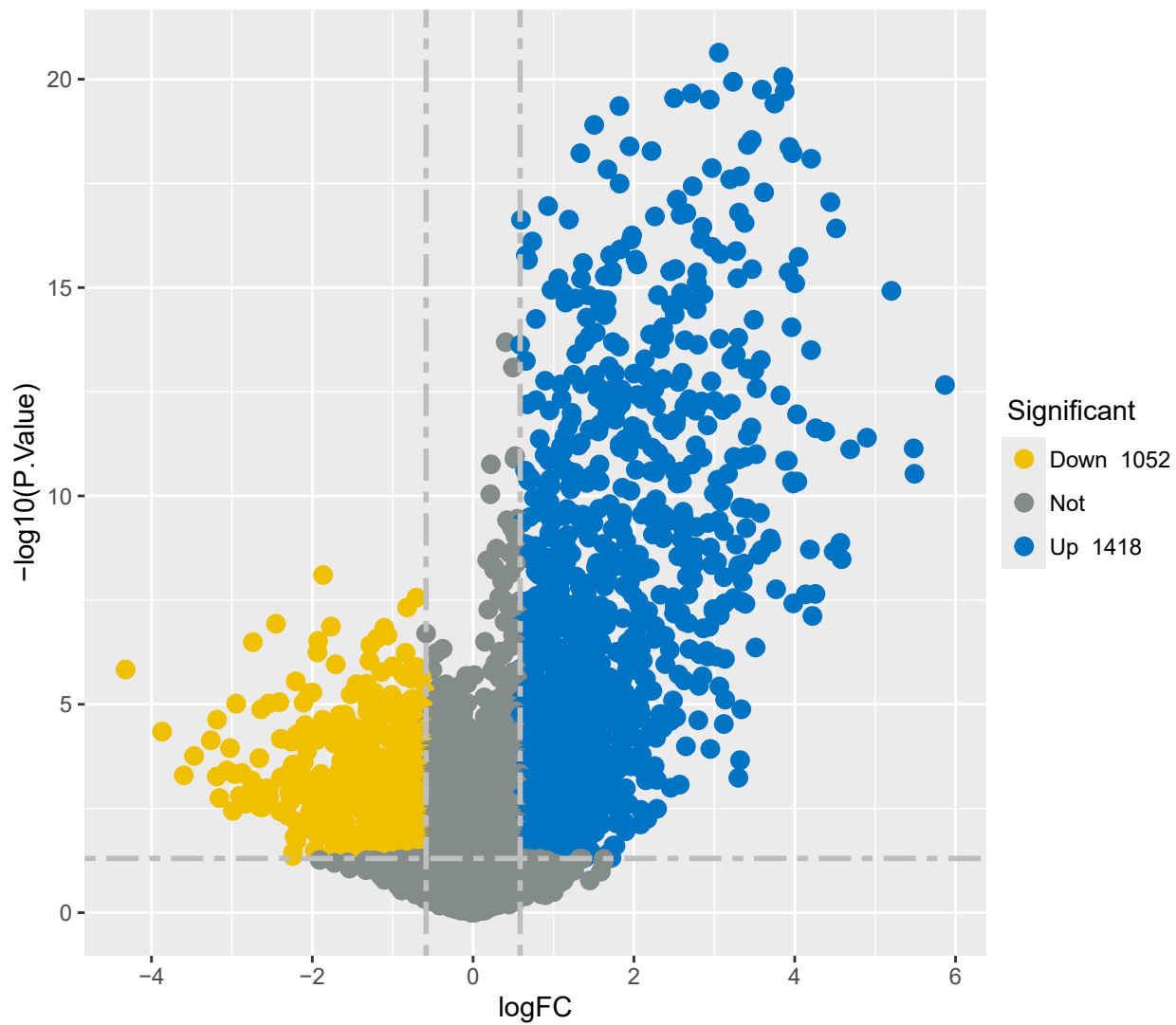

Supplement: Supplementary file 1 [file DataSheet1.zip › all raw data/Figures/Figure 3/Figure 3A.pdf]

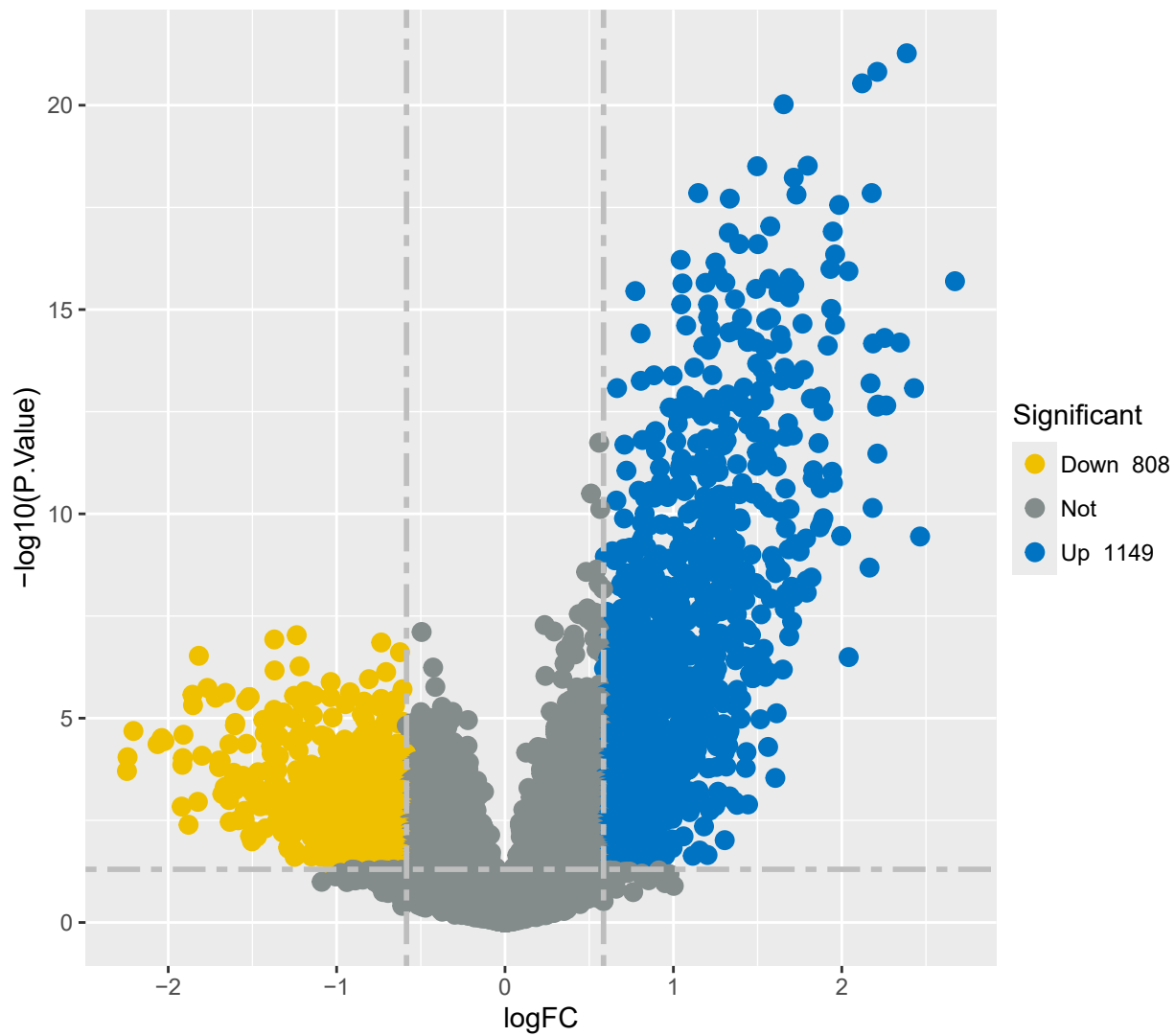

Supplement: Supplementary file 1 [file DataSheet1.zip › all raw data/Figures/Figure 3/Figure 3B.pdf]

GSE31684\_UP

GSE48276\_UP

**945**

**473**

**676**

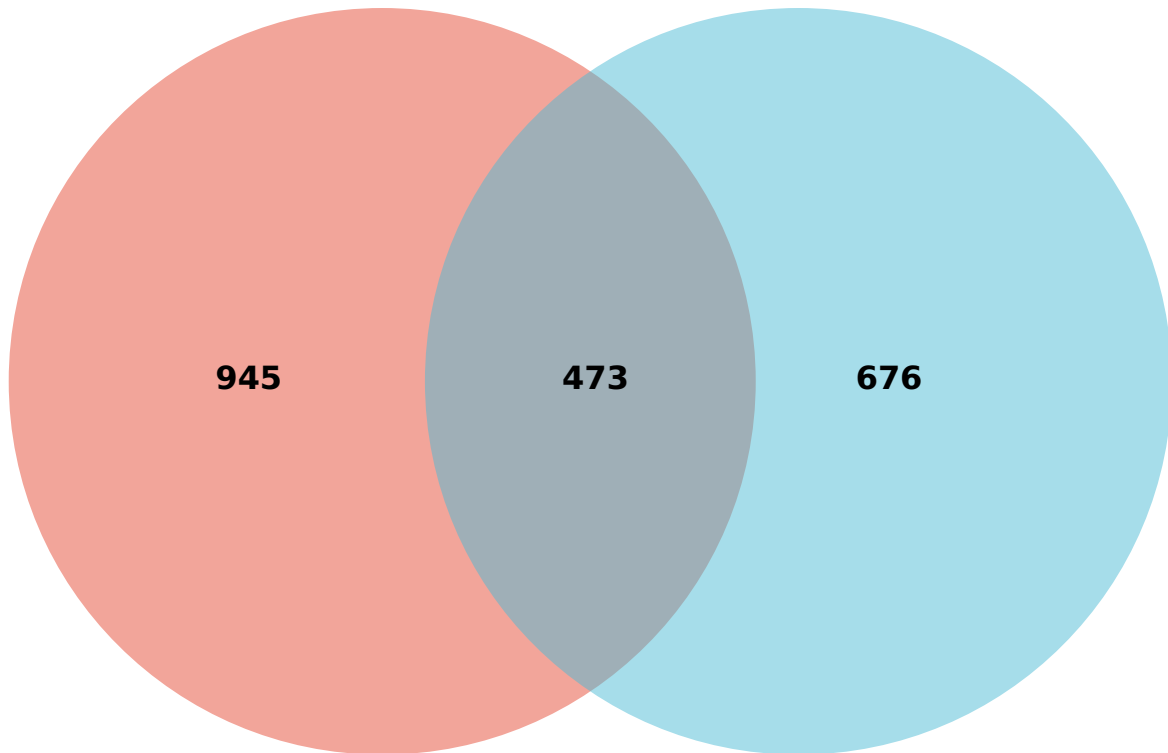

Supplement: Supplementary file 1 [file DataSheet1.zip › all raw data/Figures/Figure 3/Figure 3C.pdf]

GSE31684\_DOWN

GSE48276\_DOWN

**805**

**247**

**561**

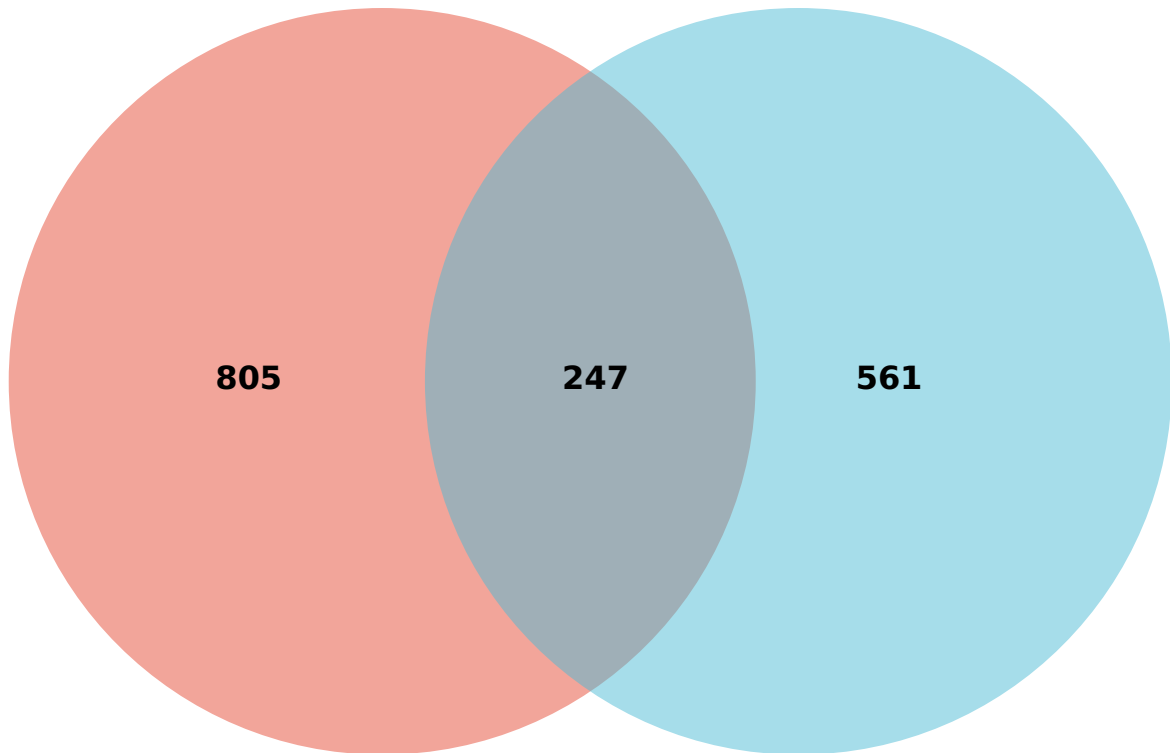

Supplement: Supplementary file 1 [file DataSheet1.zip › all raw data/Figures/Figure 3/Figure 3D.pdf]

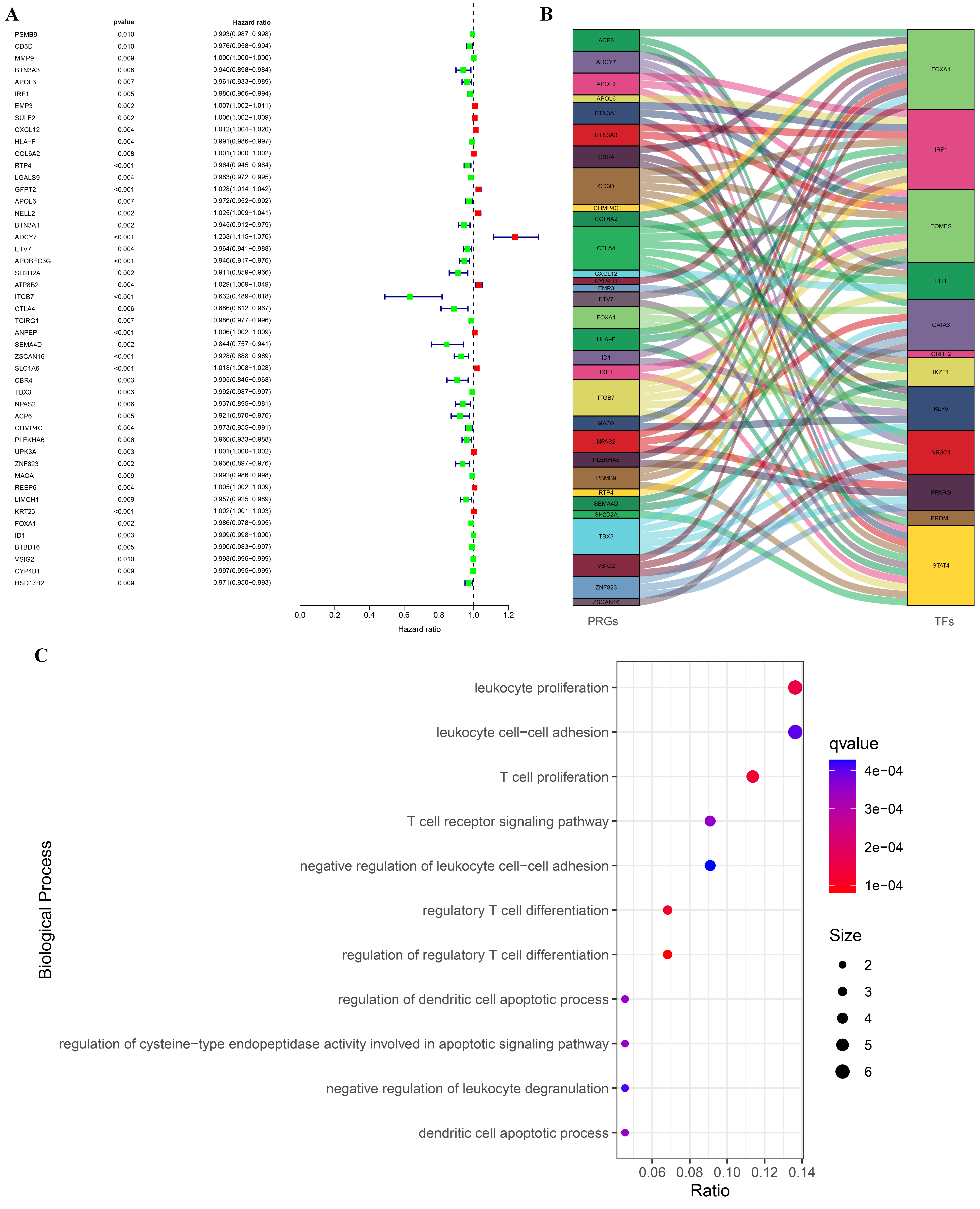

Supplement: Supplementary file 1 [file DataSheet1.zip › all raw data/Figures/Figure 4/Figure 4.jpg]

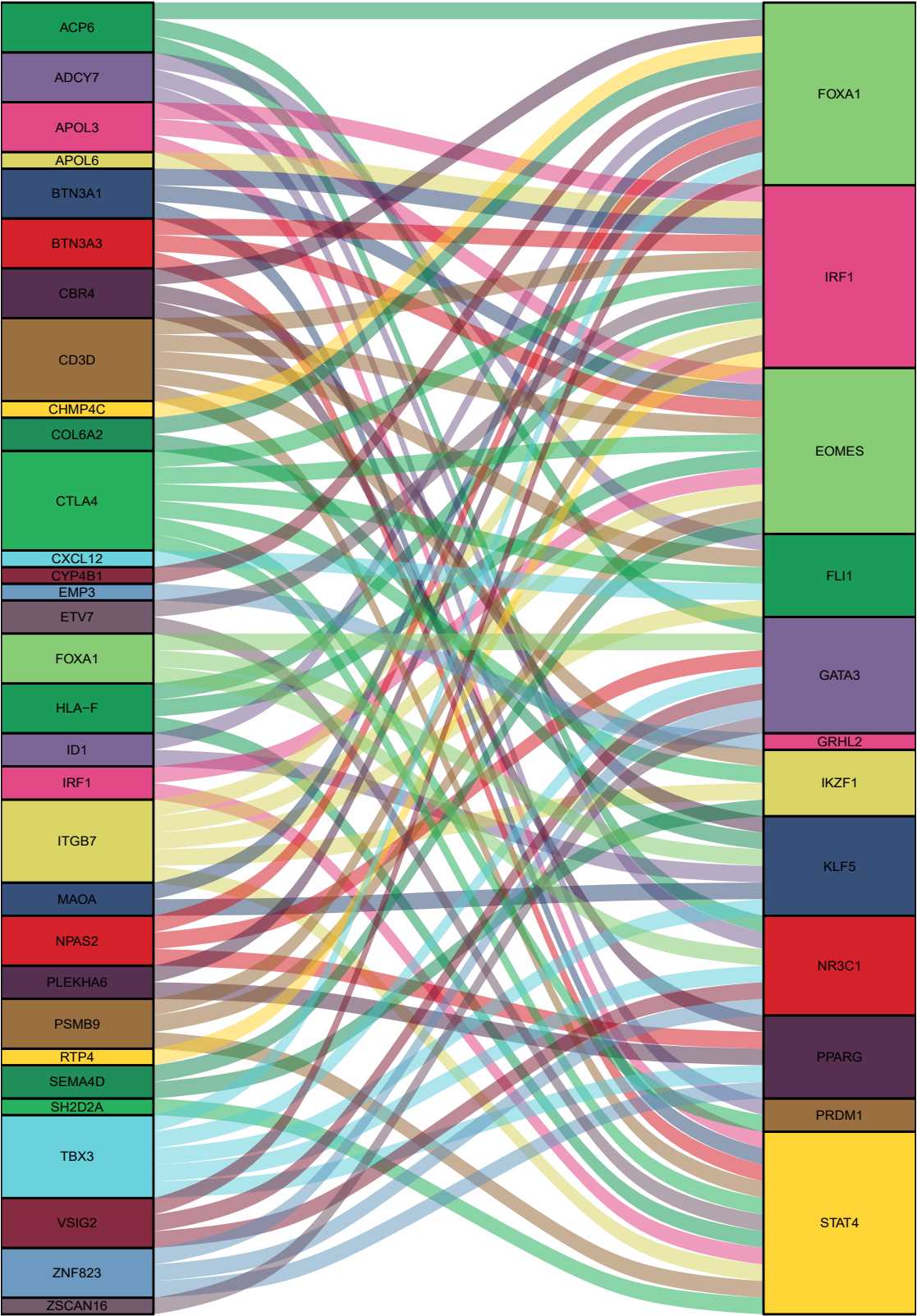

PRGs

TFs

Supplement: Supplementary file 1 [file DataSheet1.zip › all raw data/Figures/Figure 4/Figure 4B.pdf]

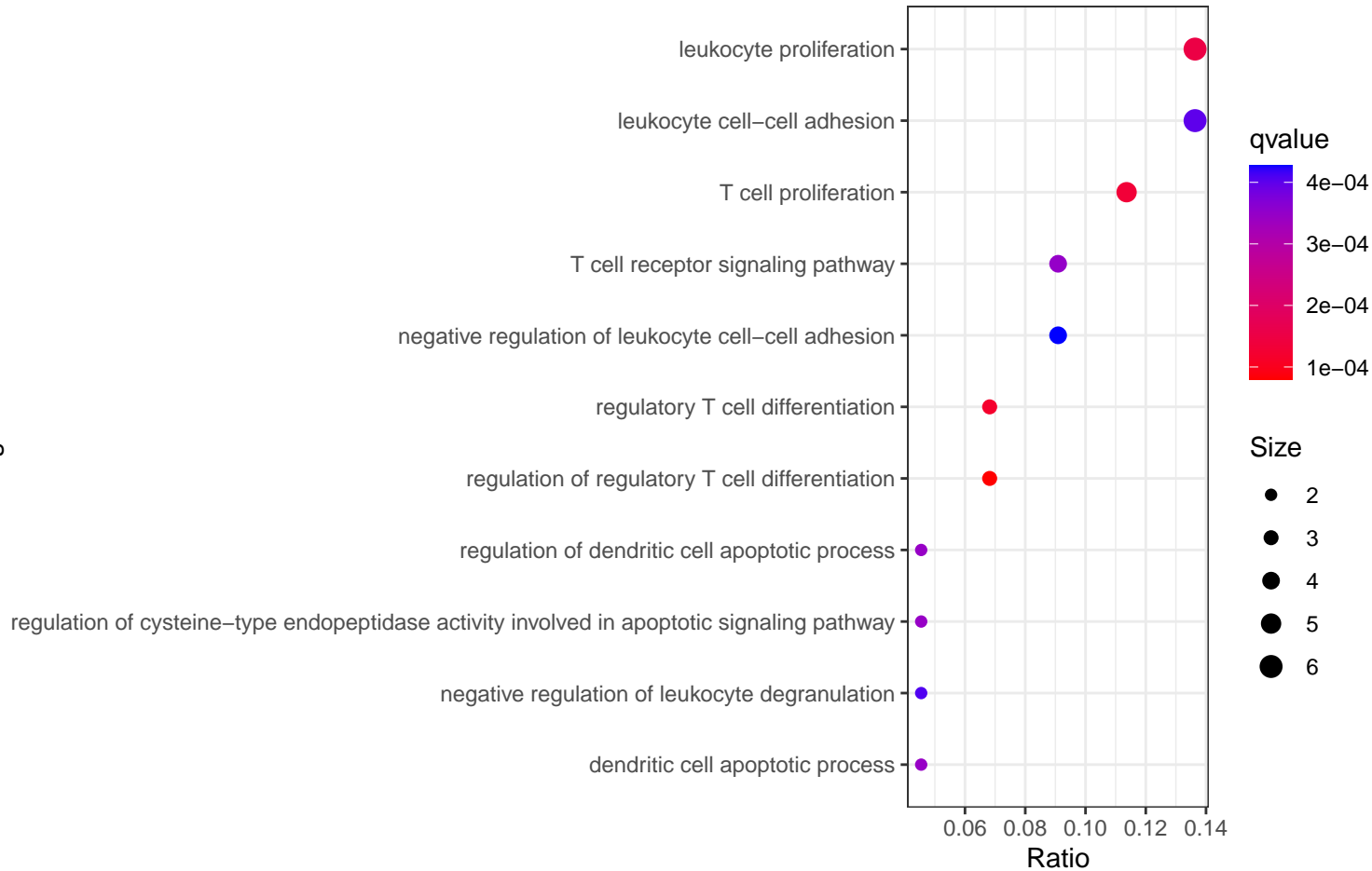

Supplement: Supplementary file 1 [file DataSheet1.zip › all raw data/Figures/Figure 4/Figure 4C.Pdf]

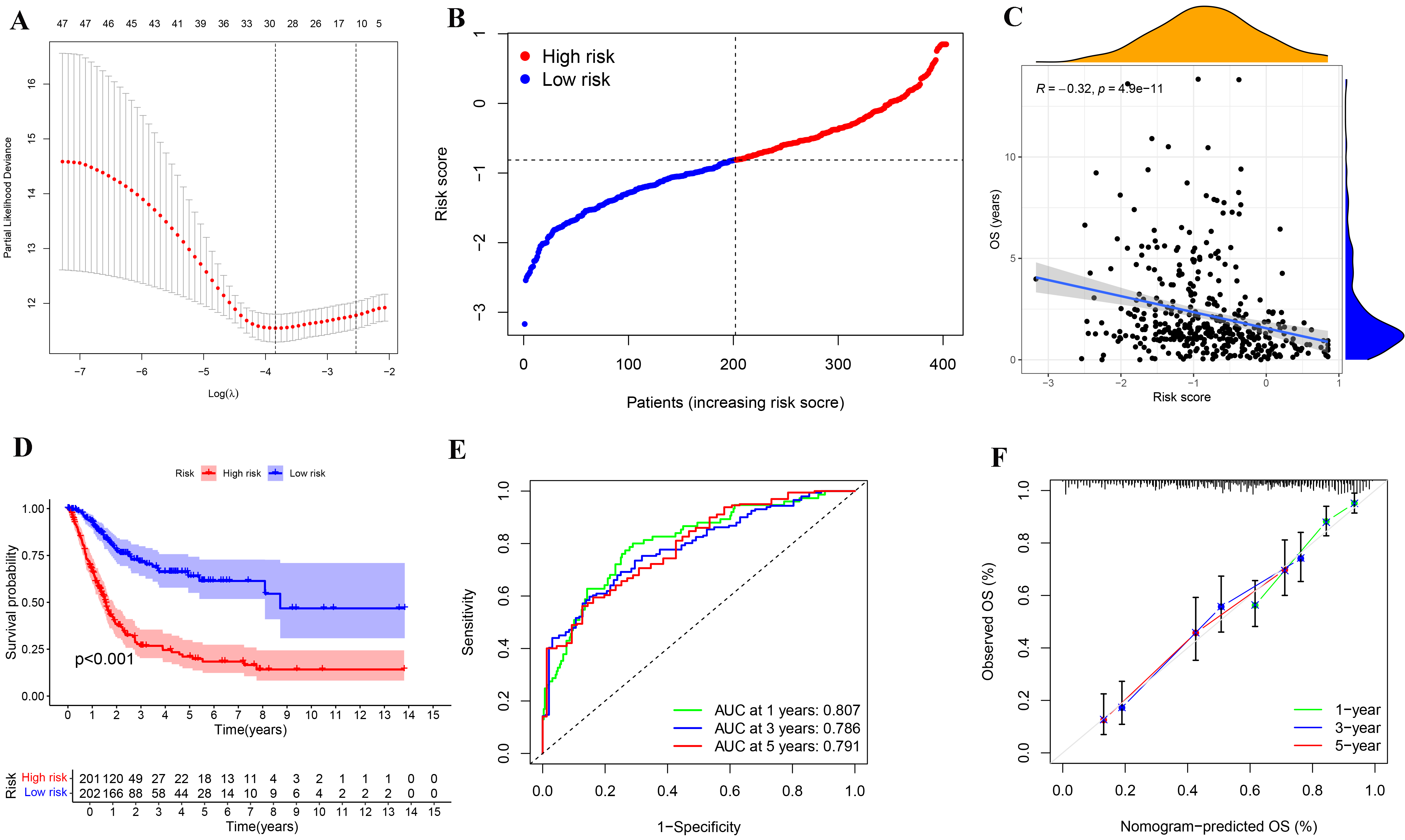

Supplement: Supplementary file 1 [file DataSheet1.zip › all raw data/Figures/Figure 5/Figure 5.jpg]

Coefficients

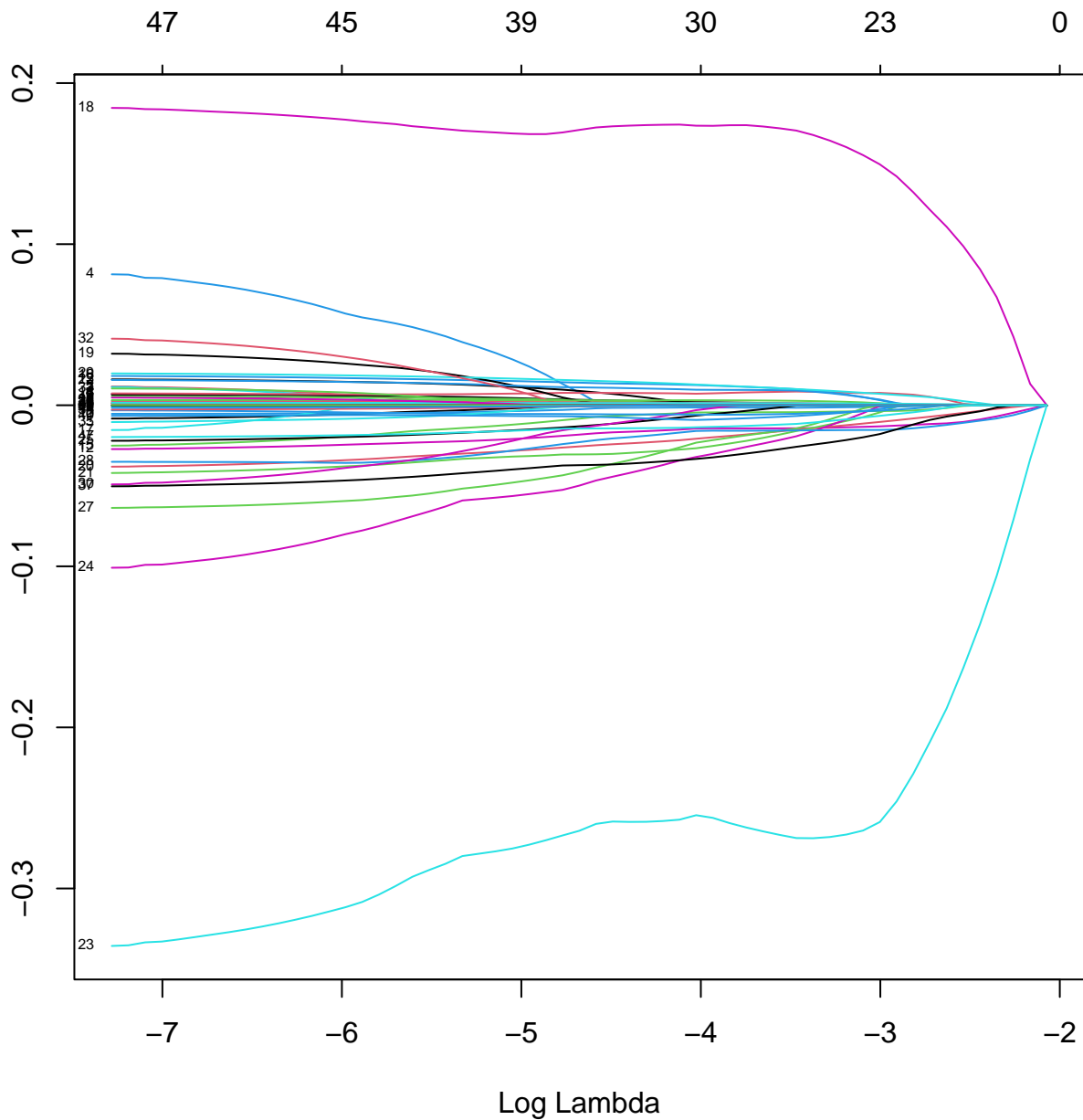

Supplement: Supplementary file 1 [file DataSheet1.zip › all raw data/Figures/Figure 5/Figure 5A.pdf]

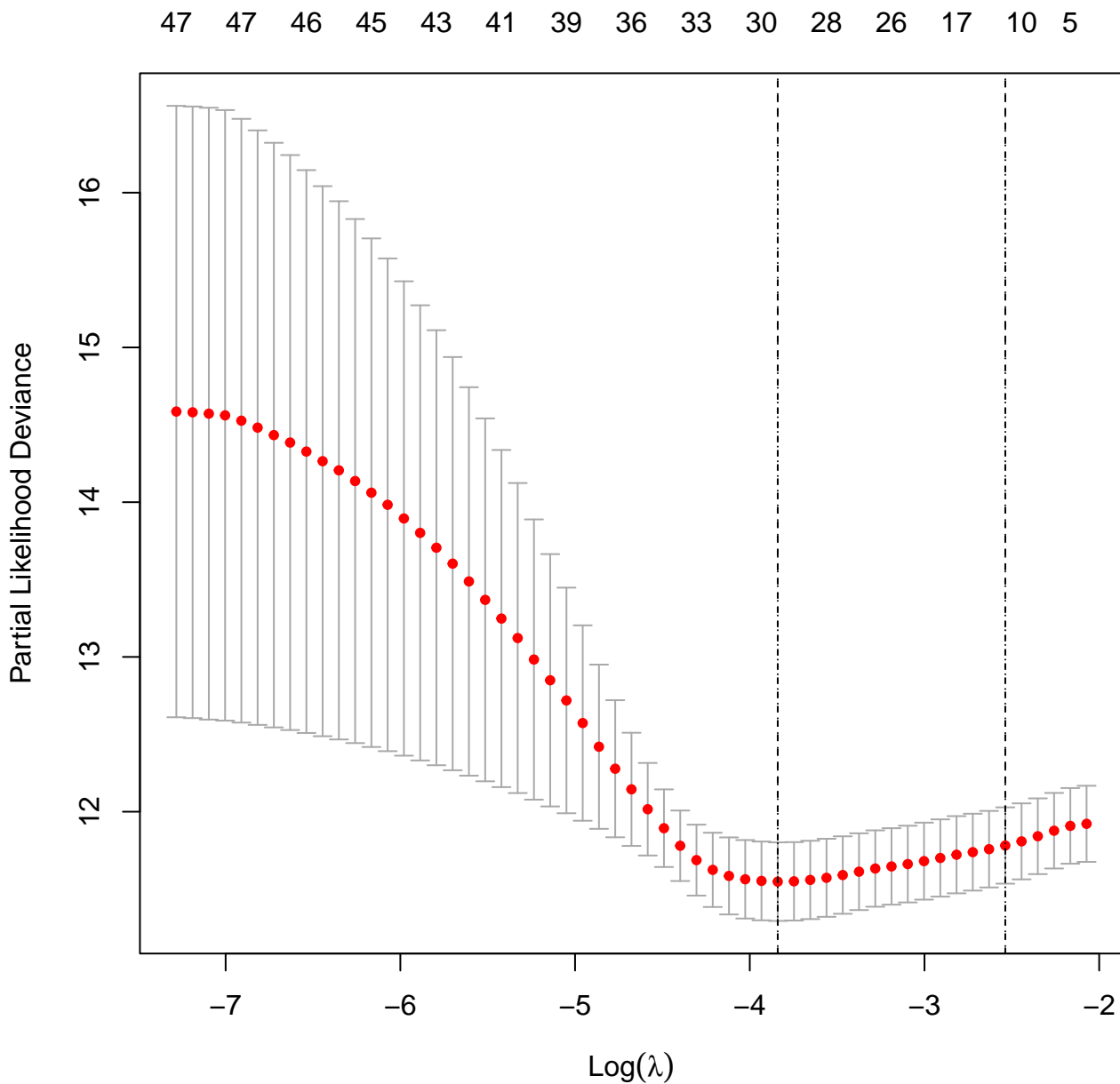

Supplement: Supplementary file 1 [file DataSheet1.zip › all raw data/Figures/Figure 5/Figure 5B.pdf]

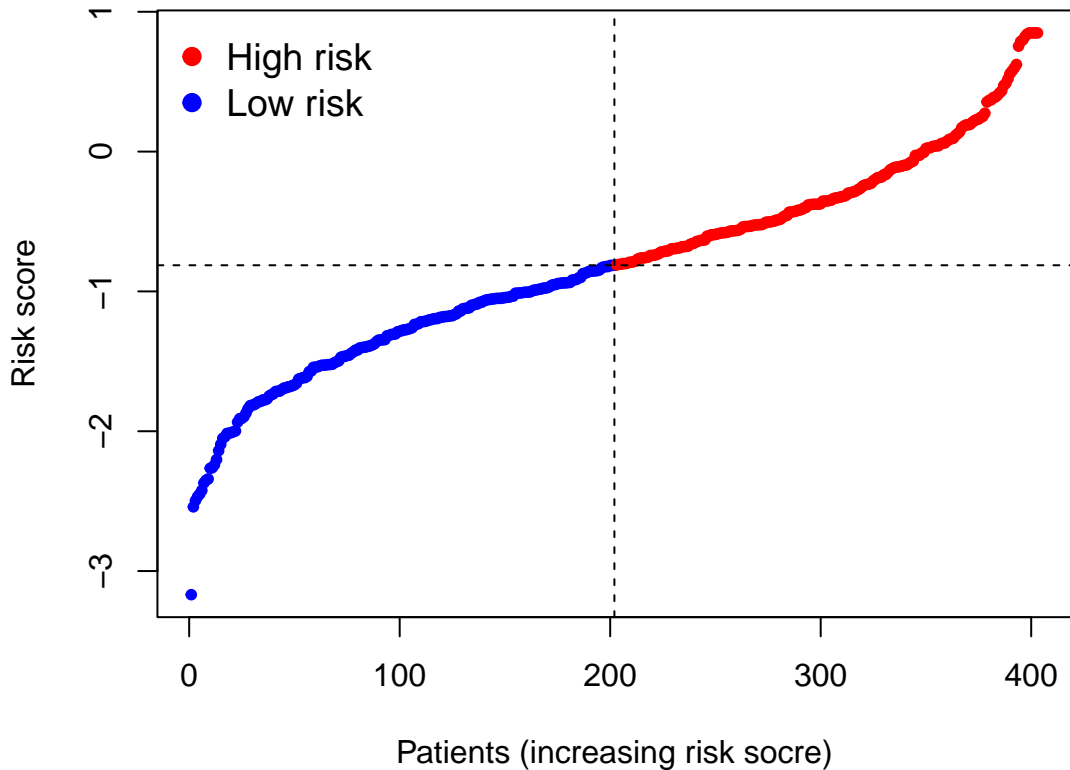

Supplement: Supplementary file 1 [file DataSheet1.zip › all raw data/Figures/Figure 5/Figure 5C.pdf]

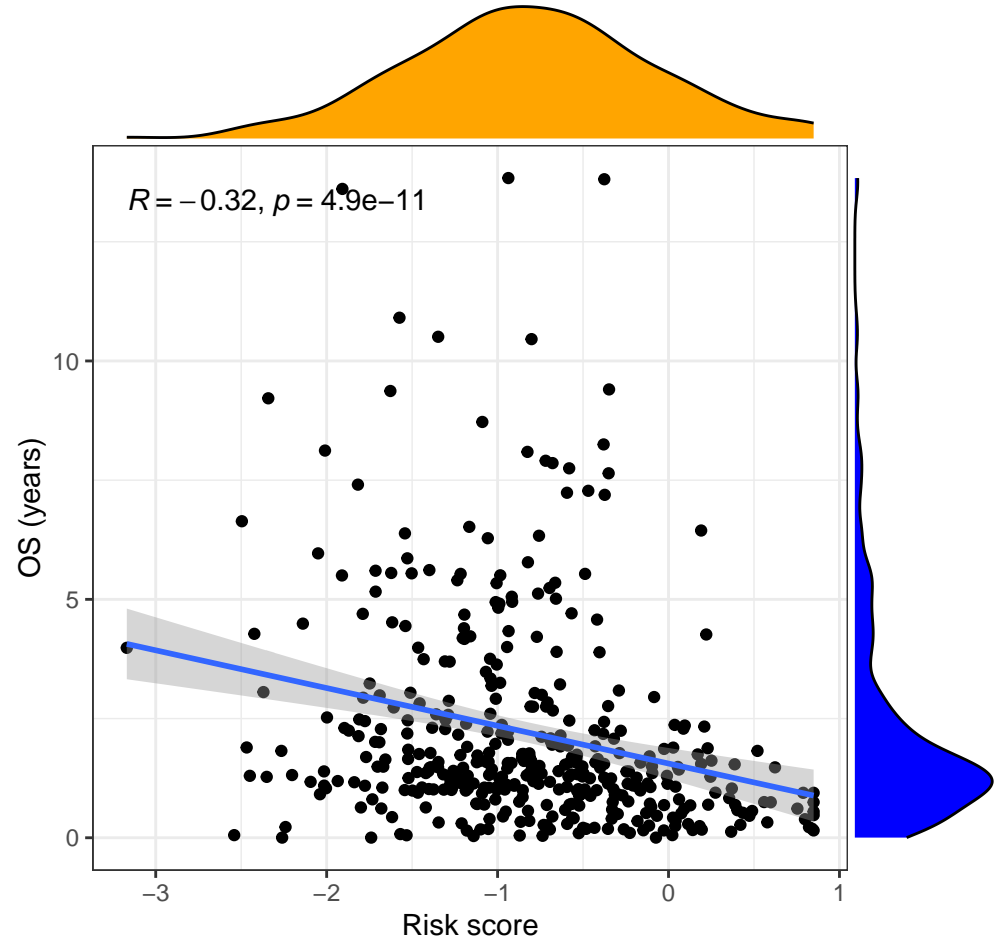

Supplement: Supplementary file 1 [file DataSheet1.zip › all raw data/Figures/Figure 5/Figure 5D.pdf]

Risk + High risk + Low risk

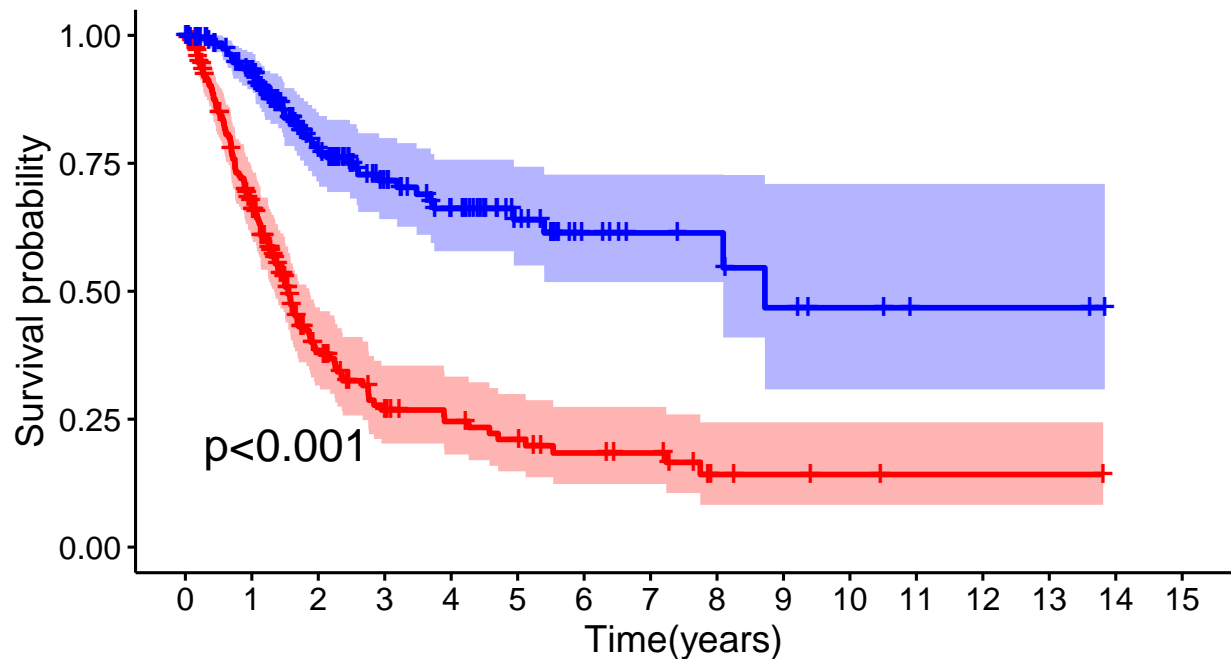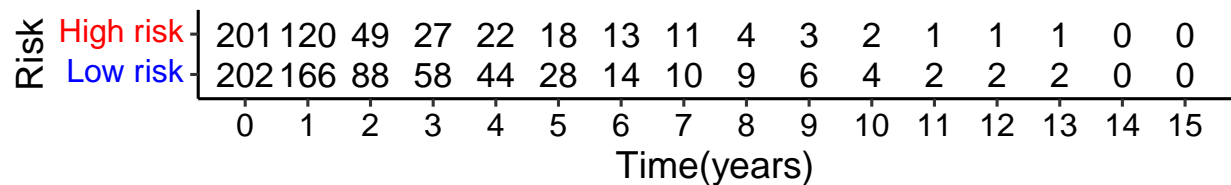

Supplement: Supplementary file 1 [file DataSheet1.zip › all raw data/Figures/Figure 5/Figure 5E.pdf]

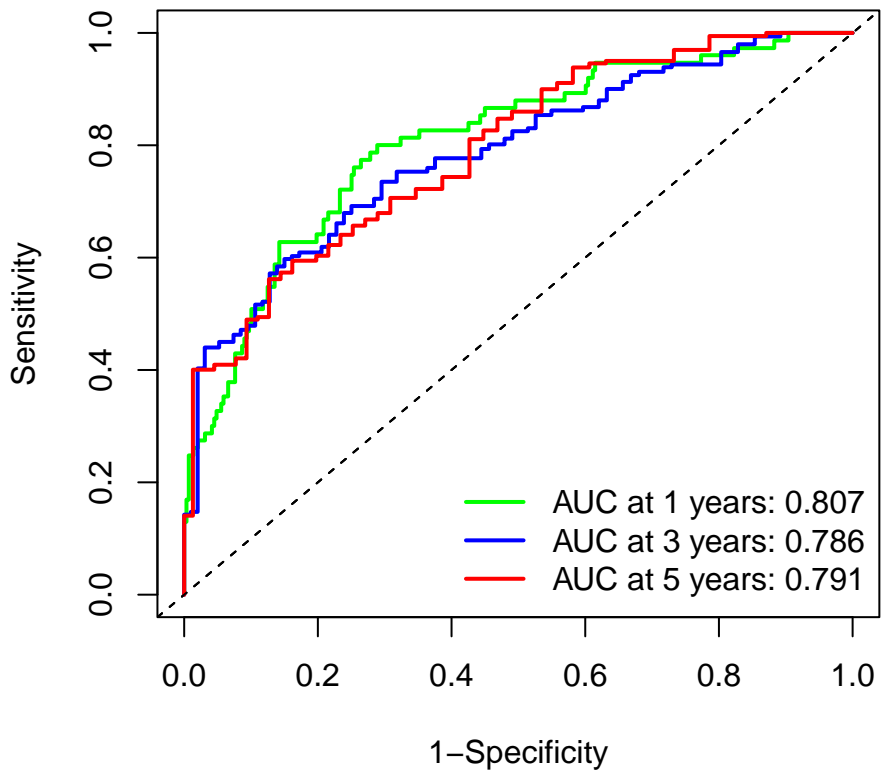

Supplement: Supplementary file 1 [file DataSheet1.zip › all raw data/Figures/Figure 5/Figure 5F.pdf]

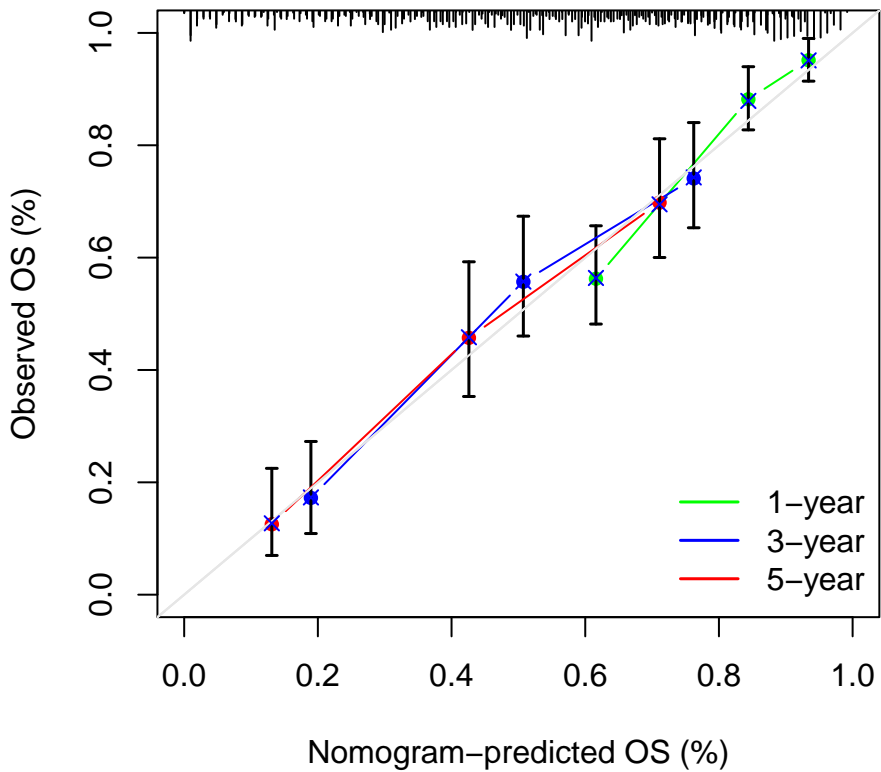

Supplement: Supplementary file 1 [file DataSheet1.zip › all raw data/Figures/Figure 5/Figure 5G.pdf]

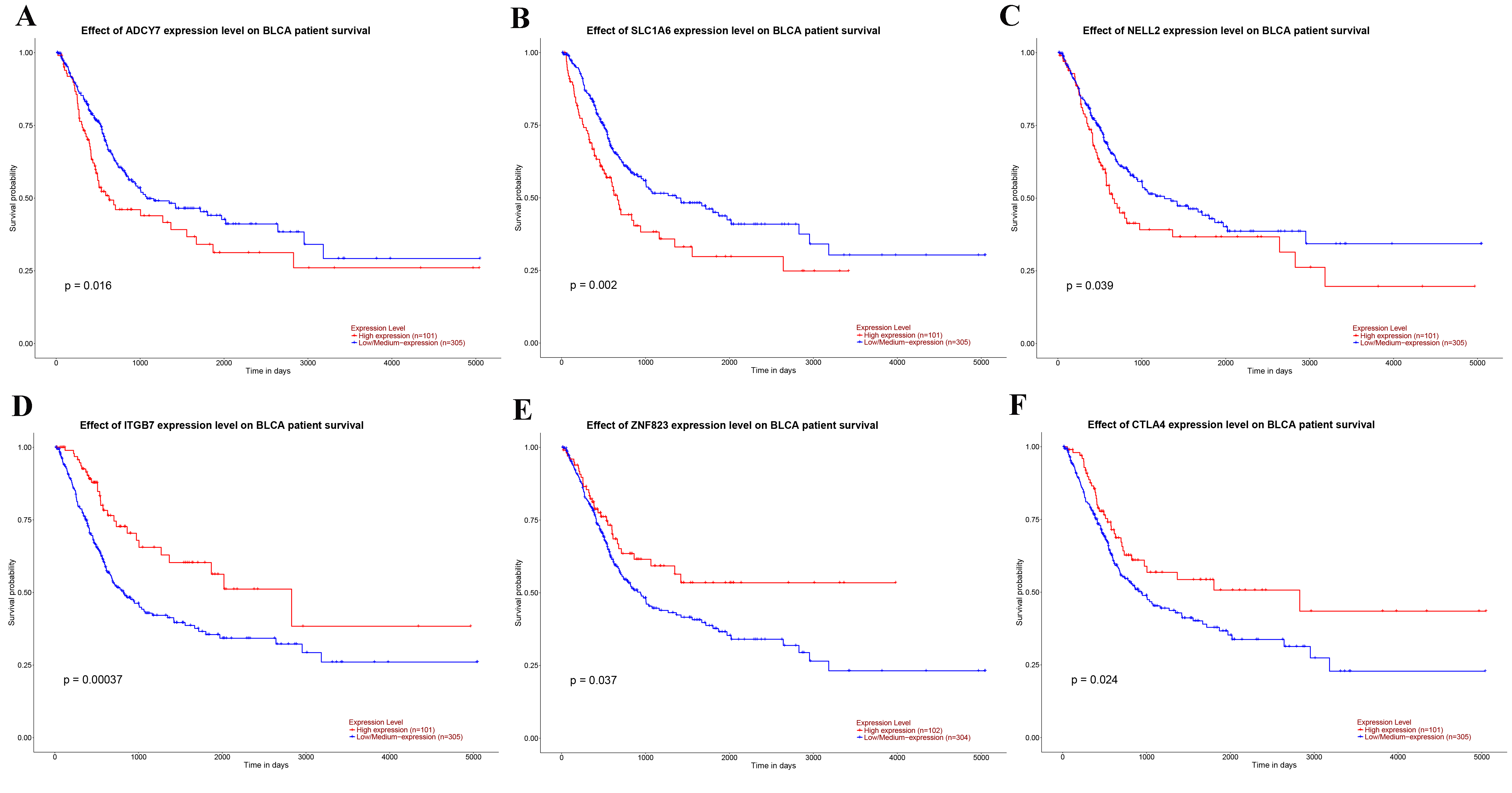

Supplement: Supplementary file 1 [file DataSheet1.zip › all raw data/Figures/Figure 6/Figure 6.jpg]

# Effect of ADCY7 expression level on BLCA patient survival

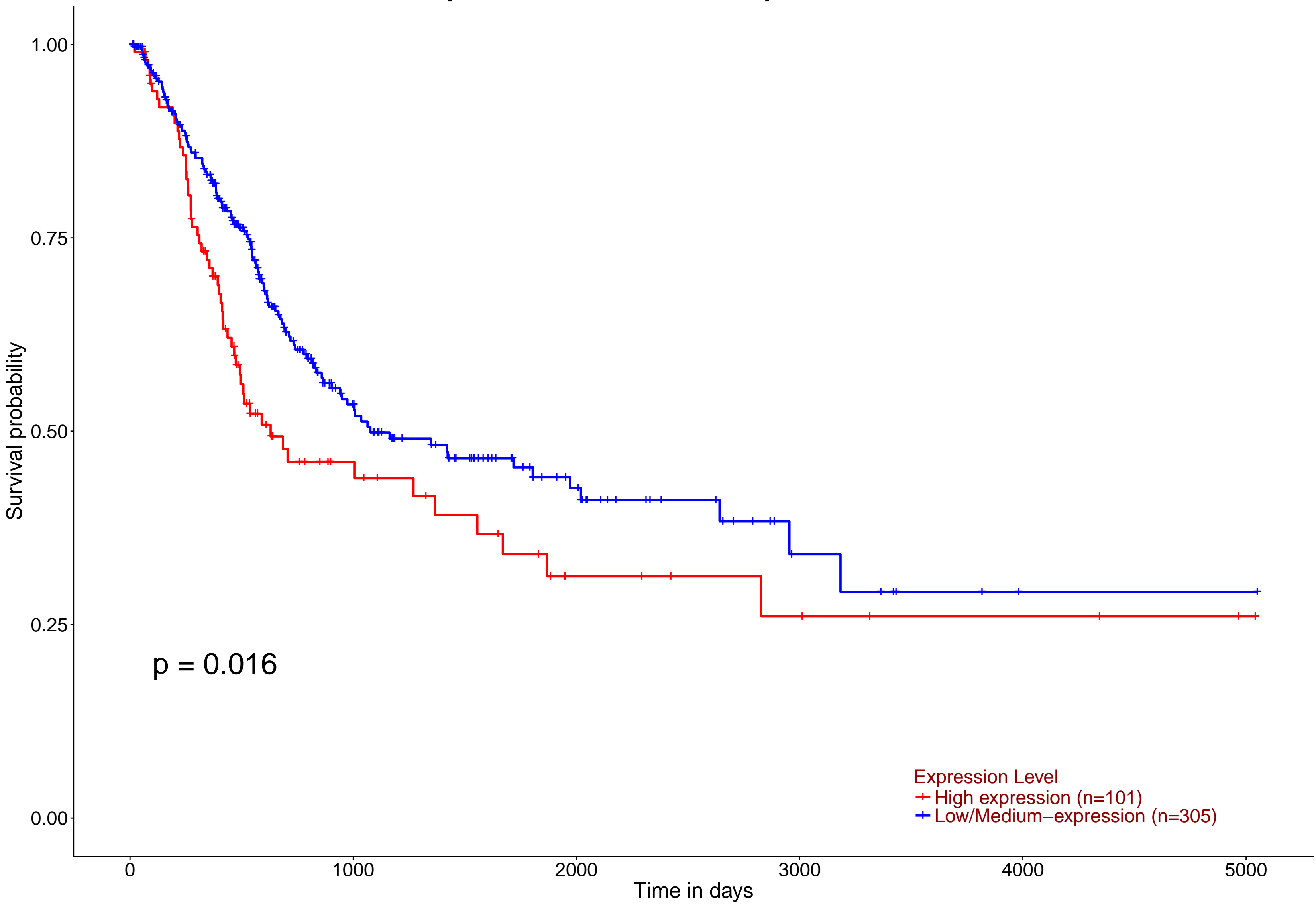

Supplement: Supplementary file 1 [file DataSheet1.zip › all raw data/Figures/Figure 6/Figure 6A.pdf]

# Effect of SLC1A6 expression level on BLCA patient survival

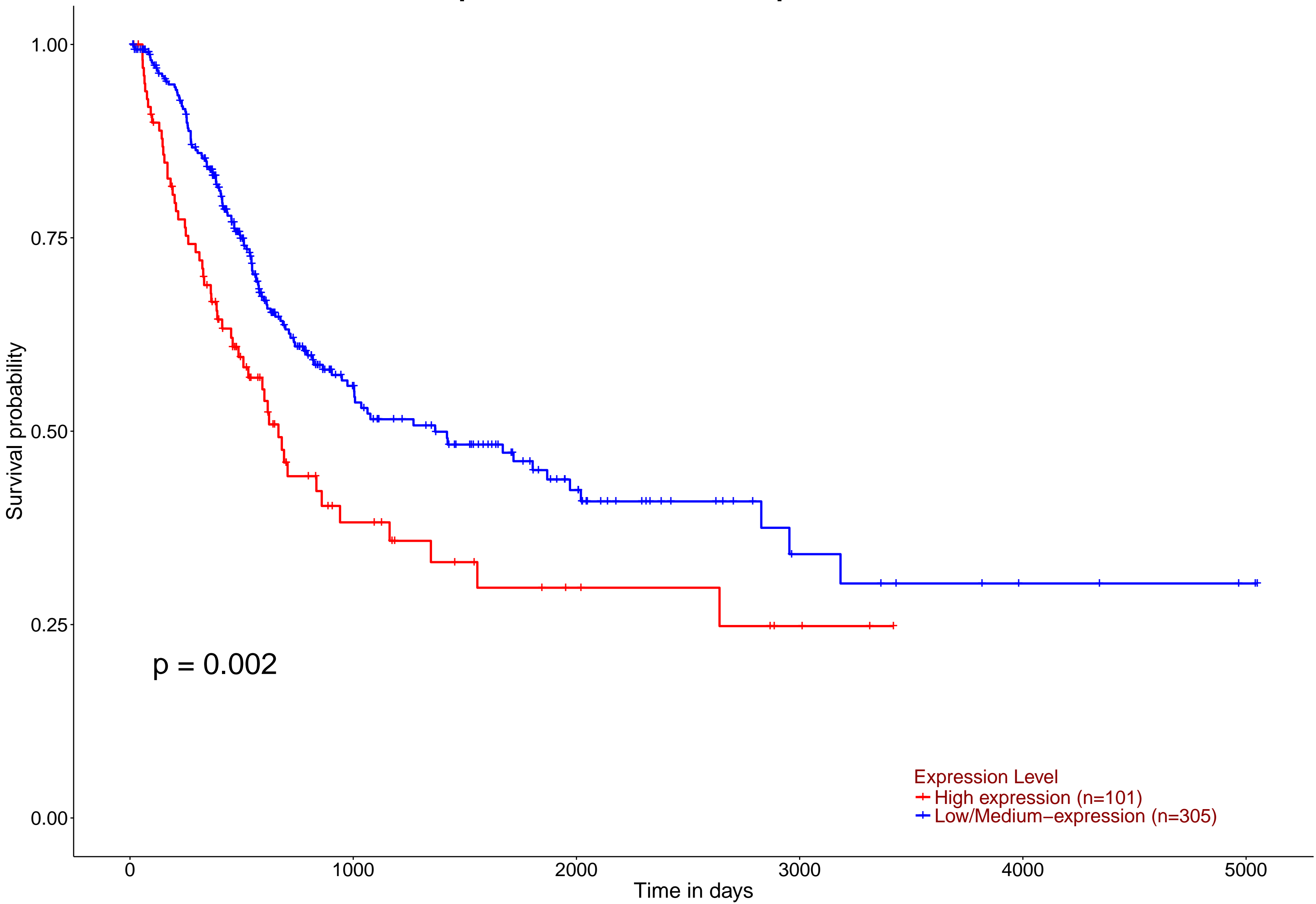

Supplement: Supplementary file 1 [file DataSheet1.zip › all raw data/Figures/Figure 6/Figure 6B.pdf]

Effect of NELL2 expression level on BLCA patient survival

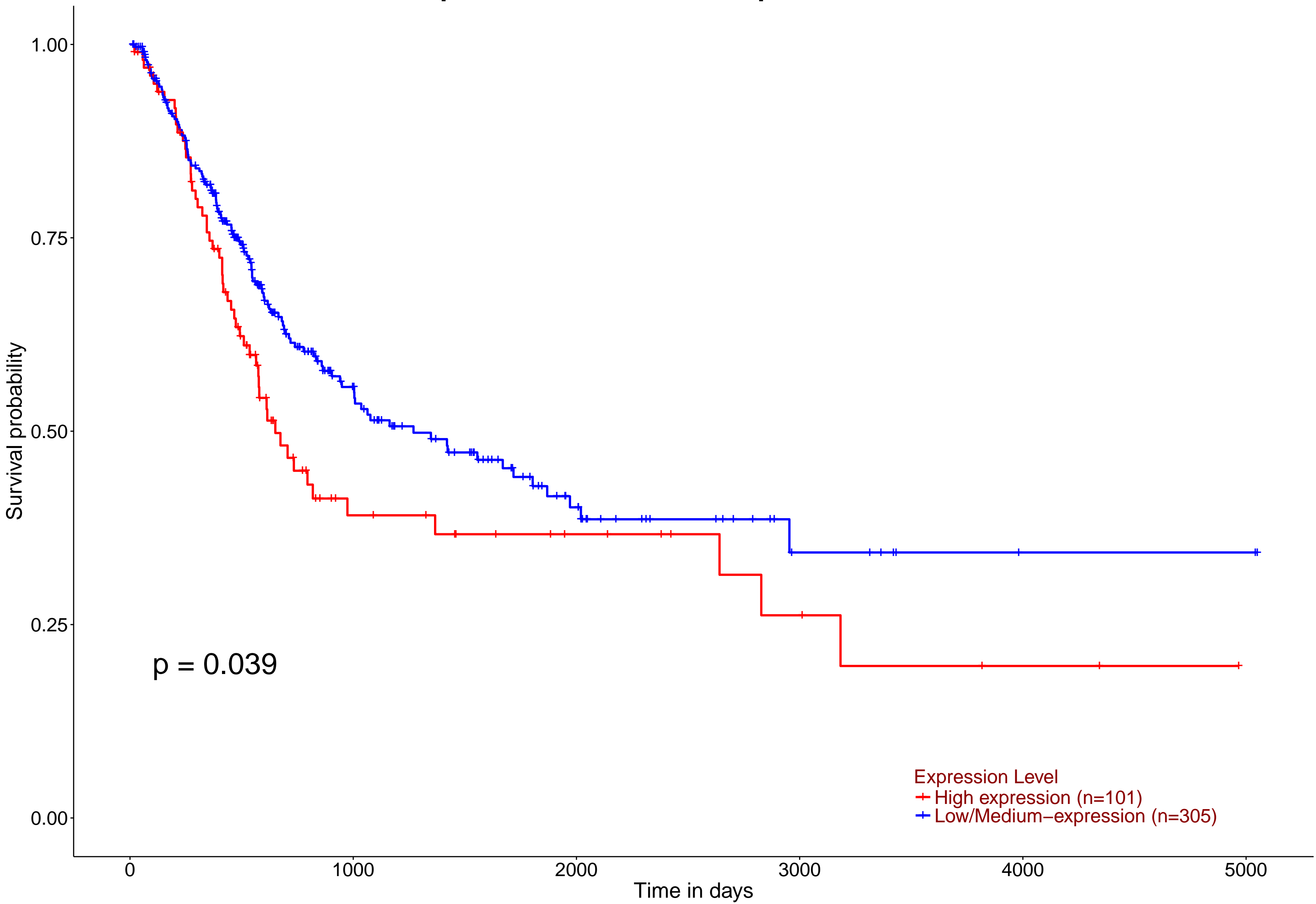

Supplement: Supplementary file 1 [file DataSheet1.zip › all raw data/Figures/Figure 6/Figure 6C.pdf]

# Effect of ITGB7 expression level on BLCA patient survival

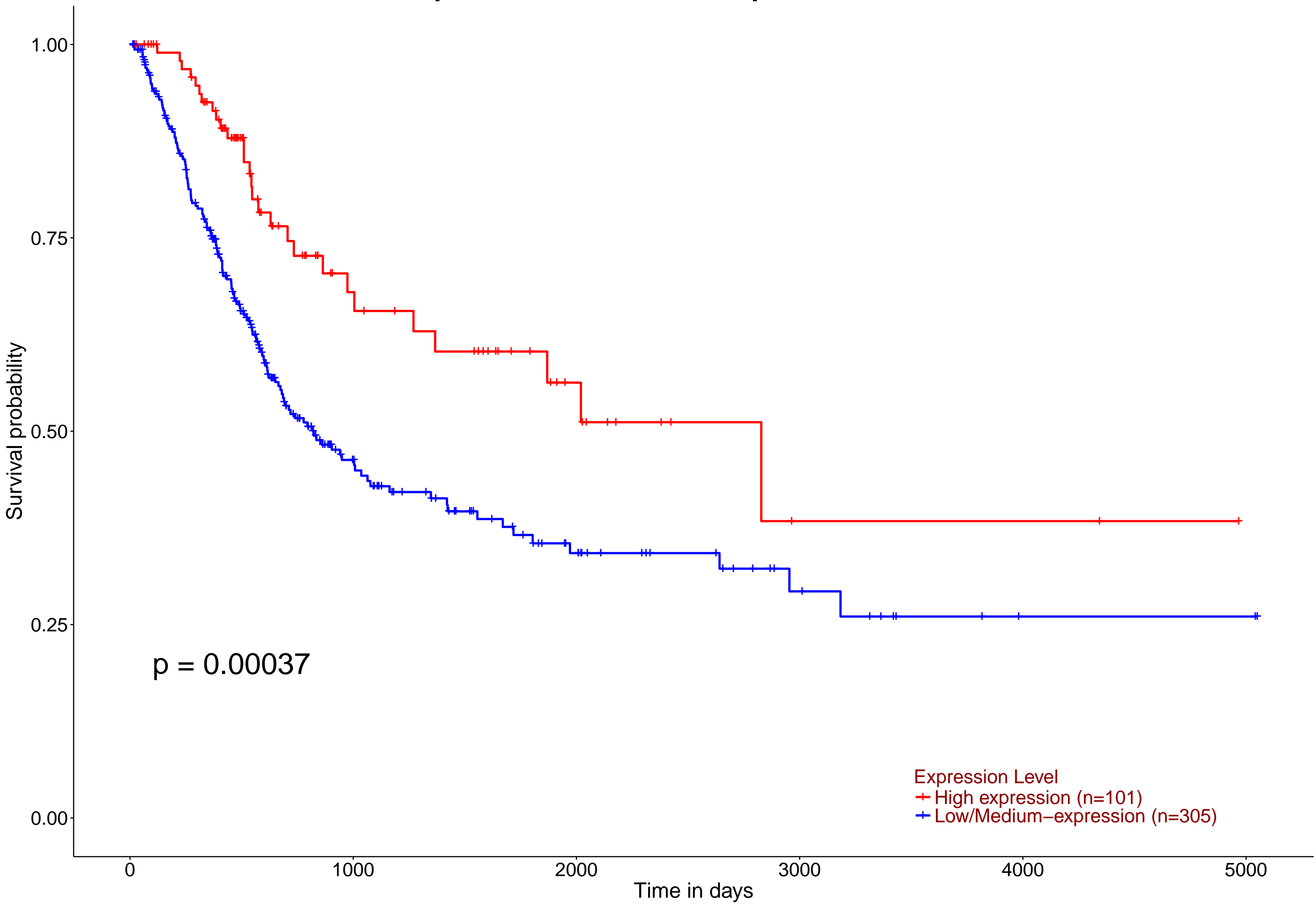

Supplement: Supplementary file 1 [file DataSheet1.zip › all raw data/Figures/Figure 6/Figure 6D.pdf]

# Effect of ZNF823 expression level on BLCA patient survival

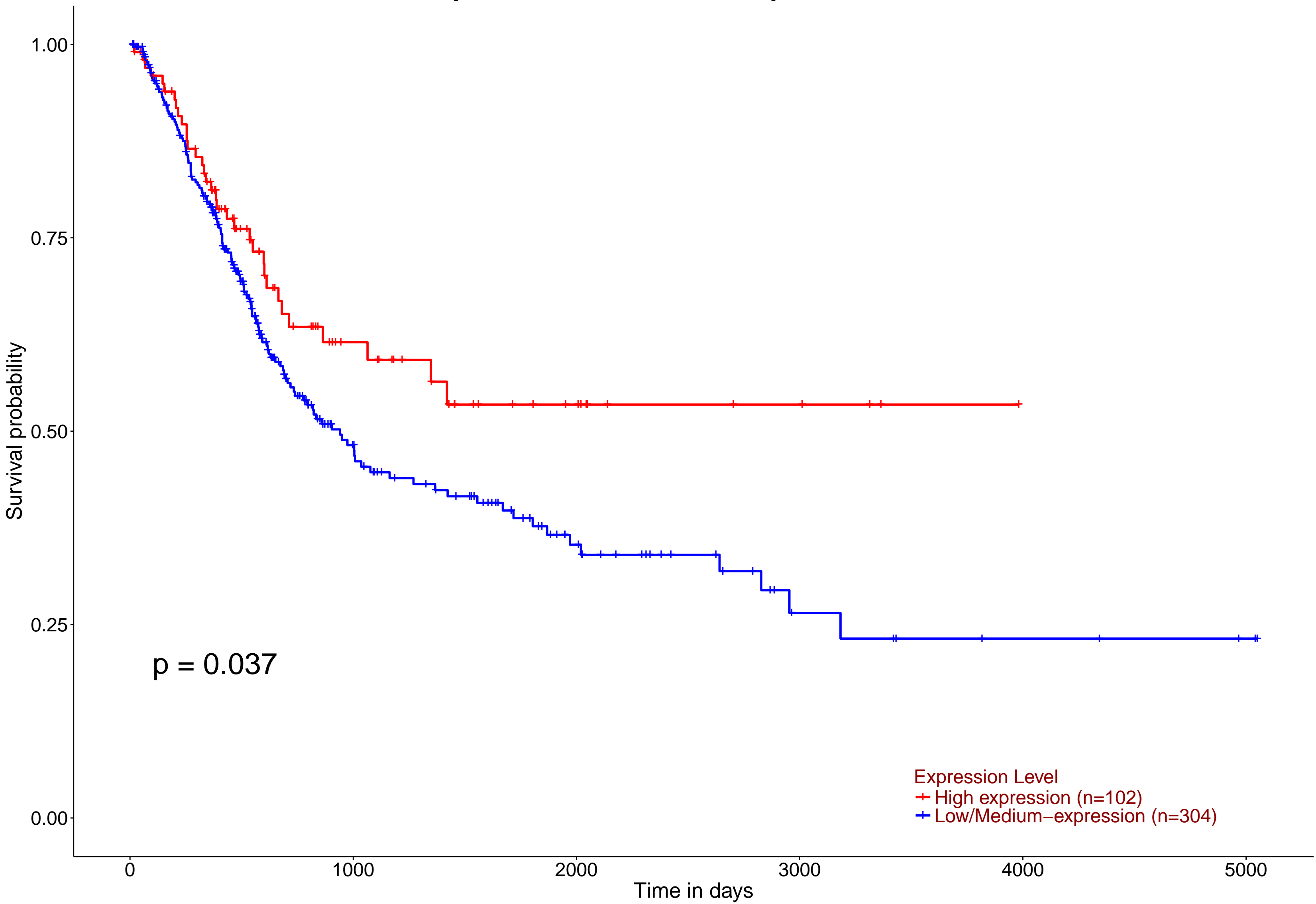

Supplement: Supplementary file 1 [file DataSheet1.zip › all raw data/Figures/Figure 6/Figure 6E.pdf]

# Effect of CTLA4 expression level on BLCA patient survival

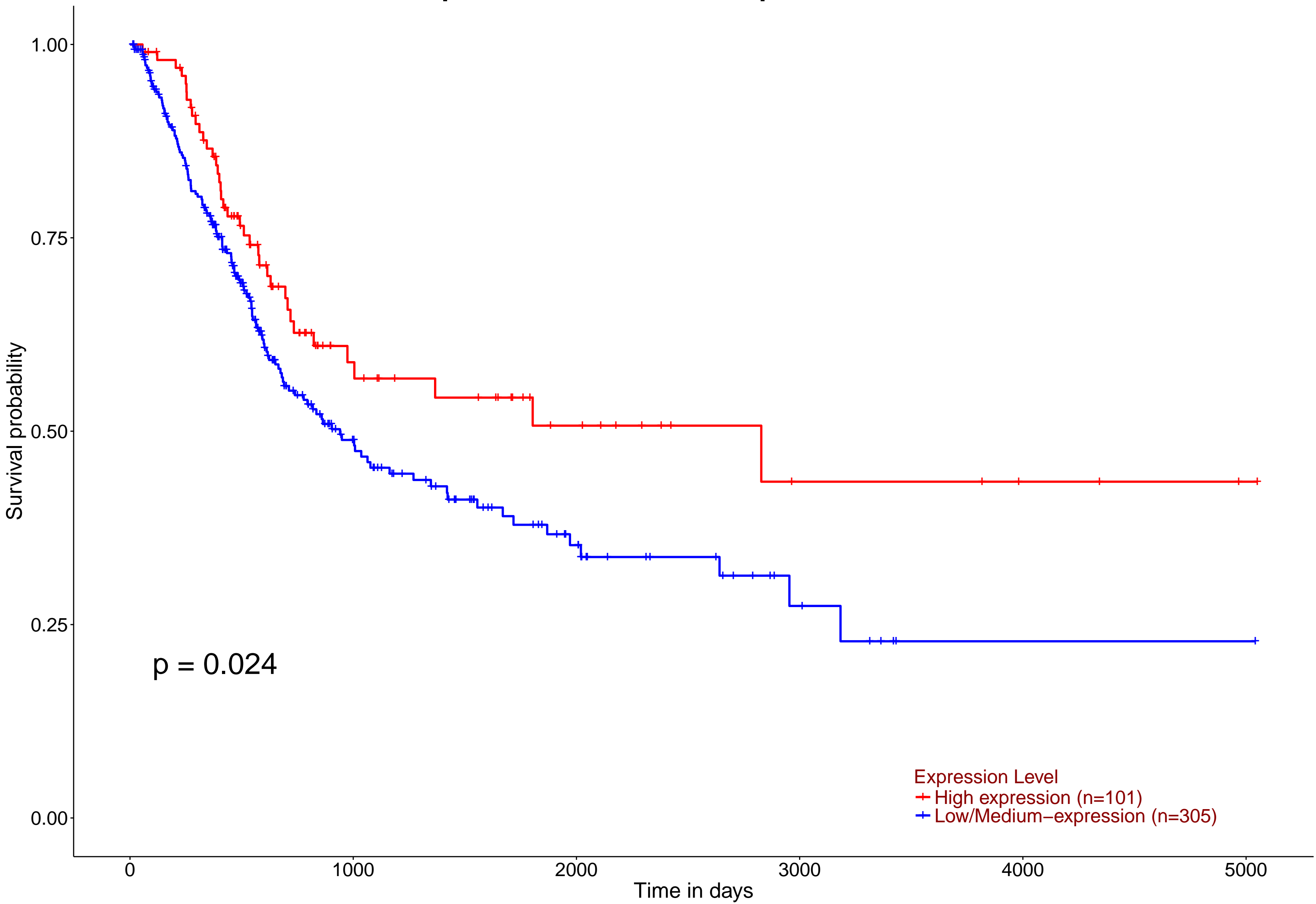

Supplement: Supplementary file 1 [file DataSheet1.zip › all raw data/Figures/Figure 6/Figure 6F.pdf]

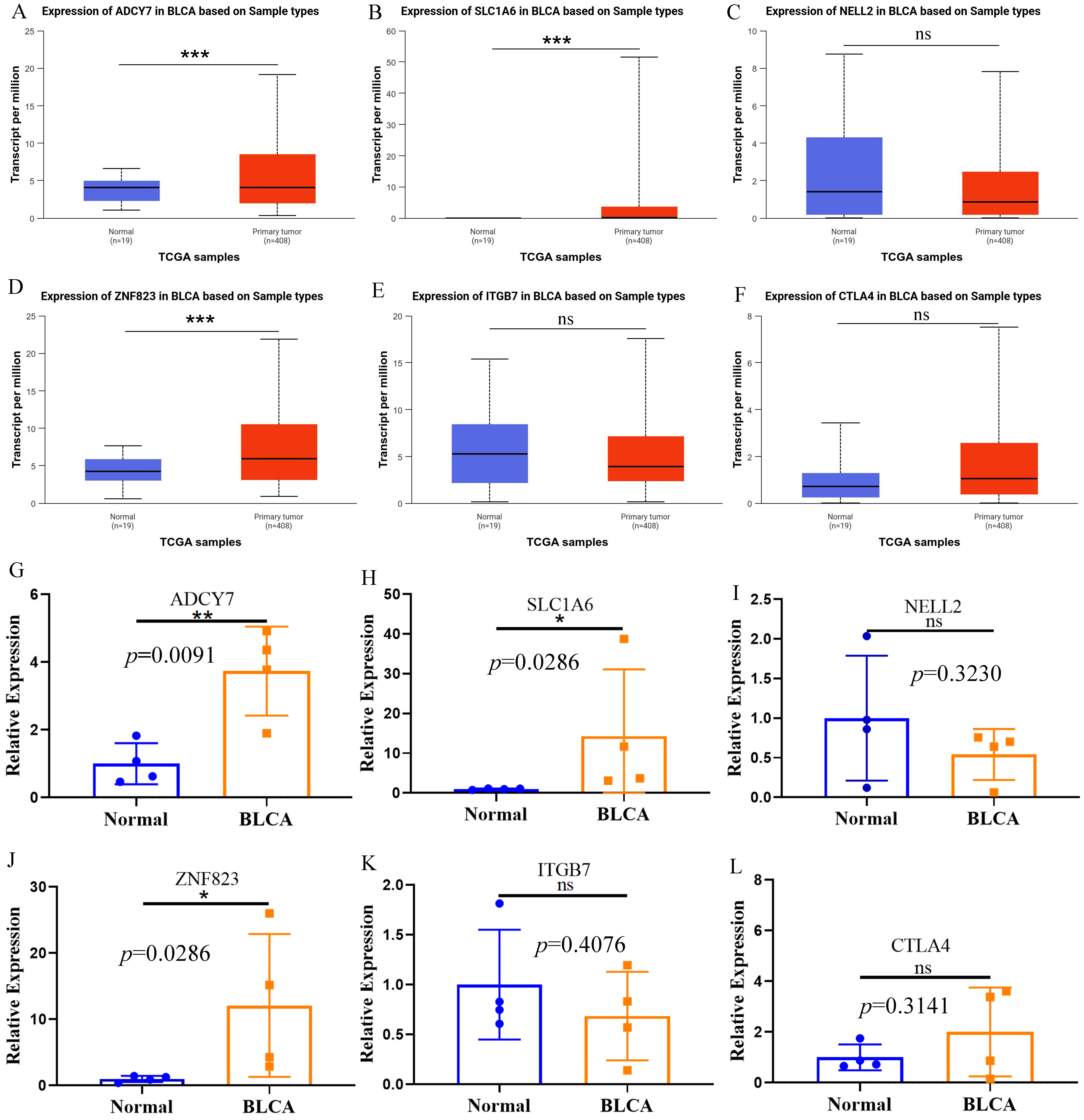

Supplement: Supplementary file 1 [file DataSheet1.zip › all raw data/Figures/Figure 7/Figure 7.jpg]

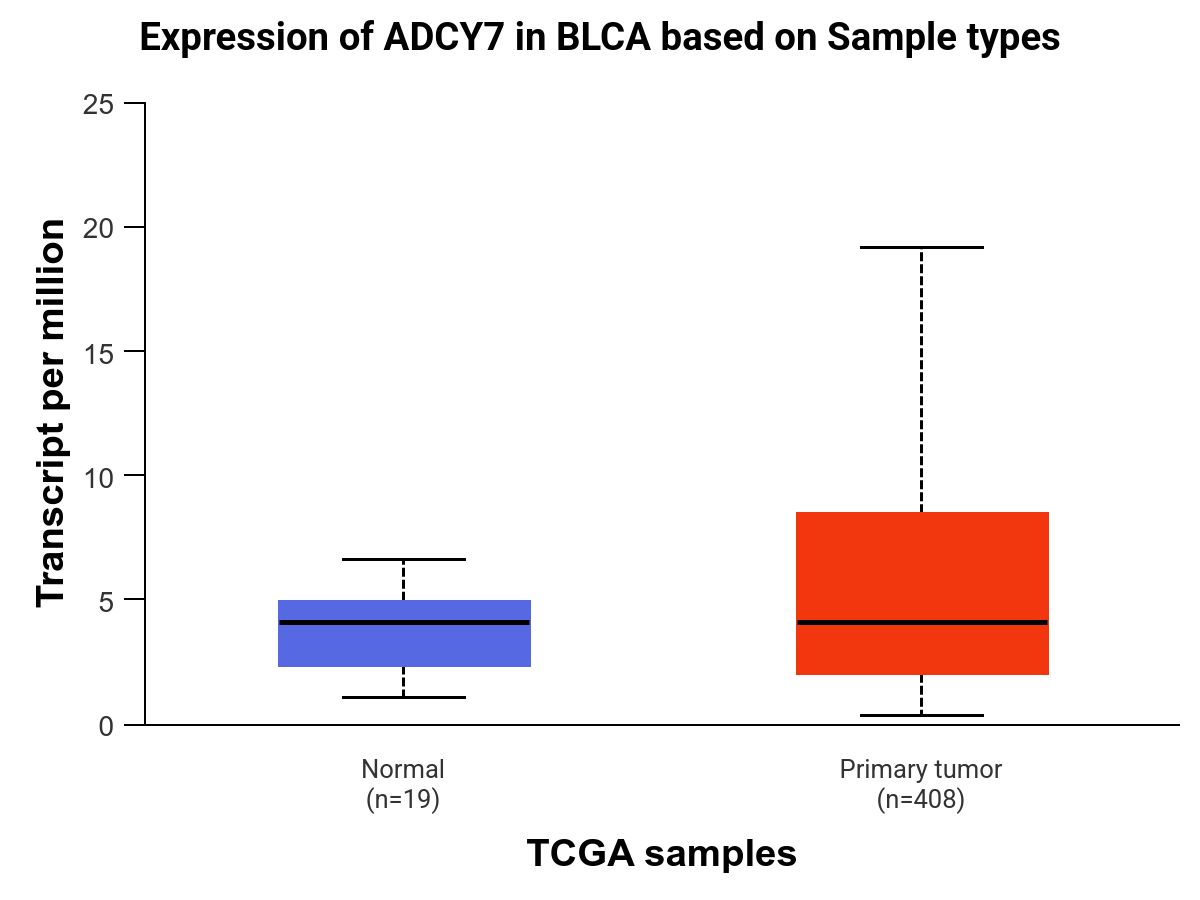

Supplement: Supplementary file 1 [file DataSheet1.zip › all raw data/Figures/Figure 7/Figure 7A.jpg]

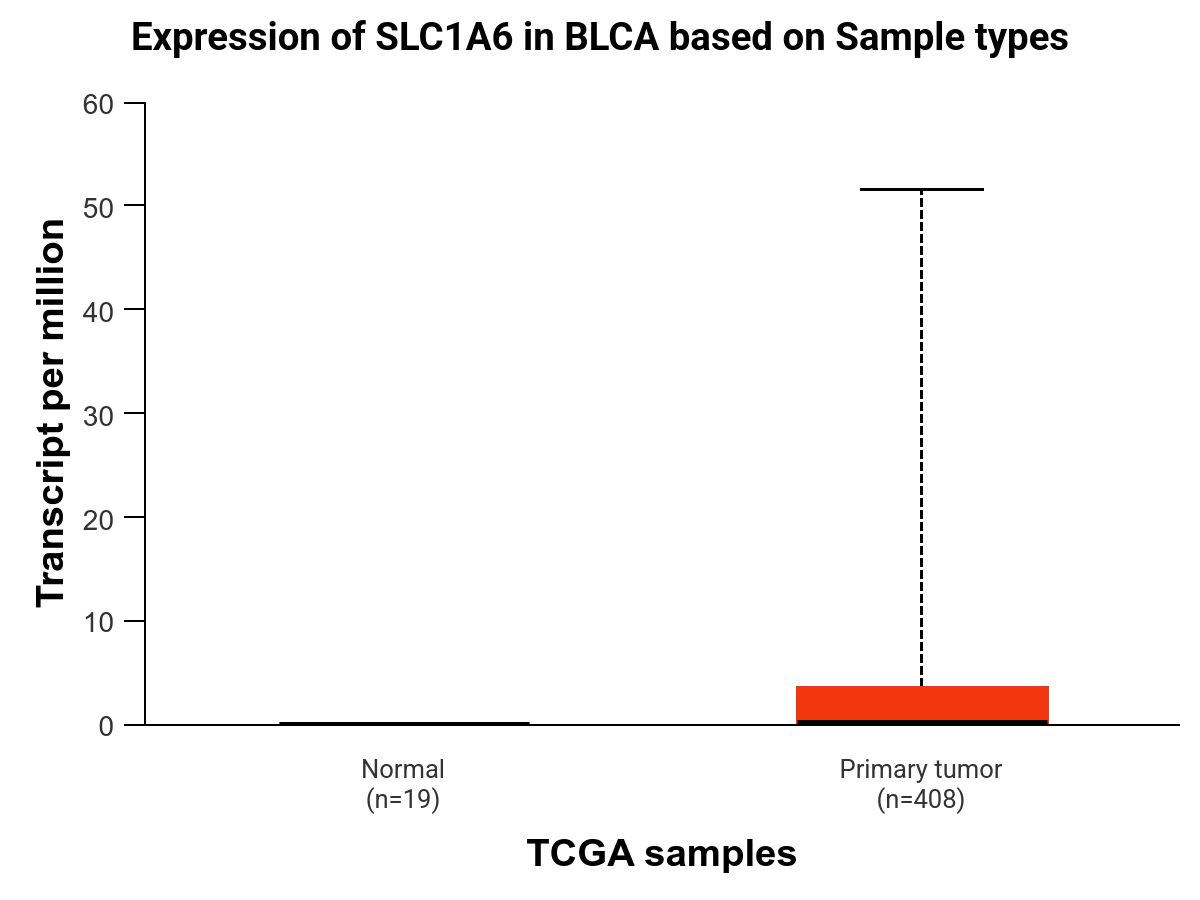

Supplement: Supplementary file 1 [file DataSheet1.zip › all raw data/Figures/Figure 7/Figure 7B.jpg]

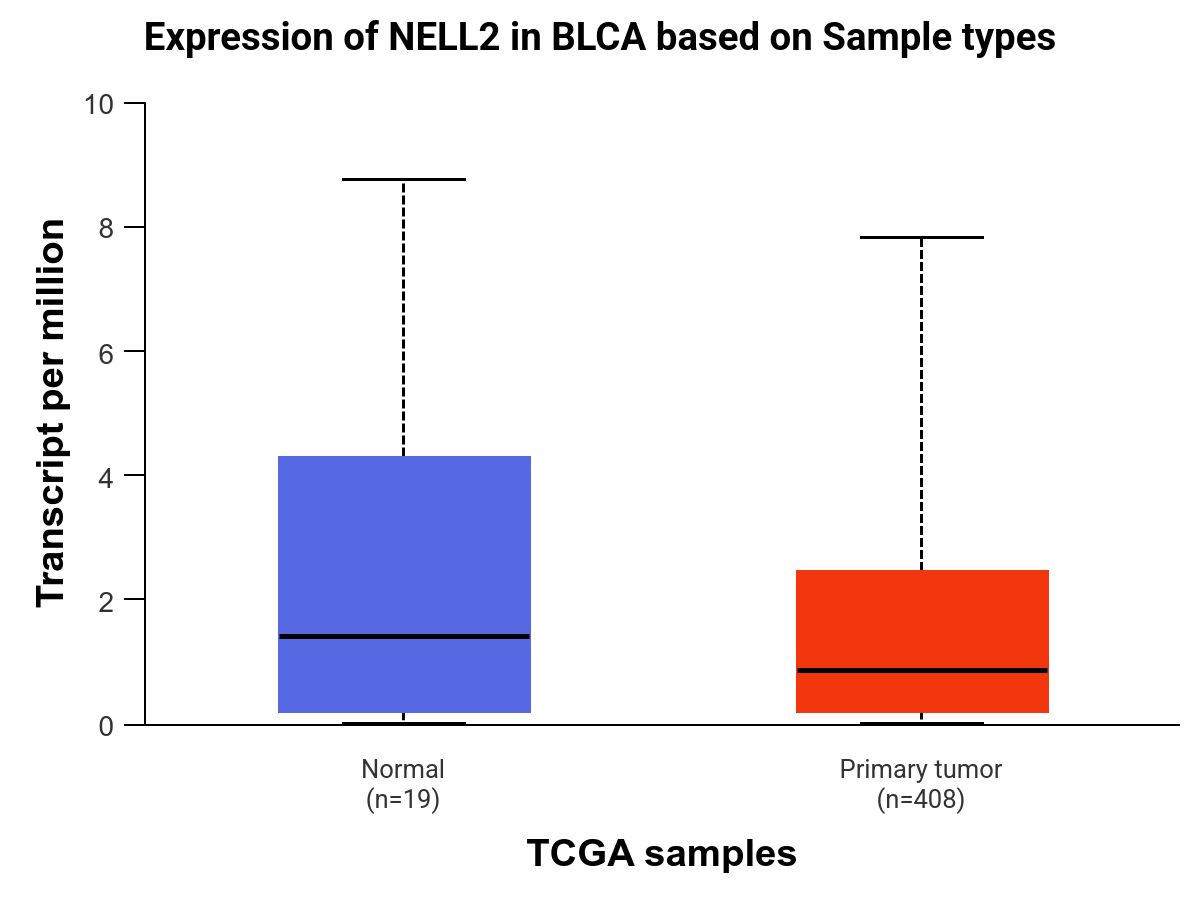

Supplement: Supplementary file 1 [file DataSheet1.zip › all raw data/Figures/Figure 7/Figure 7C.jpg]

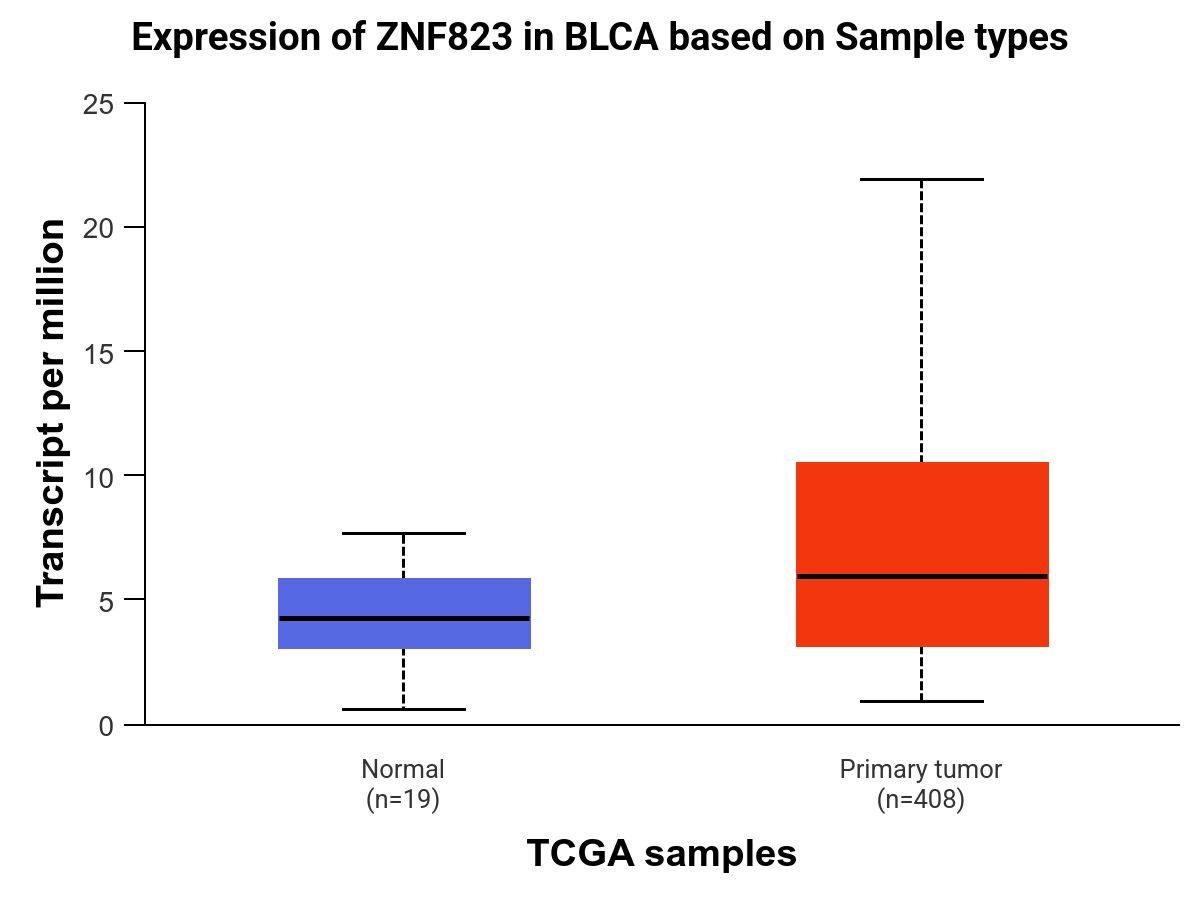

Supplement: Supplementary file 1 [file DataSheet1.zip › all raw data/Figures/Figure 7/Figure 7D.jpg]

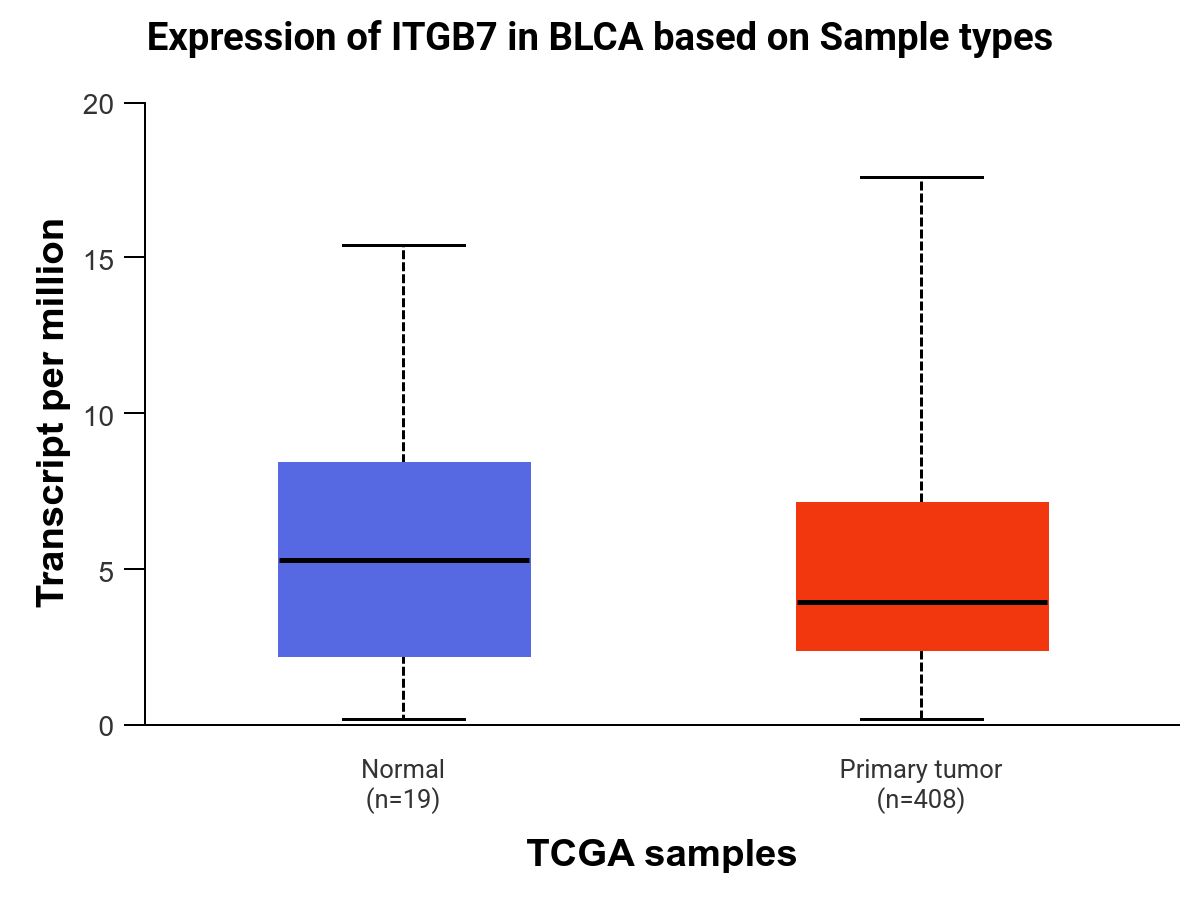

Supplement: Supplementary file 1 [file DataSheet1.zip › all raw data/Figures/Figure 7/Figure 7E.jpg]

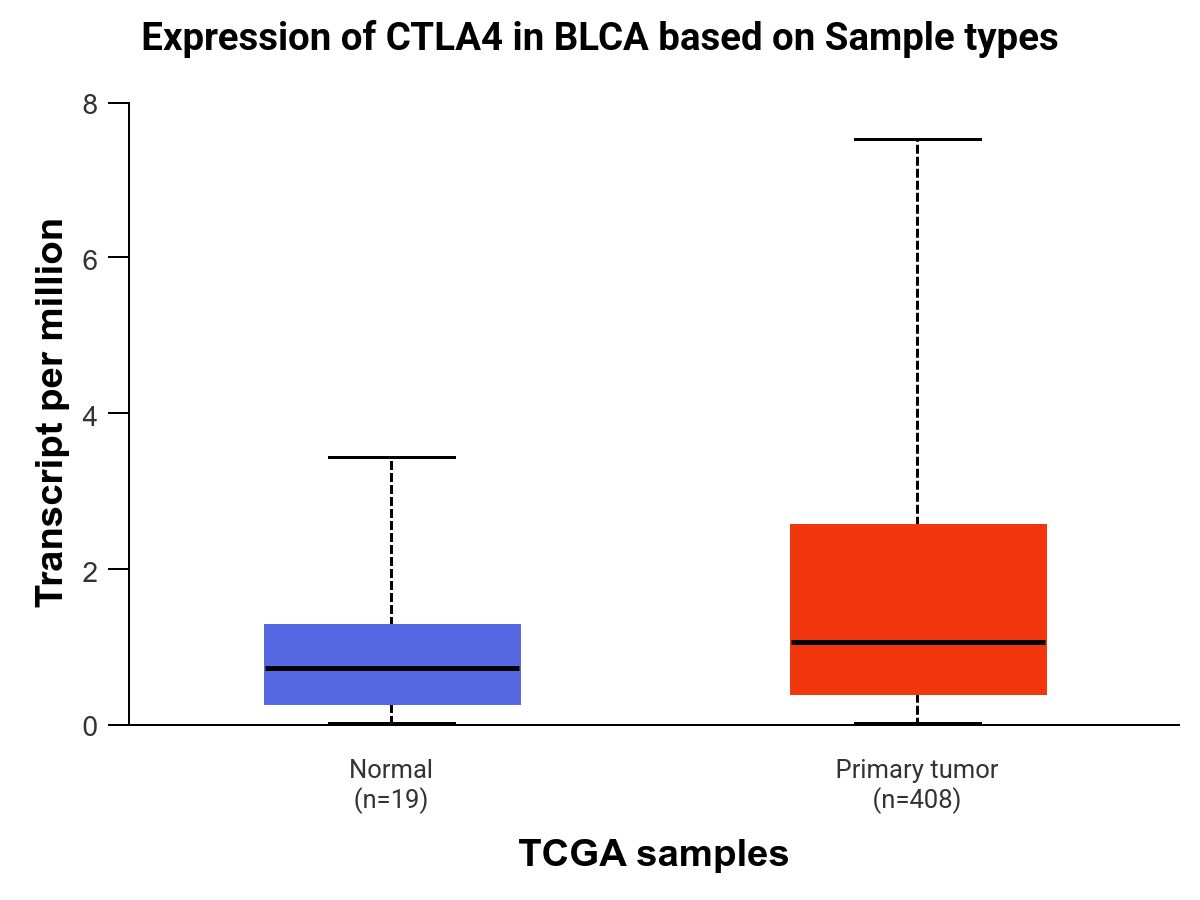

Supplement: Supplementary file 1 [file DataSheet1.zip › all raw data/Figures/Figure 7/Figure 7F.jpg]

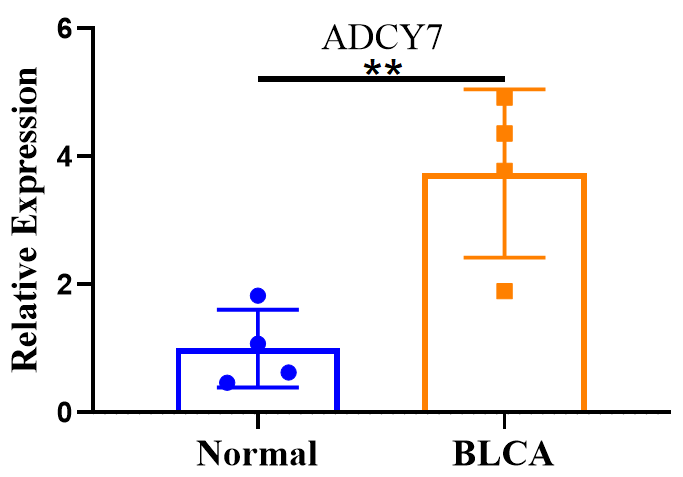

Supplement: Supplementary file 1 [file DataSheet1.zip › all raw data/Figures/Figure 7/Figure 7G.jpg]

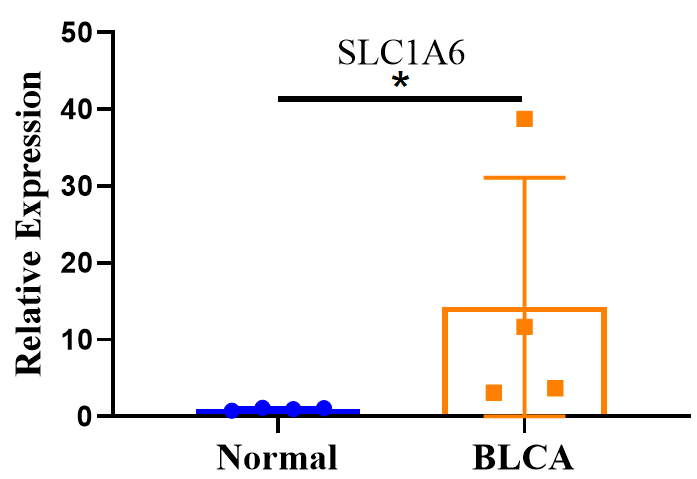

Supplement: Supplementary file 1 [file DataSheet1.zip › all raw data/Figures/Figure 7/Figure 7H.jpg]

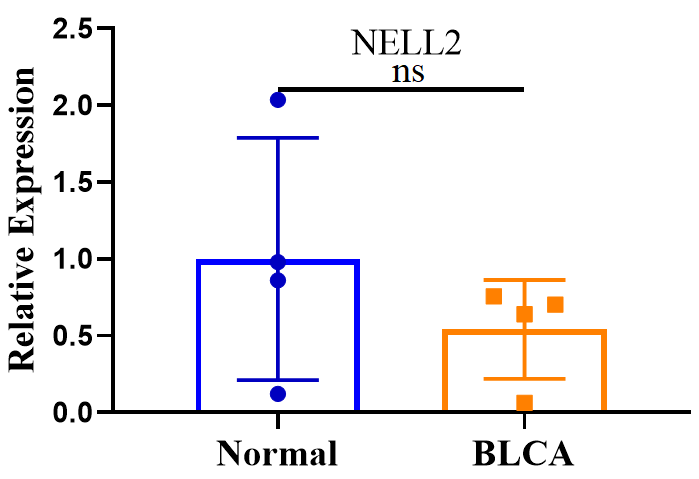

Supplement: Supplementary file 1 [file DataSheet1.zip › all raw data/Figures/Figure 7/Figure 7I.png]

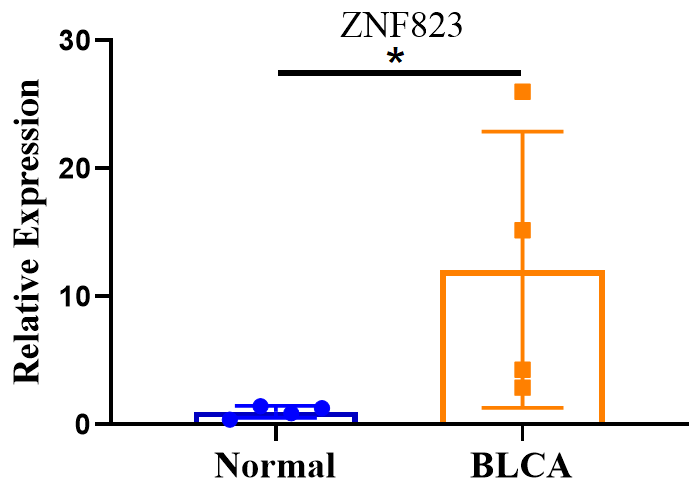

Supplement: Supplementary file 1 [file DataSheet1.zip › all raw data/Figures/Figure 7/Figure 7J.png]

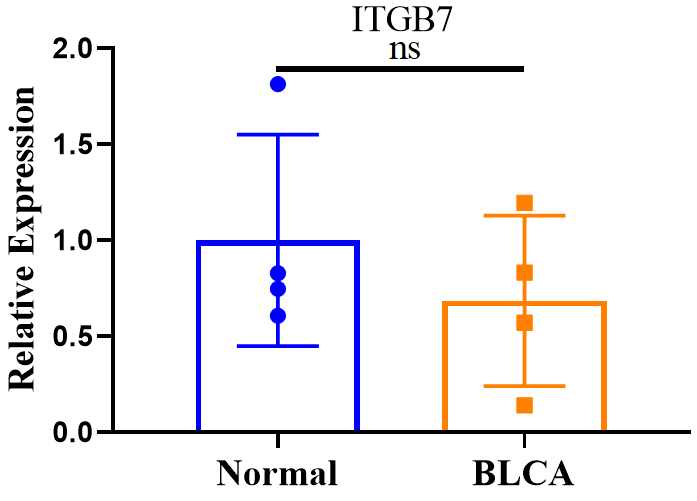

Supplement: Supplementary file 1 [file DataSheet1.zip › all raw data/Figures/Figure 7/Figure 7K.png]

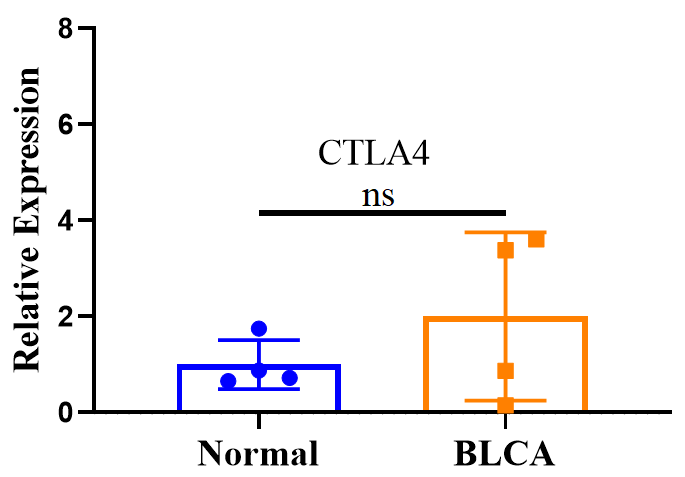

Supplement: Supplementary file 1 [file DataSheet1.zip › all raw data/Figures/Figure 7/Figure 7L.png]

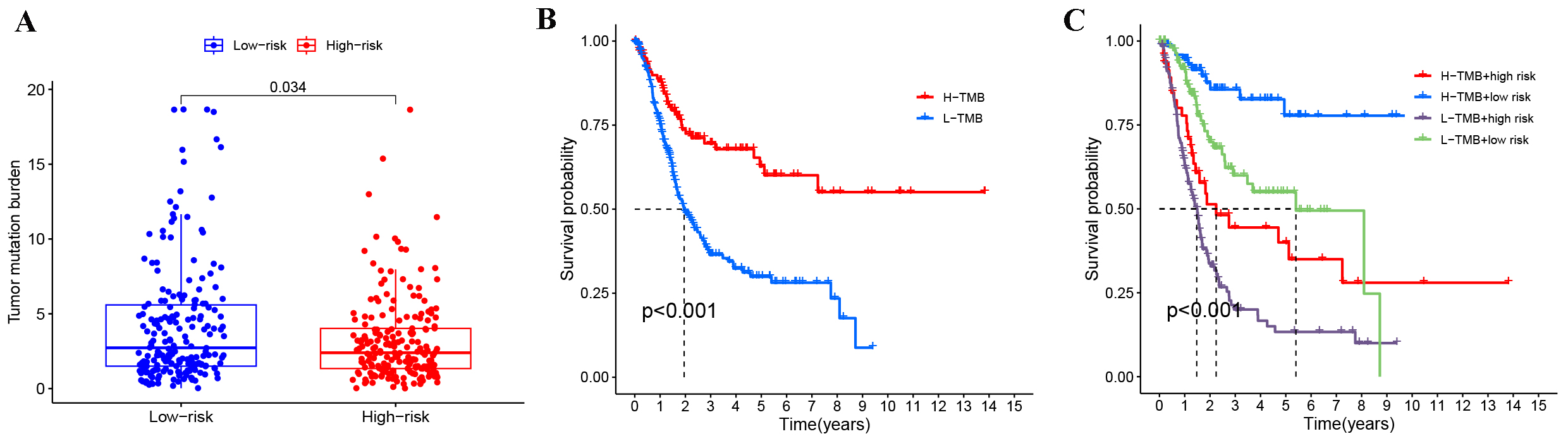

Supplement: Supplementary file 1 [file DataSheet1.zip › all raw data/Figures/Figure 8/Figure 8.jpg]

Low-risk High-risk

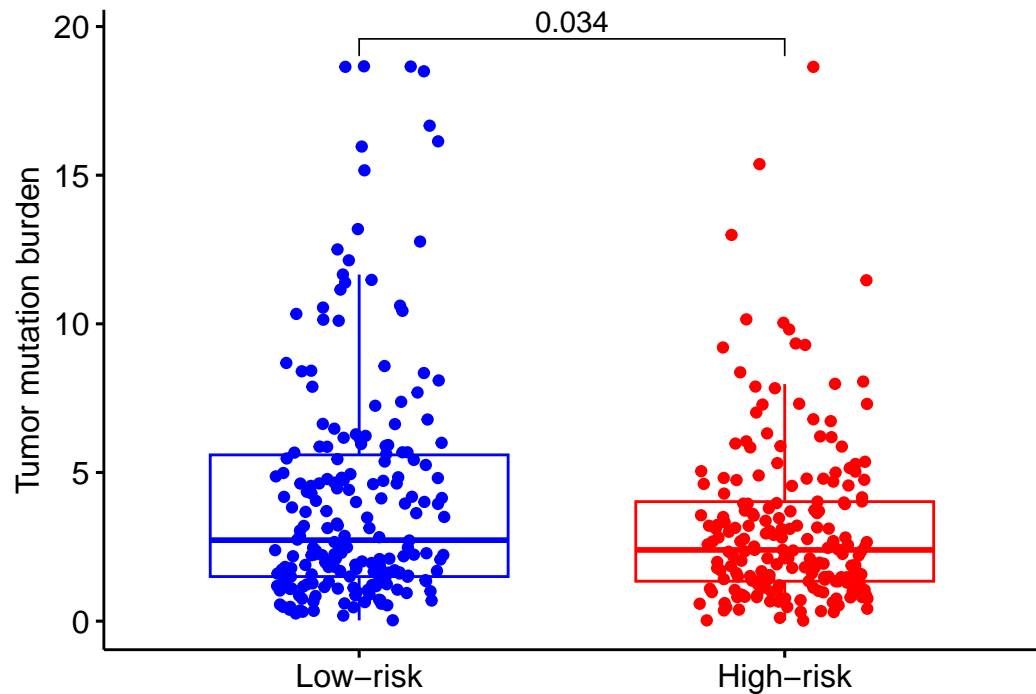

Supplement: Supplementary file 1 [file DataSheet1.zip › all raw data/Figures/Figure 8/Figure 8A.pdf]

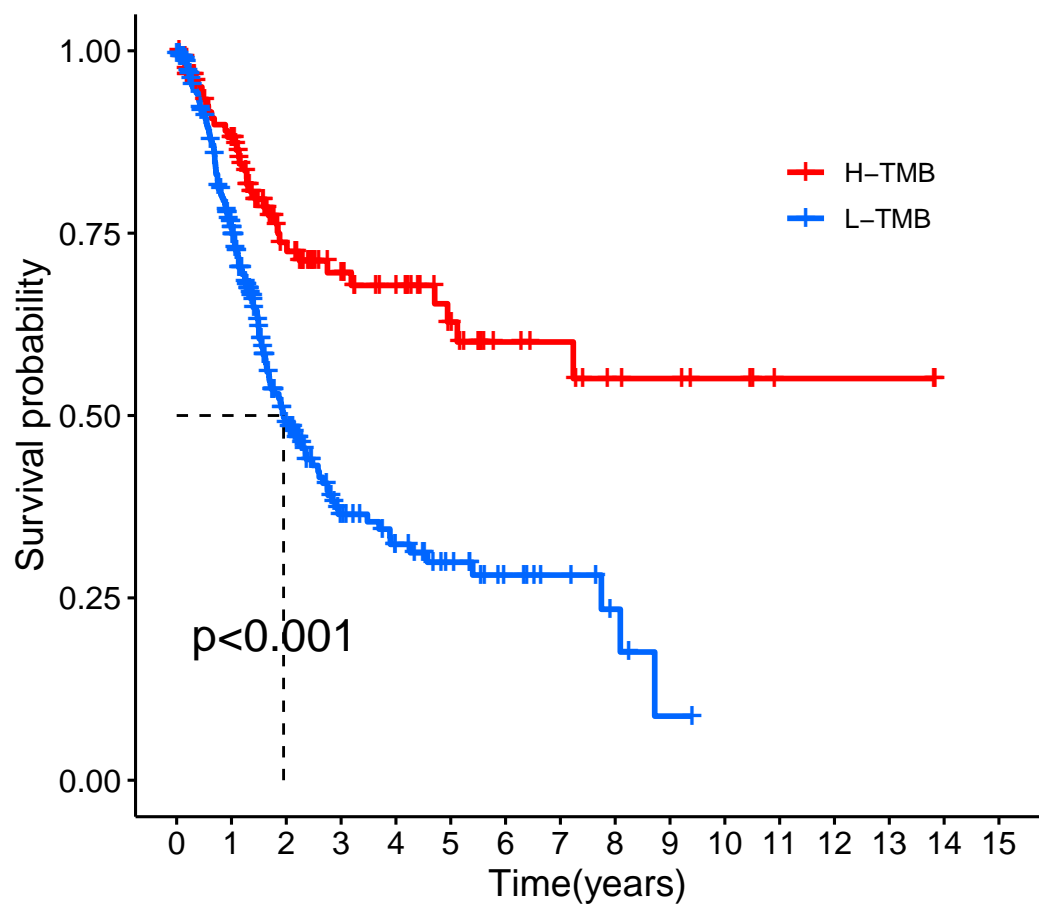

Supplement: Supplementary file 1 [file DataSheet1.zip › all raw data/Figures/Figure 8/Figure 8B.pdf]

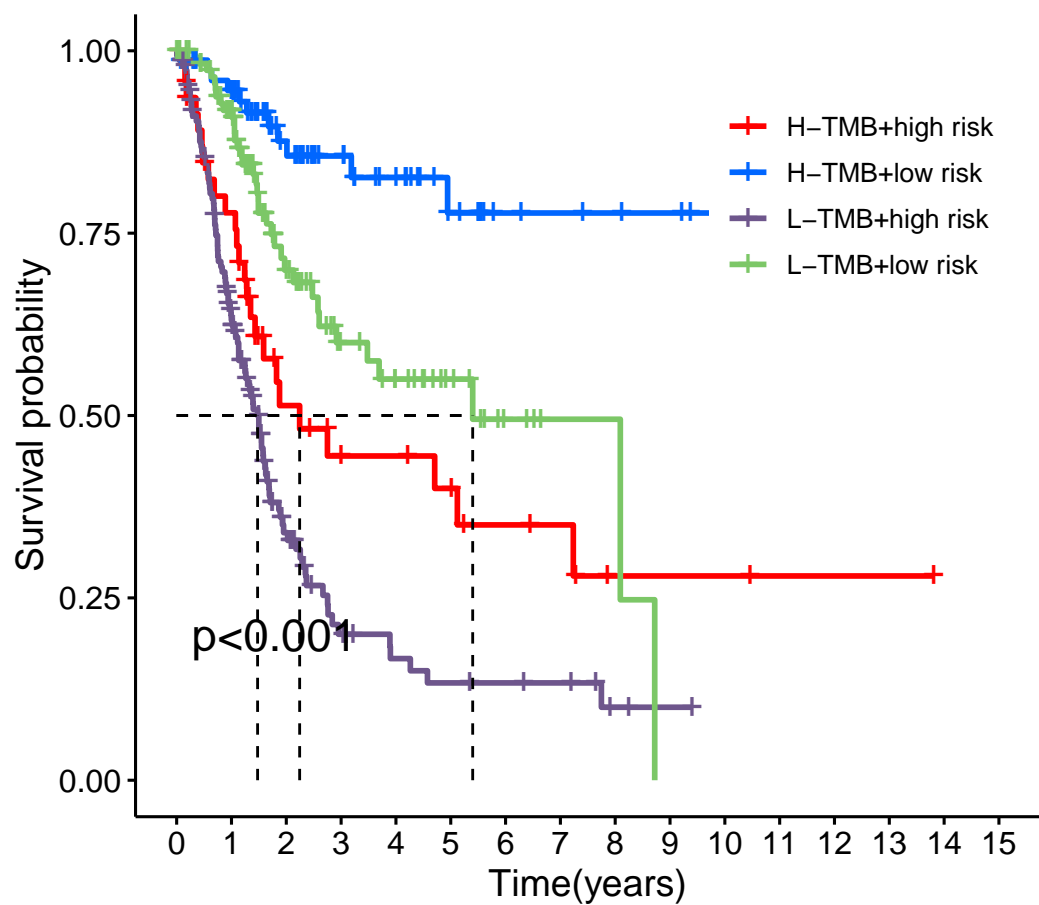

Supplement: Supplementary file 1 [file DataSheet1.zip › all raw data/Figures/Figure 8/Figure 8C.pdf]

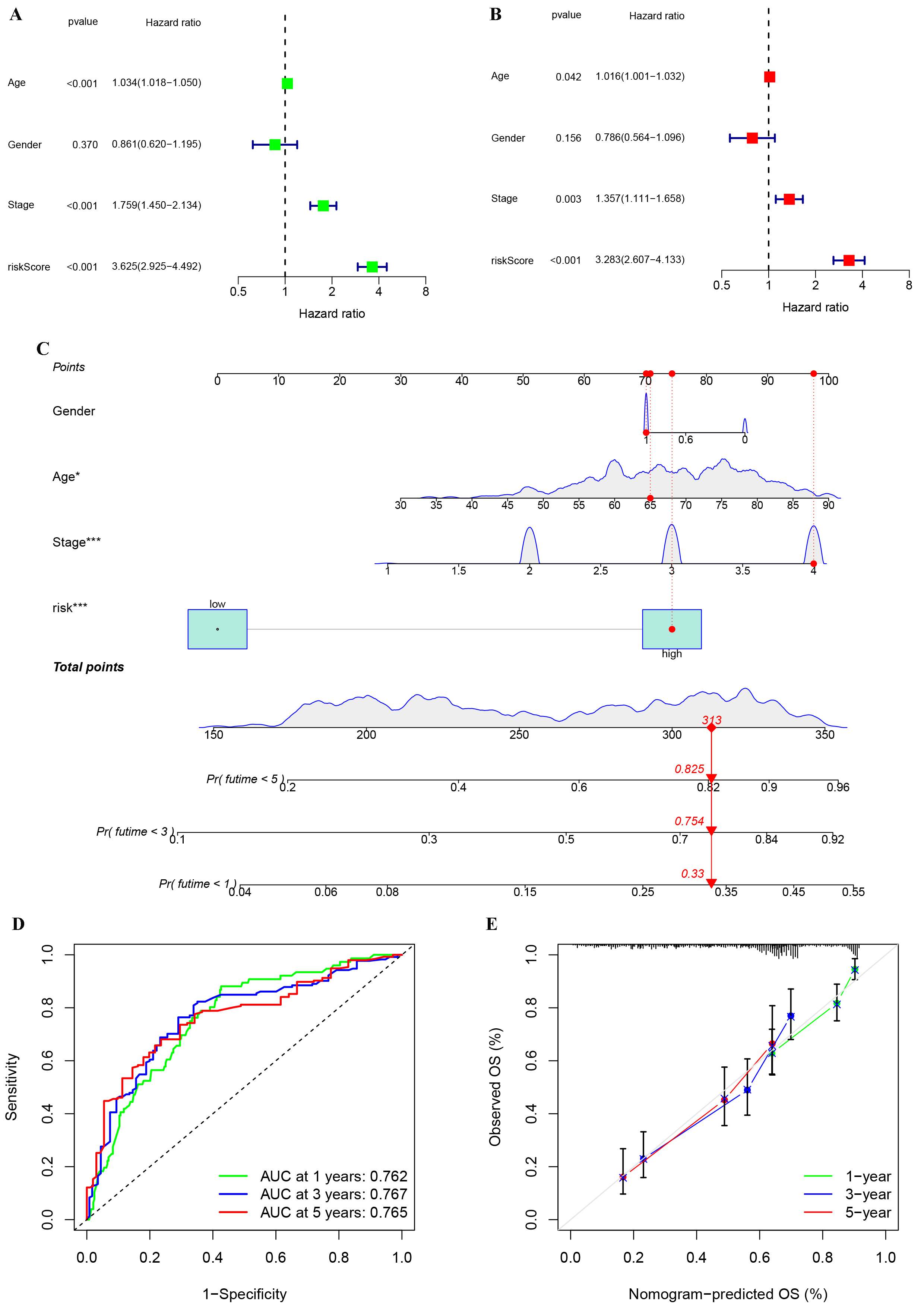

Supplement: Supplementary file 1 [file DataSheet1.zip › all raw data/Figures/Figure 9/Figure 9.jpg]

|           | pvalue | Hazard ratio       |
|-----------|--------|--------------------|
| Age       | <0.001 | 1.034(1.018–1.050) |
| Gender    | 0.370  | 0.861(0.620–1.195) |
| Stage     | <0.001 | 1.759(1.450–2.134) |
| riskScore | <0.001 | 3.625(2.925–4.492) |

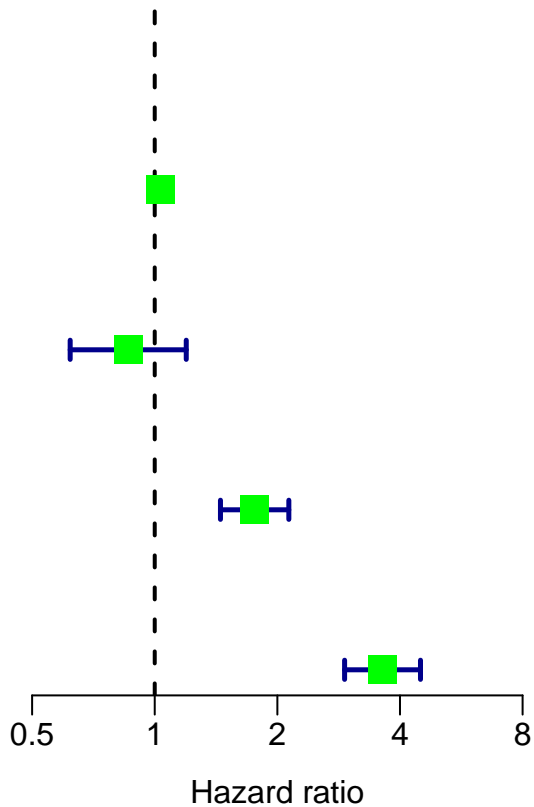

Supplement: Supplementary file 1 [file DataSheet1.zip › all raw data/Figures/Figure 9/Figure 9A.pdf]

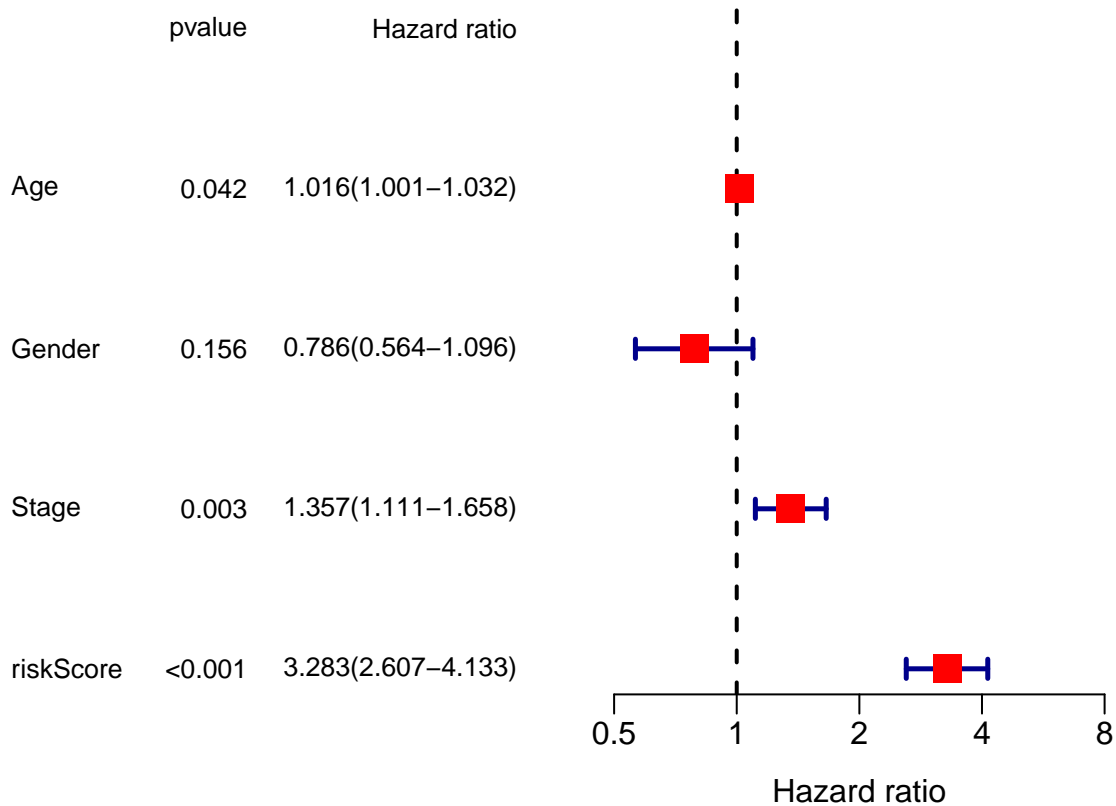

Supplement: Supplementary file 1 [file DataSheet1.zip › all raw data/Figures/Figure 9/Figure 9B.pdf]

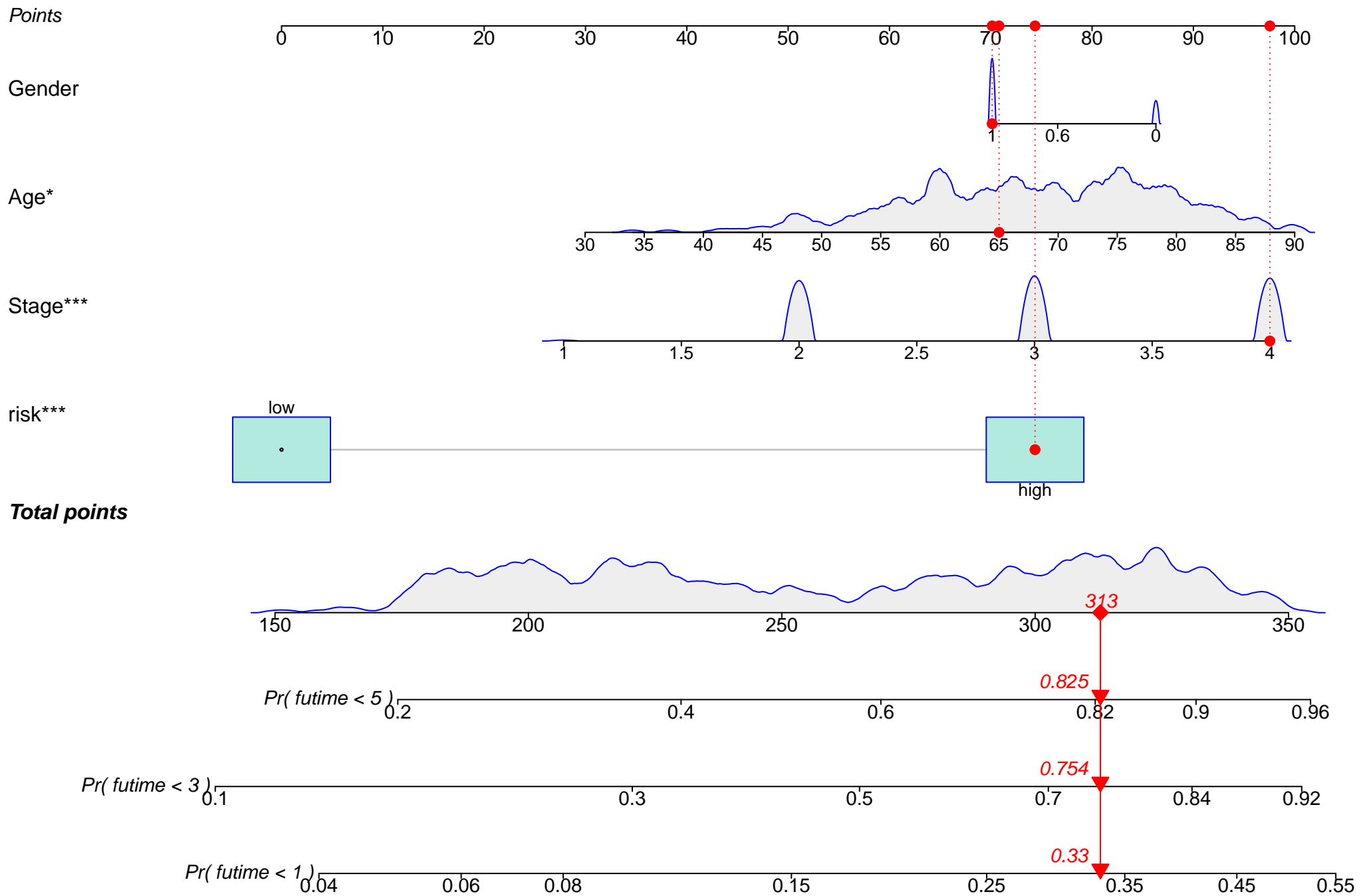

Supplement: Supplementary file 1 [file DataSheet1.zip › all raw data/Figures/Figure 9/Figure 9C.pdf]

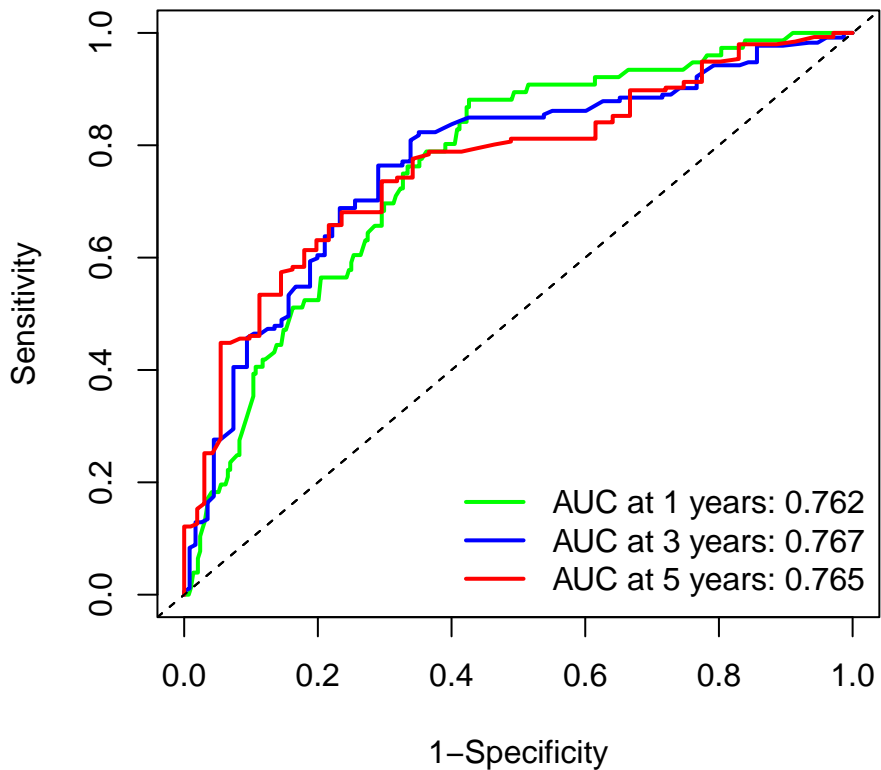

Supplement: Supplementary file 1 [file DataSheet1.zip › all raw data/Figures/Figure 9/Figure 9D.pdf]

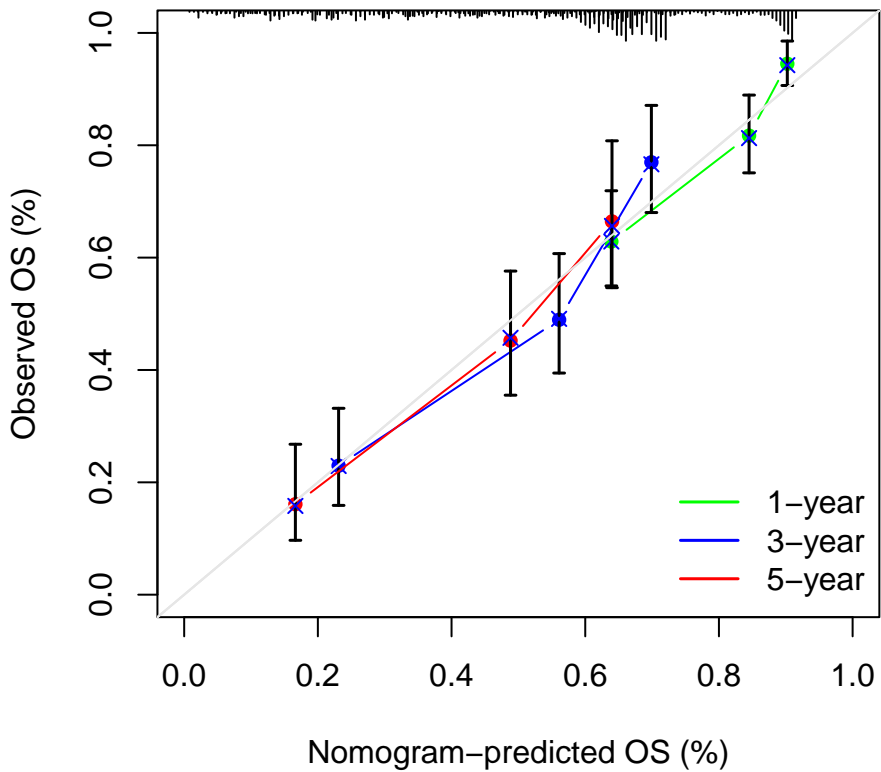

Supplement: Supplementary file 1 [file DataSheet1.zip › all raw data/Figures/Figure 9/Figure 9E.pdf]
