# Supplementary material for: Progressively exploring and assessing the prognosis of bladder urothelial cancer based on the microenvironment through the integration of multiple databases
Source: Front Mol Biosci. 2025 Nov 19;12:1702311. doi: 10.3389/fmolb.2025.1702311 (PMC12672317; doi:10.3389/fmolb.2025.1702311)
Supplement: Supplementary file 1 [file Figure 1B.pdf › all raw data/Figures/Figure 1/Figure 1B.pdf]

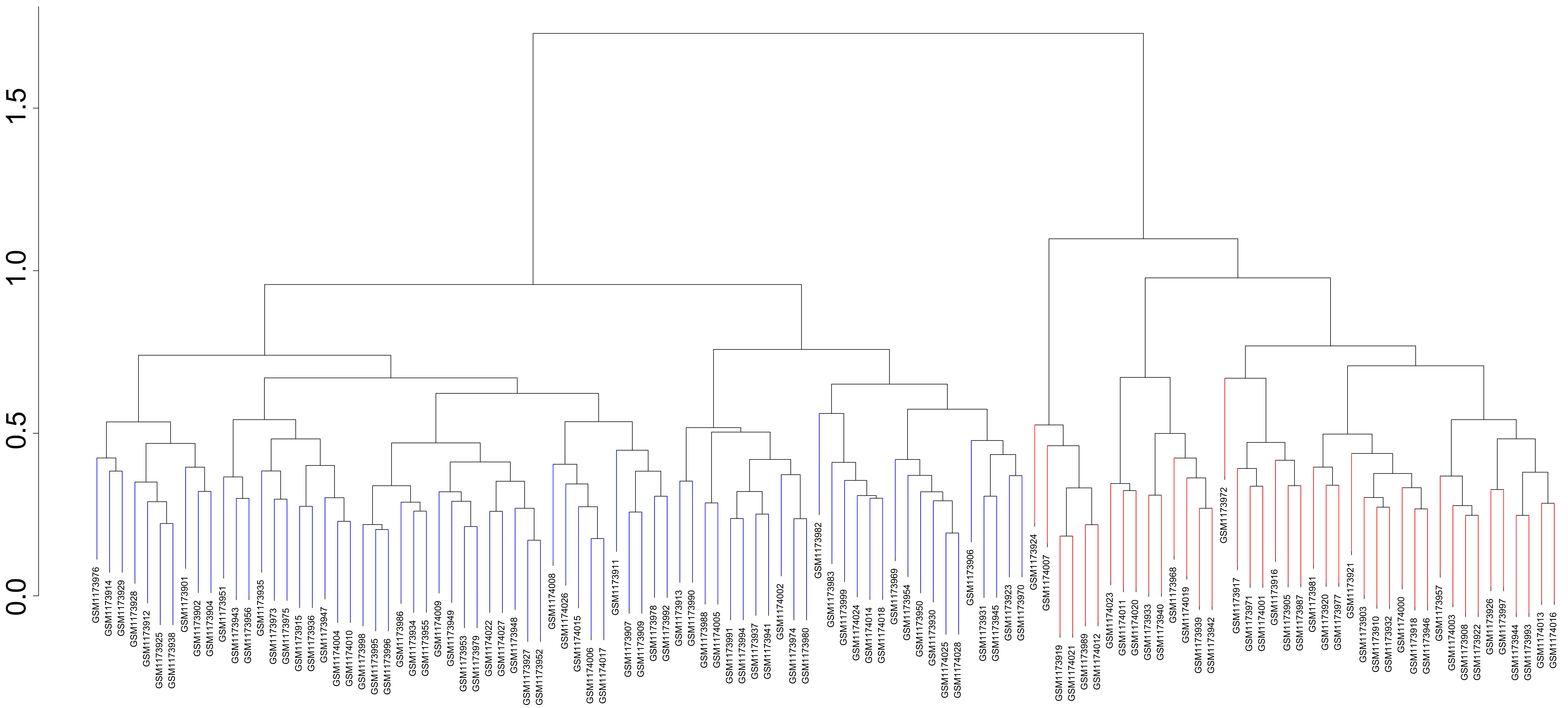

1.5
1.0
0.5
0.0
GSM1173976
GSM1173914
GSM1173929
GSM1173928
GSM1173912
GSM1173925
GSM1173938
GSM1173901
GSM1173902
GSM1173904
GSM1173951
GSM1173943
GSM1173956
GSM1173935
GSM1173973
GSM1173975
GSM1173915
GSM1173936
GSM1173947
GSM1174004
GSM1174010
GSM1173998
GSM1173995
GSM1173996
GSM1173986
GSM1173934
GSM1173955
GSM1174009
GSM1173949
GSM1173953
GSM1173979
GSM1174022
GSM1174027
GSM1173948
GSM1173927
GSM1173952
GSM1174008
GSM1174026
GSM1174015
GSM1174006
GSM1174017
GSM1173911
GSM1173907
GSM1173909
GSM1173978
GSM1173992
GSM1173913
GSM1173990
GSM1173988
GSM1174005
GSM1173991
GSM1173994
GSM1173937
GSM1173941
GSM1174002
GSM1173974
GSM1173980
GSM1173982
GSM1173983
GSM1173999
GSM1174024
GSM1174014
GSM1174018
GSM1173969
GSM1173954
GSM1173950
GSM1173930
GSM1174025
GSM1174028
GSM1173906
GSM1173931
GSM1173945
GSM1173923
GSM1173970
GSM1173924
GSM1174007
GSM1173919
GSM1174021
GSM1173989
GSM1174012
GSM1174023
GSM1174011
GSM1174020
GSM1173933
GSM1173940
GSM1173968
GSM1174019
GSM1173939
GSM1173942
GSM1173972
GSM1173917
GSM1173971
GSM1174001
GSM1173916
GSM1173905
GSM1173987
GSM1173981
GSM1173920
GSM1173977
GSM1173921
GSM1173903
GSM1173910
GSM1173932
GSM1174000
GSM1173918
GSM1173946
GSM1173957
GSM1174003
GSM1173908
GSM1173922
GSM1173926
GSM1173997
GSM1173944
GSM1173993
GSM1174013
GSM1174016
